# Discovery of novel brain permeable and G protein-biased beta-1 adrenergic receptor partial agonists for the treatment of neurocognitive disorders

**DOI:** 10.1371/journal.pone.0180319

**Published:** 2017-07-26

**Authors:** Bitna Yi, Alam Jahangir, Andrew K. Evans, Denise Briggs, Kristine Ravina, Jacqueline Ernest, Amir B. Farimani, Wenchao Sun, Jayakumar Rajadas, Michael Green, Evan N. Feinberg, Vijay S. Pande, Mehrdad Shamloo

**Affiliations:** 1 Department of Neurosurgery, Stanford University School of Medicine, Palo Alto, California, United States of America; 2 Department of Chemistry, Stanford University, Stanford, California, United States of America; 3 Biomaterials and Advanced Drug Delivery Laboratory, Stanford University School of Medicine, Palo Alto, California, United States of America; Texas Tech University, UNITED STATES

## Abstract

The beta-1 adrenergic receptor (ADRB1) is a promising therapeutic target intrinsically involved in the cognitive deficits and pathological features associated with Alzheimer’s disease (AD). Evidence indicates that ADRB1 plays an important role in regulating neuroinflammatory processes, and activation of ADRB1 may produce neuroprotective effects in neuroinflammatory diseases. Novel small molecule modulators of ADRB1, engineered to be highly brain permeable and functionally selective for the G protein with partial agonistic activity, could have tremendous value both as pharmacological tools and potential lead molecules for further preclinical development. The present study describes our ongoing efforts toward the discovery of functionally selective partial agonists of ADRB1 that have potential therapeutic value for AD and neuroinflammatory disorders, which has led to the identification of the molecule STD-101-D1. As a functionally selective agonist of ADRB1, STD-101-D1 produces partial agonistic activity on G protein signaling with an EC_50_ value in the low nanomolar range, but engages very little beta-arrestin recruitment compared to the unbiased agonist isoproterenol. STD-101-D1 also inhibits the tumor necrosis factor α (TNFα) response induced by lipopolysaccharide (LPS) both *in vitro* and *in vivo*, and shows high brain penetration. Other than the therapeutic role, this newly identified, functionally selective, partial agonist of ADRB1 is an invaluable research tool to study mechanisms of G protein-coupled receptor signal transduction.

## Introduction

Adrenergic receptors (ADRs) are G protein-coupled receptors (GPCR) that mediate the central and peripheral effects of noradrenaline (NA) and adrenaline [1, 2]. They are widely expressed throughout the body and play an important role in regulating multiple physiological processes including cardiac muscle contraction, airway reactivity, cognition, arousal, stress-related behavior, and inflammation [2–5]. Multiple subtypes of ADRs exist. Each subtype is expressed in distinct patterns and involved in multiple physiological processes [2, 6]. Therefore, ligands that selectively target one subtype will be valuable both as research tools to identify the roles of different ADR subtypes and as potential therapeutic agents for multiple diseases related to dysfunction of the NA and adrenaline systems.

Among the many subtypes of ADRs, the beta-1 adrenergic receptor (ADRB1) is an important target in multiple therapeutic areas. For example, ADRB1 antagonists (beta-blockers) have been used to treat cardiovascular disease since the 1960s and remain one of the most commonly used drugs today [7, 8]. Our laboratory has also identified therapeutic potential of ADRB1 in the treatment of Alzheimer’s disease (AD). For example, we previously demonstrated that activation of ADRB1 reverses AD-like cognitive deficits in transgenic mice overexpressing human amyloid beta protein precursor (APP) [9]. Similarly, we have shown that acute activation of ADRB1 rescues the contextual memory and spatial memory deficits observed in the Ts65Dn mouse model of Down syndrome, which displays an accelerated AD-like pathology [10]. In addition to the cognitive enhancing effects, we also observed that activation of ADRB1 attenuates pathological features of AD including beta-amyloid burden, tau pathology, and neuroinflammation [11]. Collectively, our discovery suggests that the ADRB1 agonist may have therapeutic potential for AD as it can address both cognitive symptoms and AD pathology. In addition to its involvement in AD, accumulating evidence suggests that the NA system and ADRB1 play a critical role in regulating neuroimmune responses [12–14]. By regulating the neuroinflammatory process, ADRB1 ligands may produce therapeutic benefits for the diseases associated with neuroinflammation. Thus, we have focused our attention on the development of ADRB1 agonists as potential therapeutic agents for AD and neuroinflammatory diseases.

In the classical view of GPCR signaling, activation of ADRB1 leads to stimulation of two ubiquitous and generic mechanisms: G-protein signaling and β-arrestin signaling [15]. Recently, however, it has become clear that agonists can show biased activation of signaling pathways. The ability of ligands to activate a receptor and produce responses in a pathway-dependent manner has been termed “signaling bias” or “functional selectivity” [16, 17]. As G proteins and β-arrestins mediate distinct physiological processes, biased agonists are expected to provide improved therapeutic selectivity with reduced adverse effects.

In search of compounds having therapeutic potential for AD and neuroinflammatory diseases, we sought to identify G protein-biased partial agonists of ADRB1. As partial agonists, these compounds would have more subtle effects in the periphery compared to full agonists, yet be efficacious enough to produce therapeutic benefits for AD and neuroinflammatory disease. By selectively activating ADRB1 G-protein signaling with minimal to no activity on β-arrestin signaling, these compounds would also specifically target the disease relevant signaling pathways without causing the agonist induced tolerance.

**Fig 1:**
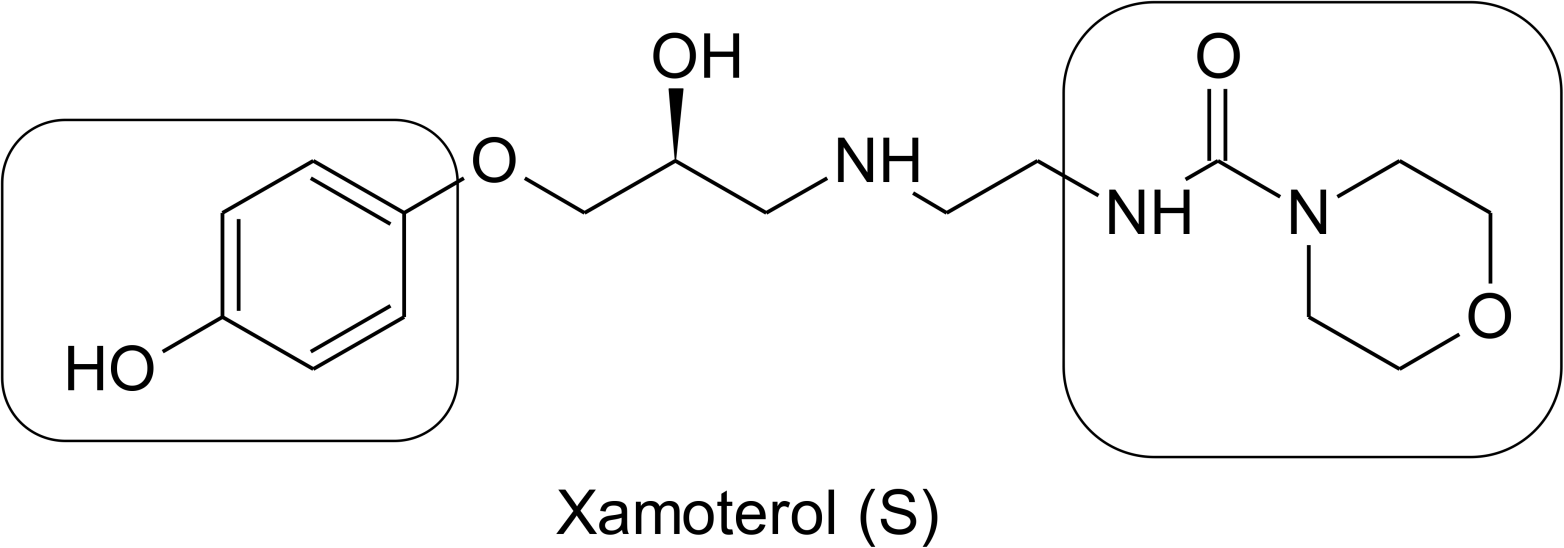
Structure of (S)-xamoterol and two sites of modification.

Xamoterol (ICI118578) is one of the most selective agonists of ADRB1 reported to date (Fig 1). It is a partial agonist that produces approximately 50% efficacy compared to the full agonist isoproterenol, and was once used for the treatment of cardiovascular disease. However, xamoterol is an exceptionally polar compound (clogP = 0.4) with only 5% oral bioavailability in humans [18]. In addition, its central nervous system (CNS) penetration is low due to its polarity. Thus, the focus of our efforts was to develop ADRB1 ligands that (1) are partial agonists of ADRB1, (2) exhibit functional selectivity for the cAMP signaling cascade with minimal or no β-arrestin signaling, and (3) show improved brain penetration, using xamoterol as a lead compound. Here, we report the discovery of a series of compounds, including the highly potent and selective drug-like ADRB1 partial agonist STD-101-D1.

## Materials and methods

### General chemistry and procedure for the preparation of compounds

All compounds were obtained at ≥ 95% purity as determined by HPLC/UV analyses. HPLC analyses were run on an Agilent 1100HPLC using a reverse phase Hypersil GOLD PFP 3μm column with a gradient mobile phase of acetonitrile and water containing 0.01% trifluoroacetic acid. LCMS analyses were run on a Shimadzu LC-10ATvp-API 150EX with an Agilent ZORBAX Eclipse XDB-C18 3.5uM column with a gradient mobile phase of acetonitrile and water containing 0.02% formic acid. Compounds were fully characterized with ^1^HNMR. ^1^HNMR spectra were recorded on a Varian 400 MHz machine. Samples were run in DMSO-*d*6 after removal of the exchangeable protons by D2O exchange with tetramethylsilane as an internal standard. The chemical shifts are expressed in δ (ppm) values, and coupling constants are expressed in hertz (Hz). s = singlet, d = doublet, t = triplet, m = multiplet, and brs = broad singlet. HPLC/UV and ^1^HNMR spectra for all of the compounds are shown in the Supporting Information.

**Fig 2:**
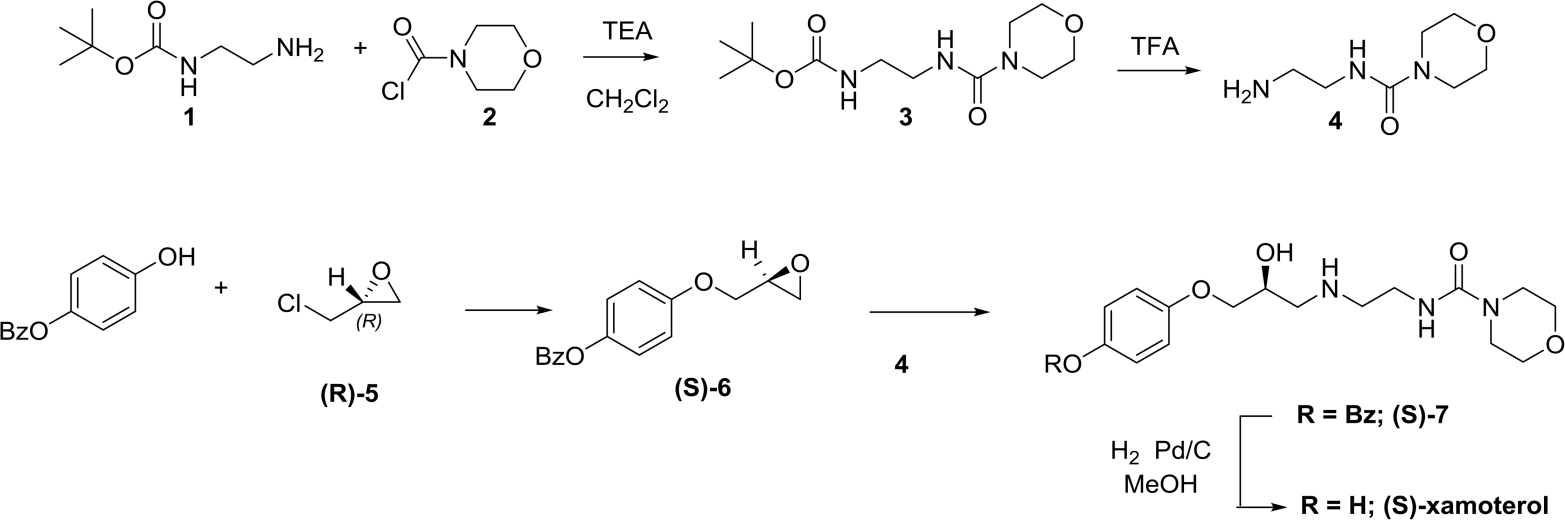
Synthesis of (R)- and (S)-xamoterol.

The synthesis of (R)- and (S)-xamoterol is shown in Fig 2. Reaction of 4-(benzyloxy)phenol with (R)-epichlorohydrin, (R)-5, gave the epoxide (S)-6. Ring opening of this epoxide with the morpholino amine, 4, and reductive removal of the benzyl group with Pd on charcoal then gave (S)-xamoterol. In a similar fashion (R)-xamoterol was prepared using (S)-epichlorohydrin.

#### Experimental procedure for tert-Butyl 2-(morpholine-4-carboxamido)ethylcarbamate (3)

To a solution of tert-butyl 2-aminoethylcarbamate **1** (50 g, 312.09 mmol) in CH_2_Cl_2_ (300mL) was added triethylamine (94.74 g, 936.3 mmol), and morpholine-4-carbonyl chloride **2** (46.7 g, 312.09 mmol) in CH_2_Cl_2_ (200mL) dropwise at 0°C. The reaction mixture was warmed to room temperature and stirred at ambient temperature overnight. The reaction mixture was then concentrated under reduced pressure and, after extractive work up, the residue was purified by silica gel column (CH_2_Cl_2_: MeOH = 10:1) to give tert-butyl 2-(morpholine-4-carboxamido)ethylcarbamate **3** (75g, 87.9%) as a white solid.

#### Experimental procedure for N-(2-aminoethyl) morpholine-4-carboxamide (4)

A solution of tert-butyl 2-(morpholine-4-carboxamido)ethylcarbamate **3** (75 g, 274.39 mmol) in CH_2_Cl_2_ (500mL) was treated dropwise at 0°C with TFA (156.0 g, 1.37 mol). The reaction mixture was stirred for 1.5 hrs and then concentrated under reduced pressure. The resulting residue was dissolved in CH_2_Cl_2_ and 6M NaOH was added to adjust to pH 8. This mixture was extracted with CH_2_Cl_2_ and the organic solution was washed with water, dried over Na_2_SO_4_ and evaporated under reduced pressure to give N-(2-aminoethyl) morpholine-4-carboxamide **4** (43 g, 91%) as a yellow oil. Some of the free base was converted to 4-(N-beta-aminoethylcarbamoyl)morpholine hydrogen sulfate (mp. 168–169°C).

#### Experimental procedure for (S)-2-((4-(benzyloxy)phenoxy)methyl)oxirane ((S)-6)

CsF (113.79 g, 749.14 mmol) was added with stirring to a mixture of 4-(benzyloxy)phenol, (50 g, 249.71 mmol) and (R)-2-(chloromethyl)oxirane, **(R)-5** (69.31 g, 749.14 mmol) in DMF (500mL). The reaction mixture was stirred at 50°C for 3 days. The reaction mixture was then partitioned between water (1 L) and EtOAc (2 L). The organic layer was washed with water (3x900 mL), brine, and then dried over Na_2_SO_4_. The filtered organic extract was concentrated under reduced pressure and purified by silica column (pet ether:EtOAc, 4:1) to give (S)-2-((4-(benzyloxy)phenoxy)methyl)oxirane, **(S)-6** (47 g, 73.4%) as a white solid.

#### Experimental procedure for (S)-N-(2-(3-(4-(benzyloxy)phenoxy)-2-hydroxypropylamino)ethyl)morpholine-4-carboxamide ((S)-7)

A mixture of N-(2-aminoethyl)morpholine-4-carboxamide, **4** (30 g, 104.11 mmol) in isopropanol (120mL) was slowly added to a solution of (S)-2-((4-(benzyloxy)phenoxy)methyl)oxirane, **(S)-6** (19.72 g, 156.66 mmol) in isopropanol (230mL) below 50°C over 5 hrs. The reaction mixture was then cooled to room temperature and thoroughly extracted with ethyl acetate. The organic layer was washed with water, brine, and then dried over Na_2_SO_4_. The ethyl acetate extract was concentrated under reduced pressure and purified by silica gel column (CH_2_Cl_2_: MeOH, 10:1) to give (S)-N-(2-(3-(4-(benzyloxy)phenoxy)-2-hydroxypropylamino)ethyl)morpholine-4-carboxamide, **(S)-7** (20.8 g, 63%) as a white solid.

#### Experimental procedure for (S)-xamoterol

A mixture of (S)-N-(2-(3-(4-(benzyloxy)phenoxy)-2-hydroxypropylamino)ethyl)morpholine-4-carboxamide, **(S)-7** (20 g, 46.56 mmol) in 200mL of EtOH containing 2mL of acetic acid was added Pd(OH)_2_/C (5g). The resulting reaction mixture was subjected to hydrogenation at 55 psi overnight. The catalyst was removed by filtration and the filtrate was concentrated under reduced pressure. The residue was dissolved in EtOH, and converted to hemifumarate with fumaric acid. The hemifumarate salt was recrystallized with EtOH to give **(S)-xamoterol hemifumarate** (12.3 g, 91.7%) as an off-white solid.

#### Experimental procedure for (R)-2-((4-(benzyloxy)phenoxy)methyl)oxirane ((R)-6)

This compound was prepared following the procedure as described for **(S)-6** from **4-(benzyloxy)phenol** (8 g, 41.9 5mmol) and (S)-2-(chloromethyl)oxirane, **(R)-5**, to give (R)-2-((4-(benzyloxy)phenoxy)methyl)oxirane, **(R)-6** (6.3 g, 24.6 mmol, 61%) as a white solid.

#### Experimental procedure for (R)-N-(2-(3-(4-(benzyloxy)phenoxy)-2-hydroxypropylamino)ethyl)morpholine-4-carboxamide ((R)-7)

This compound was prepared following the procedure as described for the preparation of **(S)-7** from 6.3 g, (24.6 mmol) of **(R)-6** and 12.77 g (73.80 mmol) of **4** to afford 3 g, (6.98 mmol, 30%) of **(R)-7**.

#### Experimental procedure for (R)-xamoterol

**(R)-7** (3g, 6.98mmol) was converted to **(R)-xamoterol hemifumarate** following the procedure as described above to give **(R)-xamoterol hemifumurate** (2.5g, 6.8mmol) as an off-white solid.

**Fig 3:**
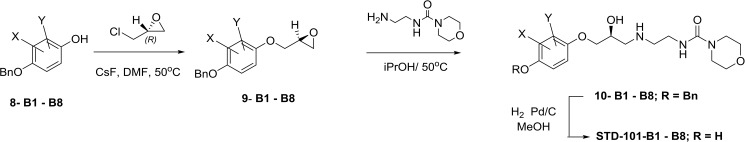
General method for the synthesis of compounds STD-101-B1 to B8.

The general method for the synthesis of compounds **STD-101-B1** to **B8** is shown in Fig 3. Reaction of phenols **8**-B1 to B8 with (R)-2-(chloromethyl)oxirane and CsF in iPrOH for 48 hrs at 50°C gave the coupled epoxides **9**-B1 to B8 in good yield. Epoxide opening with an excess of N-(2-aminoethyl)morpholine-4-carboxamide in iPrOH at 50°C then provided the benzyl ethers **10**-B1 to B8. Reductive removal of the benzyl protecting group with Pd/C then gave the free phenols, **STD-101-B1** to **B8** and **E** which were isolated either as the hemifumarate salts, **STD-101-B1, B2, B3, B5** and **B7** or as the free base, **STD-101-B4, B6**, **B8** and **E**. Experimental procedure for the representative example STD-101-B1 is shown below.

#### Experimental procedure for (S)-2-((4-(benzyloxy)-3-methylphenoxy)methyl)oxirane (9-B1)

To a solution of 4-(benzyloxy)-3-methylphenol (1.95 g, 9.10 mmol) in DMF (20 mL) was added (R)-2-(chloromethyl)oxirane (2.53 g, 27.30 mmol),CsF (4.15 g, 27.30 mmol). The reaction mixture was stirred for 48 hrs at 50°C. The reaction mixture was cooled to room temperature and poured to ice water and extracted with EtOAc (3X20 mL).The combined organic layers were washed with brine, dried over Na_2_SO_4_, filtered, concentrated under reduced pressure and purified by flash silica column chromatography to give (S)-2-((4-(benzyloxy)-3-methylphenoxy)methyl)oxirane (2.1 g, 84.5%) as a yellow oil.

#### Experimental procedure for (S)-N-(2-(3-(4-(benzyloxy)-3-methylphenoxy)-2-hydroxypropylamino)ethyl)morpholine-4-carboxamide (10-B1)

To a solution of (S)-2-((4-(benzyloxy)-3-methylphenoxy)methyl)oxirane (0.6 g, 2.22 mmol) in iPrOH (20 mL) was added N-(2-aminoethyl)morpholine-4-carboxamide (0.77 g, 4.44 mmol). The reaction mixture was stirred for 18 hrs at 50°C. The reaction mixture was cooled to room temperature and poured to ice water and extracted with EtOAc (3X20 mL). The combined organic layers were washed with brine, dried over Na_2_SO_4_, filtered, concentrated under reduced pressure and purified by flash silica column chromatography to give (S)-N-(2-(3-(4-(benzyloxy)-3-methylphenoxy)-2-hydroxypropylamino)ethyl)morpholine-4-carboxamide (0.5 g, 50.8%) as a yellow oil.

#### Experimental procedure for STD-101-B1, hemifumarate

To a solution of (S)-N-(2-(3-(4-(benzyloxy)-3-methylphenoxy)-2-hydroxypropylamino)ethyl)morpholine-4-carboxamide (0.5 g, 2.22 mmol) in MeOH(15 mL) was added Pd/C. The reaction mixture was subjected to H_2_ for 4 hrs. The reaction mixture was filtered, and the filtrate was concentrated under reduced pressure. The residue was dissolved in EtOH(15 mL), fumaric acid(0.5eq) was added and solvent removed under reduced pressure to give STD-101-B1, hemifumarate (0.35 g, 75.5%) as a pink solid.

**Fig 4:**
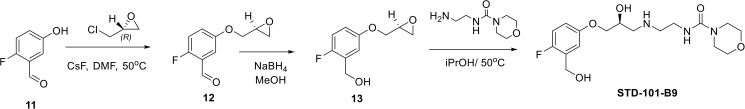
Synthesis of STD-101-B9.

The synthesis of **STD-101-B9** is shown in Fig 4. Reaction of 2-fluoro-5-hydroxybenzaldehyde with (R)-2-(chloromethyl)oxirane and CsF in iPrOH gave the coupled epoxide **12,** which was then treated with sodium borohydride to reduce the aldehyde to the hydroxymethylene, **13.** Ring opening with N-(2-aminoethyl) morpholine-4-carboxamide gave **STD-101-B9**, which was isolated as the hemifumarate salt.

#### Experimental procedure for (S)-2-fluoro-5-(oxiran-2-ylmethoxy)benzaldehyde (12)

A mixture of 2-fluoro-5-hydroxybenzaldehyde (1.0 g, 7.14 mmol), (R)-2-(chloromethyl) oxirane (1.98 g, 21.41mmol) and CsF(3.25 g,21.41mmol) in DMF (10 ml) was stirred at 0°C and then heated at 50°C for 48 hrs. The reaction mixture was then portioned between water and ethylacetate (2:1 ratio), the organic layer was washed with water, brine, dried over Na_2_SO_4_, filtered, concentrated under reduced pressure and purified by silica column chromatography, to give (S)-2-fluoro-5-(oxiran-2-ylmethoxy)benzaldehyde (1.1 g, 78.6%) as a light yellow oil.

#### Experimental procedure for (S)-(2-fluoro-5-(oxiran-2-ylmethoxy)phenyl)methanol (13)

To a solution of (S)-2-fluoro-5-(oxiran-2-ylmethoxy)benzaldehyde (1.2 g, 6.14 mmol) in THF (10 ml) was added NaBH_4_(0.23 g, 6.12 mmol) and the reaction mixture was stirred at room temperature overnight. Then the reaction mixture was quenched with water and extracted with EtOAc (3X30mL). The combined organic layers were washed with water, brine, dried over Na_2_SO_4_, filtered, concentrated under reduced pressure and purified by silica column chromatography, to give (S)-(2-fluoro-5-(oxiran-2-ylmethoxy)phenyl)methanol (0.57 g, 47%) as yellow oil.

#### Experimental procedure for (S)-N-(2-(2-hydroxy-3-(3-methyl-1H-indazol-4-yloxy)propylamino)ethyl)morpholine-4 (STD-101-B9)

To a solution of (S)-(2-fluoro-5-(oxiran-2-ylmethoxy) phenyl) methanol (0.57 g, 2.88mmol) in iPrOH (20 ml), was added N-(2-aminoethyl) morpholine-4-carboxamide (1.0g, 5.75mmol). The reaction mixture was stirred at 50°C for 5 hrs, and then evaporated under reduced pressure. Water was added to the residue and the product was extracted into ethyl acetate. The organic layer was washed with water, brine, dried over Na_2_SO_4_, filtered, concentrated under reduced pressure and purified by silica column chromatography to give (S)-N-(2-(2-hydroxy-3-(3-methyl-1H-indazol-4-yloxy)propylamino)ethyl)morpholine-4-carboxamide (0.23 g, 18.6%) as a solid.

**Fig 5:**
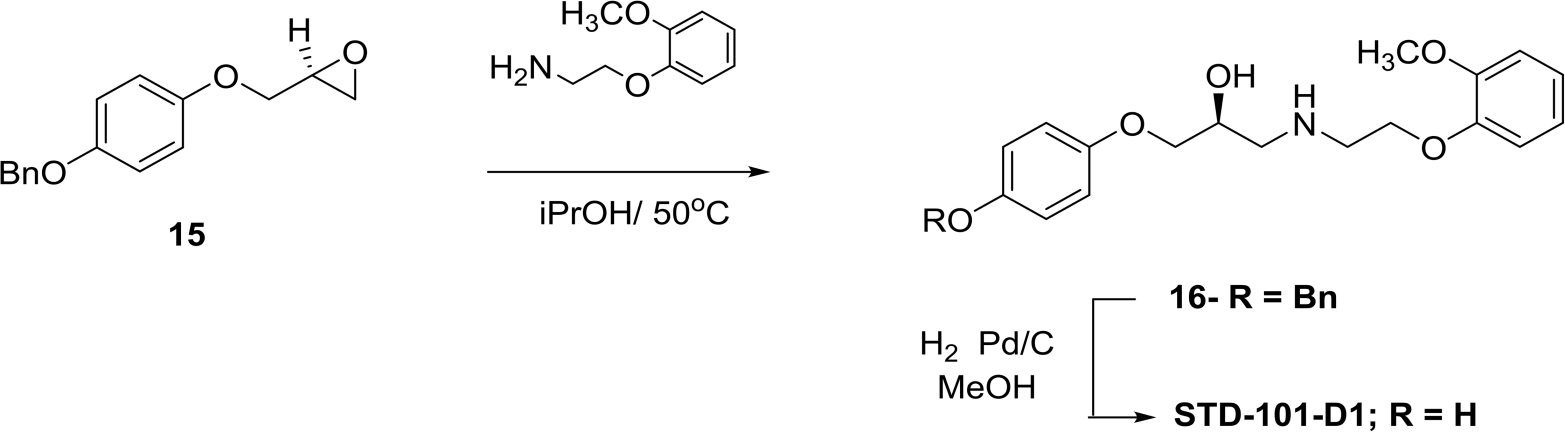
General method for the synthesis of compounds STD-101-D1 to D6.

The general method for the synthesis of compounds **STD-101-D1** to **D6** is shown in Fig 5. The starting material (S)-1-(4-(benzyloxy)phenoxy)-3-(2-(2-methoxyphenoxy)ethylamino)propan-2-ol (**15**) was prepared by treating 4-benzyloxyphenol with (R)-epichlorohydrin as described above. Opening of the epoxide ring of **15** with 2-(2-methoxyphenoxy)ethanamine gave the benzyl ether **16** which was then deprotected by catalytic reduction to give the desired product **STD-101-D1**. Compounds **STD-101-D2** to **D6** were prepared in a similar fashion using the appropriate amine reagent and isolated as the hemifumarate salts. Experimental procedure for the representative example compound STD-101-D1 is shown below.

#### Experimental procedure for (S)-1-(4-(benzyloxy)phenoxy)-3-(2-(2-methoxyphenoxy)ethylamino)propan-2-ol (16)

To a solution of (S)-2-((4-(benzyloxy)phenoxy)methyl)oxirane (1.0 g, 3.9mmol) in iPrOH (40 ml), was added 2-(2-methoxyphenoxy)ethanamine (0.98 g, 5.8mmol) in iPrOH dropwise under 50°C for 5h. The reaction mixture was cooled to room temperature, and removed the solution, water was added, extracted with EA. The organic layer was washed with water, brine, dried over Na_2_SO_4_, concentrated under reduced pressure and purified by silica column, to give (S)-1-(4-(benzyloxy)phenoxy)-3-(2-(2-methoxyphenoxy)ethylamino)propan-2-ol (0.52 g, 1.228mmol).

#### Experimental procedure for (S)-4-(2-hydroxy-3-(2-(2-methoxyphenoxy)ethylamino)propoxy)phenol (STD-101-D1)

A solution of (S)-1-(4-(benzyloxy)phenoxy)-3-(2-(2-methoxyphenoxy)ethylamino)propan-2-ol (0.52 g, 1.2mmol) and Pd/C (0.1 g) in MeOH (20ml), was stirred under a H_2_ atmosphere at RT for 2h. The reaction mixture was filtered, concentrated under reduced pressure, and purified by silica column to give (S)-4-(2-hydroxy-3-(2-(2-methoxyphenoxy)ethylamino)propoxy)phenol (0.35g, 1.05mmol).

**Fig 6:**
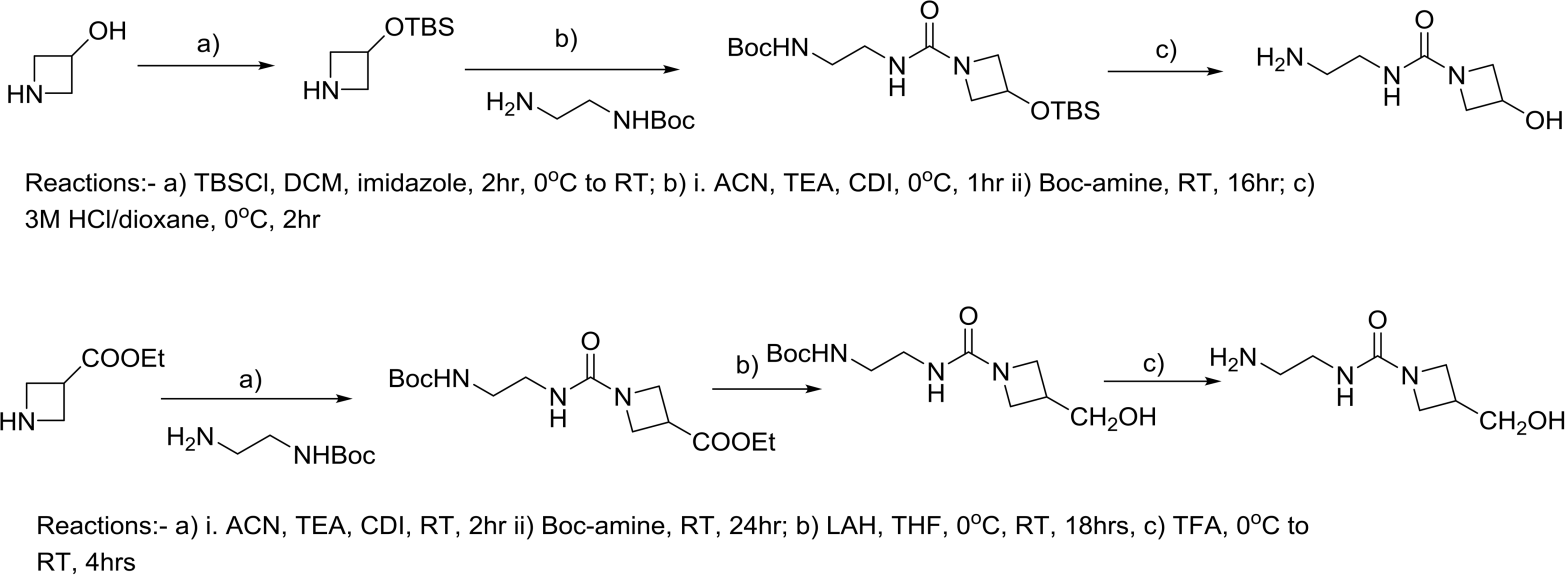
Syntheses of the amine components for STD-101-D5 and D6.

The syntheses of the amine components for **STD-101-D5** and **D6** are shown in Fig 6.

### NMR spectral data

NMR spectral results for STD-101-B1 to B9, D1 to D6 and E are shown below. It is important to highlight that the H-signals for the fumaric acid in the hemifumarate salts are not recorded.

#### (S)-N-(2-((2-hydroxy-3-(4-hydroxy-3-methylphenoxy)propyl)amino)ethyl)morpholine-4-carboxamide hemifumarate (STD-101-B1)

MS(m/z), 354(M+1); ^1^HNMR (400 MHz, DMSO, after D2O): 2.04 (3H, s), 2.91(2H, m), 3.06(1H, m), 3.21 (4H, m), 3.25 (1H,m), 3.50(4H, m), 3.75 (1H,m), 4.0 (1H, m), 6.5 (1H,s), 6.66(2H, m).

#### (S)-N-(2-((3-(3-chloro-4-hydroxyphenoxy)-2-hydroxypropyl)amino)ethyl)morpholine-4-carboxamide hemifumarate (STD-101-B2)

MS(m/z), 374(M+1); ^1^HNMR (400 MHz, DMSO, after D2O exchange, whenever as salt signals for fumaric acid are not shown): 2.96 (4H, m), 3.25 (4H, m), 3.37 (2H, m), 3.50 (4H, m), 3.84 (2H, m), 4.08 (1H, m), 6.73 (1H, dd, J = 8,4Hz); 6.76 (1H, d, J = 8Hz), 6.94 (1H, d, J = 4Hz.)

#### (S)-N-(2-((3-(3-ethyl-4-hydroxyphenoxy)-2-hydroxypropyl)amino)ethyl)morpholine-4-carboxamide hemifumarate (STD-101-B3)

MS(m/z), 368(M+1); ^1^HNMR (400 MHz, DMSO, after D2O exchange); 1.05(3H,t J = 7.8 Hz), 2.48 (2H, q, J = 7.8Hz), 2.90(2H, m),2.95–3.22 (10H, m), 3.48 (4H,m), 4.01(1H, m), 6.41(1H, s), 6.55–6.65(2H,m).

#### (S)-N-(2-((3-(5-chloro-2-fluoro-4-hydroxyphenoxy)-2-hydroxypropyl)amino)ethyl)morpholine-4-carboxamide (STD-101-B4)

MS(m/z), 392(M+1); ^1^HNMR (400 MHz, DMSO, after D2O exchange): 3.19(2H, m),3.25–3.36(6H, m), 3.48 (2H,m), 3.64(4H, m), 3.99 (2H, m) 4.03(1H, m), 6.72(1H, d, J = 12.2Hz), 7.14(1H,d, J = 8.72Hz).

#### (S)-N-(2-((3-(2,5-difluoro-4-hydroxyphenoxy)-2-hydroxypropyl)amino)ethyl)morpholine-4-carboxamide hemifumarate (STD-101-B5)

MS(m/z), 376(M+1); ^1^HNMR (400 MHz, DMSO, after D2O exchange): 2.85–3.10 (4H, m),3.23 (4H, m), 3.48 (2H,m), 3.54–3.70 (6H, m), 3.91(2H, m) 4.13(1H, m), 6.82(1H, d, J = 8Hz), 7.10(1H,d, J = 12Hz).

#### (S)-N-(2-((3-(3-cyano-4-hydroxyphenoxy)-2-hydroxypropyl)amino)ethyl)morpholine-4-carboxamide (STD-101-B6)

MS(m/z), 365(M+1); ^1^HNMR (400 MHz, DMSO, after D2O exchange): 2.48–2.54 (4H, m), 3.07(2H, m), 3.22 (4H, m), 3.50 (4H,m), 3.81 (2H, m), 3.88 (1H, m), 6.89(1H, d, J = 8.8 Hz), 7.08(1H, d J = 8.8Hz), 7.13(1H,s).

#### (S)-N-(2-((3-(3,5-difluoro-4-hydroxyphenoxy)-2-hydroxypropyl)amino)ethyl)morpholine-4-carboxamide hemifumarate (STD-101-B7)

MS(m/z), 376(M+1); ^1^HNMR (400 MHz, DMSO, after D2O exchange: 2.72–2.86(4H, m), 3.13(4H, m), 3.49 (4H, m), 3.50 (4H,m), 3.60 (2H, m), 3.75 (2H, m), 3.85 (1H,m), 6.68(2H, d, J = 7.2 Hz).

#### (S)-N-(2-((3-(5-cyano-2-fluoro-4-hydroxyphenoxy)-2-hydroxypropyl)amino)ethyl)morpholine-4-carboxamide (STD-101-B8)

MS(m/z), 383(M+1); ^1^HNMR (400 MHz, DMSO, after D2O exchange): 2.85–3.15(4H, m), 3.23–3.34 (4H, m), 3.46(6H, m), 3.98 (2H,m), 4.12(1H, m), 6.86(1H, d, J = 13 Hz), 7.45 (1H, d, J = 9Hz).

#### (S)-N-(2-((3-(4-fluoro-3-(hydroxymethyl)phenoxy)-2-hydroxypropyl)amino)ethyl)morpholine-4-carboxamide hemifumarate (STD-101-B9)

MS(m/z), 372(M+1); ^1^HNMR (400 MHz, DMSO, after D2O exchange: 2.98 (4H, m), 3.12(4H, m), 3.30 (2H, m), 3.51 (4H, m), 3.89(2H, m), 4.12(1H,m), 4.47(2H,s), 6.80 (1H,m), 7.02 (2H,m).

#### (S)-4-(2-hydroxy-3-((2-(2-methoxyphenoxy)ethyl)amino)propoxy)phenol hemifumarate (STD-101-D1)

MS(m/z), 334(M+1); ^1^HNMR (400 MHz, DMSO, after D2O exchange): 2.81 (2H, m), 2.95 (2H, m), 3.07 (2H, t, J = 4Hz), 3.71 (3H, s), 3.96 (1H, m), 4.08 (2H, t, J = 4Hz), 6.64 (2H, d, J = 8,4Hz); 6.73 (2H, d, J = 8Hz), 6.94 (4H, m)

#### (S)-4-(3-((2-(3,4-dimethoxyphenethoxy)ethyl)amino)-2-hydroxypropoxy)phenol hemifumarate (STD-101-D2)

S(m/z), 392(M+1); ^1^HNMR (400 MHz, DMSO, after D2O exchange): 2.70 (2H, m), 2.96 (2H, m), 3.63 (3H, s), 3.66 (3H, s), 3.75 (2H, m), 3.97 (3H, m), 6.64 (2H, d, J = 8,4Hz); 6.73 (2H, d, J = 8Hz), 6.63–6.79 (7H, m).

#### (S)-4-((2-hydroxy-3-(4-hydroxyphenoxy)propyl)amino)-1-morpholinobutan-1-one hemifumarate (STD-101-D3)

MS(m/z), 339(M+1); ^1^HNMR (400 MHz, DMSO, after D2O exchange): 1.73 (2H, m), 2.34 (2H, t, J = 7Hz), 2.75 (2H, m), 2.88 (1H, m), 3.39 (4H, m), 3.55 (4H, m), 3.80 (2H, m), 3.97 (1H, m), 6.65 (2H, d, J = 8Hz), 6.75 (2H, d, J = 8Hz).

#### (S)-3-((2-hydroxy-3-(4-hydroxyphenoxy)propyl)amino)-1-morpholinopropan-1-one hemifumarate (STD-101-D4)

MS(m/z), 325(M+1); ^1^HNMR (400 MHz, DMSO, after D2O exchange): 2.71 (2H, m), 2.88 (1H, m), 3.08 (3H, m), 3.40 (4H, m), 3.60 (4H, m), 3.78 (2H, m), 4.08 (1H, m), 6.66 (2H, d, J = 8Hz), 6.75 (2H, d, J = 8Hz).

#### (S)-3-hydroxy-N-(2-((2-hydroxy-3-(4-hydroxyphenoxy)propyl)amino)ethyl)azetidine-1-carboxamide hemifumarate (STD-101-D5)

MS(m/z), 326(M+1); ^1^HNMR (400 MHz, DMSO, after D2O exchange): 2.98 (4H, m), 3.20 (2H, m), 3.54 (2H, m), 3.81 (2H, m), 3.93 (2H, m), 4.10 (1H, m), 4.37 (1H, m), 6.66 (2H, d, J = 8Hz), 6.75 (2H, d, J = 8Hz).

#### (S)-N-(2-((2-hydroxy-3-(4-hydroxyphenoxy)propyl)amino)ethyl)-3-(hydroxymethyl)azetidine-1-carboxamide hemifumarate (STD-101-D6)

MS(m/z), 340(M+1); ^1^HNMR (400 MHz, DMSO, after D2O exchange): 2.57 (1H,m), 3.00 (3H, m), 3.18 (1H, m), 3.31 (2H, m), 3.50 (4H, m), 3.80 (4H, m), 4.12 (1H, m), 6.66 (2H, d, J = 8Hz), 6.75 (2H, d, J = 8Hz).

#### (S)-N-(2-((2-hydroxy-3-((5-hydroxynaphthalen-1-yl)oxy)propyl)amino)ethyl)morpholine-4-carboxamide (STD-101-E)

MS(m/z), 390(M+1); ^1^HNMR (400 MHz, DMSO, after D2O exchange): 2.77–2.84 (4H, m), 3.21 (6H, m), 3.47 (4H, m), 4.08 (3H, m), 6.87(2H, m), 7.23 (2H, m), 7.65 (2H, d, J = 8Hz).

### cAMP assay

Pharmacological effects of compounds at the cAMP pathway mediated by ADRB1 were evaluated by measuring cAMP production using the homogenous time-resolved fluorescence detection method with HEK-293 cells stably expressing human recombinant ADRB1. Briefly, cells were suspended in HBSS buffer completed with 20 mM HEPES (pH 7.4) and 500 μM IBMX (3-isobutyl-1-methylxanthine) and distributed at a density of 3x10^3^ cells/well. Subsequently, cells were incubated with HBSS (basal control), the full agonist isoproterenol hydrochloride (I5627; Sigma-Aldrich Corp., MO), xamoterol (Santai Labs; http://www.santailabs.com/index.asp), or test compounds for 30 min. Following incubation, the cells were lysed and the fluorescence acceptor (D2-labeled cAMP) and fluorescence donor (anti-cAMP antibody labeled with europium cryptate) were added. After a 60 min incubation with the fluorescence donor and acceptor at room temperature, the fluorescence transfer was measured at 337 nm (excitation) and 620 and 665 nm (emission) using a microplate reader. The cAMP concentration was determined by dividing the signal measured at 665 nm by that measured at 620 nm (ratio). The results were expressed as a percent of the maximum efficacy achieved with isoproterenol. Pharmacological effects of compounds at the cAMP pathway mediated by ADRB2 and ADRB3 were measured as described above using CHO cells stably expressing human recombinant ADRB2 and human SK-N-MC neurotumor cells endogenously expressing ADRB3, respectively.

### β-arrestin assay

Pharmacological activity of compounds in the β-arrestin pathway mediated by ADRB1 were evaluated using an enzyme fragment complementation method with a β‐galactosidase functional reporter. An engineered CHO-K1-ADRB1 PathHunter cell line (DiscoveRx) was used in the assay. In this cell line, the enzyme acceptor (β‐galactosidase fragment) is fused to β-arrestin and the enzyme donor (β‐galactosidase fragment) is fused to the ADRB1. Thus, activation of the ADRB1 stimulates binding of β-arrestin to the ProLink-tagged ADRB1 and forces complementation of the two enzyme fragments, resulting in the formation of an active β‐galactosidase enzyme. Briefly, CHO-K1-ADRB1 PathHunter cell lines were plated in a total volume of 20 μL cell plating reagent (DiscoveRx, 93-0563R0A) at a density of 2,500 cells/well into 384 well microplates and incubated overnight at 37°C in 5% CO_2_. The following day, 5 μL of the full agonist isoproterenol, xamoterol (S), or test compounds was added to cells and incubated at 37°C for 90 min. After the 90 min incubation, 15 μL of PathHunter Detection reagent cocktail (DiscoveRx, 93–0001) was added, followed by a 60 min incubation at room temperature. Chemiluminescent signal was then read with a PerkinElmer Envision (Perkin Elmer, Inc., MA) instrument. The results were expressed as a percent of the maximum efficacy achieved with isoproterenol.

### Crystal structure modeling

Ligands were docked to the binding pocket of PDB: 2YCZ, constrained to a 25 Angstrom box size surrounding the orthosteric pocket. Docking was performed using Schrödinger Glide, with SP precision and after using LigPrep to prepare all ligands with a maximum of 32 stereoisomers and 6 low energy ring conformations. The docking and analysis were facilitated by the open source software: (https://github.com/evanfeinberg/conformation/blob/master/grids.py)).

### *In vitro* primary microglia tumor necrosis factor α (TNFα) assay

Mixed glial cells were obtained from the cerebral cortex of Sprague Dawley rat pups at postnatal days 1–3. Briefly, neonates were euthanized by decapitation and their brain tissues were collected for cortex isolation. The isolated cortex was then trypsinized, triturated, and placed into tissue culture flasks in DMEM supplemented with 10% fetal bovine serum and 1% Penicillin/Streptomycin. After 10 days *in vitro*, microglia were harvested by gentle shaking of the growth flask, plated in a 96 well plate at a density of 30,000 cells/well, and incubated at 37°C overnight. The next day, microglia were stimulated with lipopolysaccharides (LPS) (10 ng/ml) along with isoproterenol, xamoterol (S), or STD-101-D1 at the concentration of 10 μM for 4 hrs at 37°C. Following the 4 hr incubation, cell media was collected and the concentration of TNFα was measured by ELISA (Invitrogen, KRC3011) according to the manufacturer’s instruction.

### Microsomal stability assay

The test compound was pre-incubated with pooled mouse, rat, or human microsomes in 100 mM potassium phosphate buffer (pH 7.4) containing 10 mM MgCl_2_ for 5 min in a 37°C shaking waterbath. After the preincubation, the reaction was initiated by adding freshly prepared NADPH to a final concentration of 1mM. Aliquots of the reaction samples were collected at 0 min, 15 min, 30 min, 45 min, and 60 min after the initiation of the reaction, and quenched with equal volume of acetonitrile. Samples were then mixed and centrifuged, and supernatants were diluted with equal volume of water and used for LC-MS/MS analysis to determine the concentrations of STD-101-D1. Analyte peak areas at different time points were recorded, and the compound remaining was calculated by comparing the peak area at each time point to time zero. The half-life was calculated from the slope of the initial linear range of the logarithmic curve of compound remaining (%) vs. time, assuming first order kinetics.

### Animals

For the *in vivo* LPS study, a total of 45 male C57Bl/6J mice at the age of 10–12 weeks (Jackson Laboratory, Bar Harbor, ME, USA) were used. For the pharmacokinetic study, a total of 36 Sprague-Dawley male rats (Charles River, Wilmington, MA, USA) weighing 280–380 g were used. All animals were kept under a reverse light-dark cycle with lights off at 8:30 AM and on at 8:30 PM in a temperature- and humidity-controlled environment and given food and water *ad libitum*. All animal experiments were conducted in accordance with the U.S. National Institutes of Health Guide for the Care and Use of Laboratory Animals (NIH Publications No. 80–23). All experimental protocols were reviewed and approved by the Stanford University Institutional Animal Care and Use Committee (IACUC) (Protocol #—18466).

### *In vivo* LPS assay

C57Bl/6J mice (10–12 weeks old) were injected with xamoterol (3 mg/kg; subcutaneous; n = 11), STD-101-D1 (3 mg/kg; intraperitoneal; n = 4) or vehicle (5% DMSO/20%PEG400/water; intraperitoneal; n = 12) 15 min prior to LPS (50 ug/kg; intraperitoneal). A control group was injected with vehicle 15 minutes prior to saline (n = 18). Following injections, mice were single-housed. At 90 min post-LPS/saline, mice were deeply anesthetized with isoflurane and blood was collected from the right ventricle via cardiac puncture (23 g needle) into lithium heparin-containing vials (BD microtainer plasma tubes). Subsequently, brains were collected after perfusion with phosphate-buffered saline. Plasma was separated by centrifugation (11,000 rpm for 3 minutes) within 60 min of collection and stored at -80°C until analysis. The concentration of TNFα in the collected plasma was measured by ELISA (Invitrogen, KMC3012) according to the manufacturer’s instruction. The LPS-induced inflammatory response in the CNS was assessed by measuring mRNA expression for genes related to neuroimmune activation in brain tissue according to the previously reported method [11]. Briefly, total RNA was extracted from hippocampus containing coronal sections using the RNeasy Lipid Tissue Mini Kit (Qiagen), and transcribed into cDNA (Superscript III, Invitrogen). PCR was performed in duplicate using TaqMan gene expression assays, TNFα (Mm00443258_m1), IL1b (Mm00434228_m1), IL6 (Mm00446190_m1), and glyceraldehyde-3-phosphate dehydrogenase (GAPDH; Mm99999915_g1). Fold changes of expression relative to control were determined after normalization to GAPDH. Relative quantification and fold change were calculated by the comparative CT method [19]. Brain uptake of STD-101-D1 was determined in brain tissue. Brain tissues were homogenized in distilled water at a ratio of 1:3 (weight of tissue:volume of water) and homogenates were analyzed using LC-MS/MS.

### Pharmacokinetic studies

Two cohorts of Sprague-Dawley rats were used in two independent studies. All animals were fasted overnight before the experiment with free access to water. In study 1, a total of 18 rats were used for a 4 hr time course pharmacokinetic (PK) study. Rats were anesthetized with 3% isoflurane and catheters were implanted into the jugular and/or portal veins for compound administration and/or blood sampling at 1 to 2 days prior to the experiments as previously described [20, 21]. On the day of the experiment, xamoterol was freshly prepared in saline. STD-101-D1 was freshly prepared in 5% DMSO, 20% PEG, and 75% distilled water. The prepared xamoterol or STD-101-D1 was administered to the cannulated rats at a dose of 10 mg/kg intravenously (IV), intraperitoneally (IP), or orally (PO) (n = 3 per route). For IV and IP groups, approximately 150 μL aliquot of blood samples were collected via jugular vein catheters before drug administration, and at 1, 5, 10, 45, 60, 90, 120, and 180 min after drug administration. For the PO group, approximately 150 μL aliquot of blood samples were collected via jugular and portal vein catheters before drug administration, and at 1, 5, 10, 45, 60, 90, 120, and 180 min after drug administration. Four hours post-dose, rats were deeply anesthetized with isoflurane and blood samples were collected by cardiac puncture. In study 2, a total of 18 rats were used for a 20 min post-dose collection study. Xamoterol and STD-101-D1 were freshly prepared as described above and administered to rats at a dose of 10 mg/kg via IV, IP, or PO routes (n = 3 per route). At 20 min post-dose, rats were deeply anesthetized with isoflurane and blood samples were collected by cardiac puncture. Brains were collected after perfusion with phosphate buffered saline. All plasma samples were immediately separated after collection by centrifugation (11,000 rpm for 3 minutes) and stored at −80°C until analysis. The brain tissue samples were homogenized in distilled water at a ratio of 1:3 (weight of tissue:volume of water), and the homogenates were stored at −80°C until analysis. The concentrations of xamoterol and STD-101-D1 in plasma and brains were determined using LC-MS/MS (AB SCIEX QTRAP 4000 mass spectrometer coupled to a Shimadzu UFLC system). For xamoterol, LC separation was carried out on an Agilent Zorbax SB-Phenyl column (5 μm, 2.1×50 mm) with isocratic elution using a mobile phase composed of 30% methanol and 70% water with 0.1% of formic acid. Tulobuterol was used as the internal standard. The flow rate was set to 0.45 ml/min. Column temperature was 25°C. The analysis time was 2.2 min. The injection volume was 20 μl. The mass spectrometer was operated in the positive mode with multiple-reaction monitoring (MRM). The m/z 340.2→253.2 and 228.1→154.1 transitions were used for Xamoterol and the internal standard, respectively. For STD-101-D1, LC separation was carried out on a Phenomenex Synergi Polar-RP column (2.5 μm, 2 mm × 50 mm) with a flow rate of 0.25 ml/min at room temperature. Mobile phase A consisted of 10 mM ammonium acetate and 0.1% formic acid in LCMS grade water. Mobile phase B consisted of 10 mM ammonium acetate and 0.1% formic acid in LCMS grade acetonitrile:water 90:10% (v/v). The HPLC elution program was as follows: 35% B (0.3 min)→85% B (linear increase in 1.2 min)→35% B (linear decrease in 0.1 min)→35% B (0.9 min). Five μl of the extracted samples were injected. The mass spectrometer was operated in the positive mode with multiple-reaction monitoring (MRM) with the transition m/z 334.1→210.1 and 334.1→100.2. Data acquisition and analysis were performed using the Analyst 1.6.1 software (AB SCIEX, CA).

### Cardiovascular studies

Effects of compounds on heart rate and blood pressure were measured in male Sprague-Dawley rats using a fluid filled catheter-transducer system with a disposable blood pressure transducer MLT 0699 connected to a PowerLab 8/30 recording unit with Quad Bridge Amp (AD instruments, CO). Briefly, rats were anesthetized with isoflurane (3–4% for induction and 1.5% for maintenance) and a 1 cm longitudinal incision was made on the ventral aspect of the tail exposing the tail artery. A polyethylene catheter (PE50) filled with heparinized saline was then inserted into the tail artery and connected to the blood pressure transducer system. After ensuring the absence of any air bubbles, 5–10 min of baseline systolic and diastolic blood pressure along with heart rate measurements were recorded using LabChart Pro (AD Instruments, CO). After establishing the baseline, xamoterol or STD-101-D1 was subcutaneously administered and changes in blood pressure and heart rate measurements were recorded for an additional 30 min. The effects of xamoterol or STD-101-D1 on heart rate were calculated as the difference between average values recorded during the baseline recording and average values recorded during 5 to 10 min after the administration of compound. The effects of xamoterol or STD-101-D1 on blood pressure were calculated as the difference between average values measured during the baseline recording and the lowest value measured during the 10 min period after administration of the compound.

### Calculation and statistics

*In vitro* pharmacology data for the cAMP pathway represent 2–5 experiments performed singly or in duplicate. *In vitro* pharmacology data for the β-arrestin pathway represent technical replicates within a single experiment. Curve fitting was performed with GraphPad Prism 5.0 software (GraphPad Software, CA) using the equation for a single-site sigmoidal, dose-response curve with a variable slope. EC_50_ values were expressed as geometric means (95% confidence limits). Statistical analyses were performed with GraphPad Prism 5.0. One-way analyses of variance, followed by Dunnett’s test for post-hoc analyses were performed on *in vitro* and *in vivo* TNFα data. Pharmacokinetic analyses were performed using the Phoenix WinNonlin Professional Edition computer software version 2.0 (Certara, NJ). Differences were considered to be significant at a level of *p* < 0.05. In all cases, outliers were excluded according to Grubbs’ test and p < 0.05 was considered to be significant.

## Results

### Structure-activity relationship of compounds at the cAMP pathway mediated by ADRB1

The β-aminoalcohol linking moiety is a crucial pharmacophoric element of most ADRB1 binding compounds, including xamoterol [22]. Consequently, structure-activity relationship (SAR) studies and drug discovery programs have focused upon making structural changes at the two terminal sites of ADRB1 binding molecules. We hypothesize that affinity and selectivity at ADRB1 can be significantly improved and manipulated by making the appropriate structural changes to xamoterol at the phenolic and morpholino subsites (Fig 1). As such, we prepared 18 analogs of xamoterol with the objective of (1) maintaining affinity and efficacy for ADRB1, and (2) increasing brain penetration. Pharmacological effects of the analogs on ADRB1 were then evaluated by measuring cAMP production.

**Fig 7:**
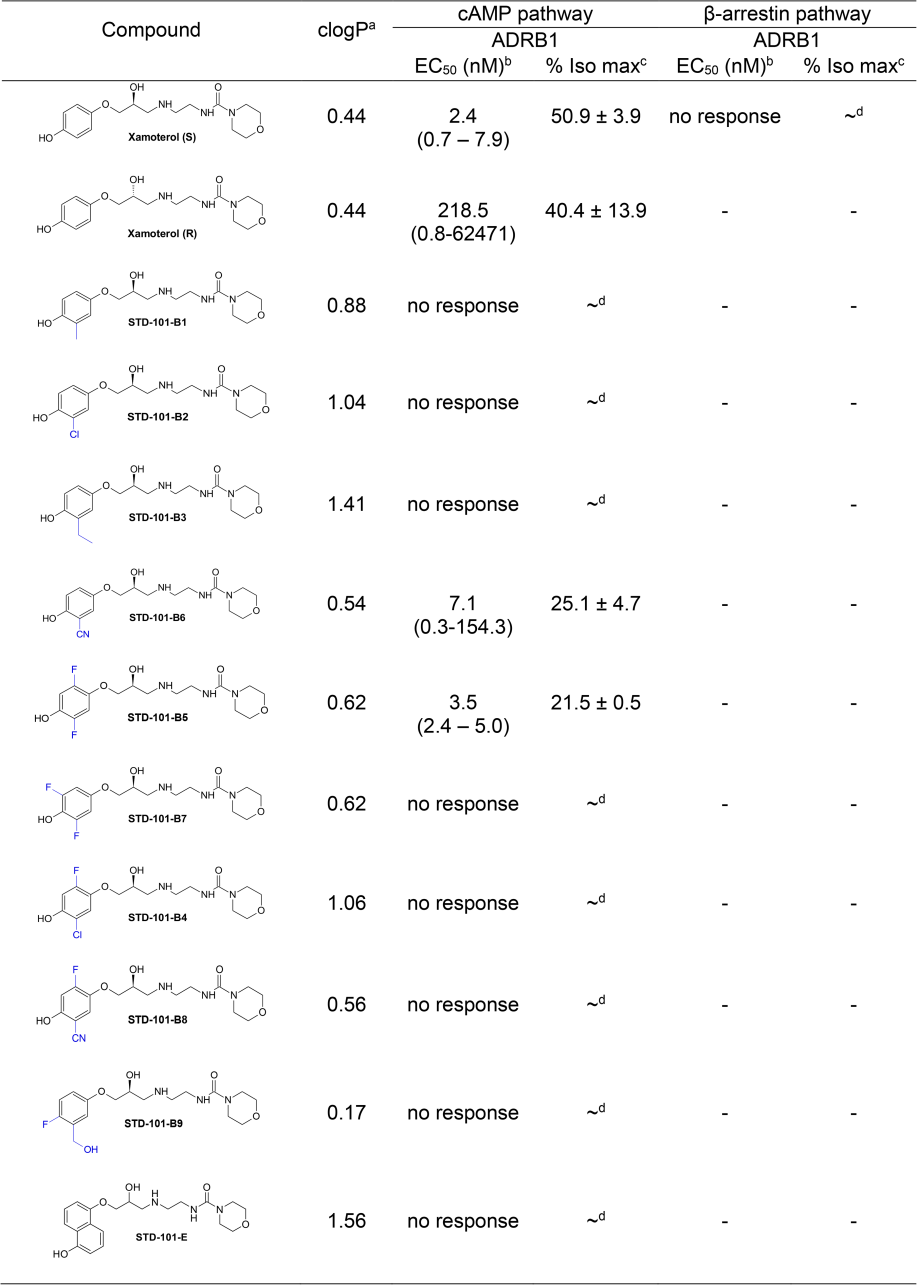
Effects of structural modifications of the phenolic OH moiety of xamoterol on the cAMP pathway mediated by ADRB1. clogP^a^, Calculated with ChemDraw Pro Version 16.0 (PerkinElmer Health Sciences, CT; EC_50_ (nM)^b^, Geometrical mean of EC50 values from at least two independent experiments; % Iso max^c^, Percent efficacy compared to the maximum response achieved with isoproterenol; ~^d^, Could not be determined.

**Fig 8:**
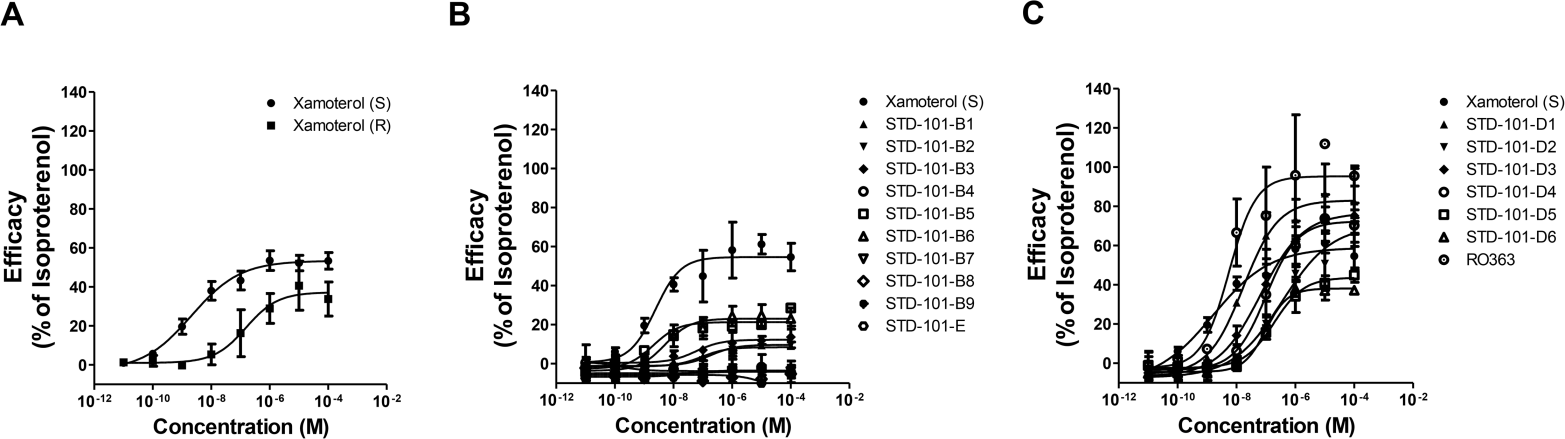
Concentration-response effects of compounds on the cAMP pathway via ADRB1. Data are expressed as a percentage of maximum efficacy obtained with the full agonist isoproterenol. Values represents means ± S.E.M.s (1–2 experiments with n = 1–2).

The major shortcomings of xamoterol for CNS indications are its poor oral bioavailability and rapid clearance [18]. Most of the absorbed drug in humans is excreted in the urine unchanged, with a substantial amount also excreted as the sulfate after first pass by the liver. We therefore envisioned that structural modifications of the phenol group could enhance PK properties of the molecules. In the first series of compounds, the phenol group in xamoterol is substituted with a variety of electron donating and electron withdrawing groups (Fig 7). These diverse structural modifications also alter the lipophilicity of the compounds and modulate other molecular properties, such as tPSA, which should enhance oral absorption. As the (S)-enantiomer of xamoterol was more potent and efficacious than the (R)-enantiomer, all structural modifications were made on the (S)-enantiomer (Fig 7 and Fig 8A). As demonstrated by the data shown in Figs 7 and 8B, the introduction of methyl, chlorine, and ethyl residues (STD-101-B1, B2, and B3) to sterically crowd the phenolic OH abolished the ADRB1 agonist activity. These analogs produced no pharmacological effects up to concentrations of 100 μM (Fig 7 and Fig 8B). On the other hand, introduction of cyanide to this position (STD-101-B6) led to a decrease in both potency and efficacy compared to xamoterol (S) (Fig 7 and Fig 8B). The addition of fluorines at C2 and C5 with respect to phenolic OH (STD-101-B5) resulted in an approximately 2-fold efficacy decrease. A fluorine substitution at C3 and C5 (STD-101-B7) abolished the agonist activity completely (Fig 7 and Fig 8B) as did substitution of the larger substituents, chlorine or cyano, ortho to the phenolic OH group (STD-101-B4, STD-101-B8). Compounds (STD-101-B9) and STD-101-E (Fig 7 and Fig 8B) were also inactive.

**Fig 9:**
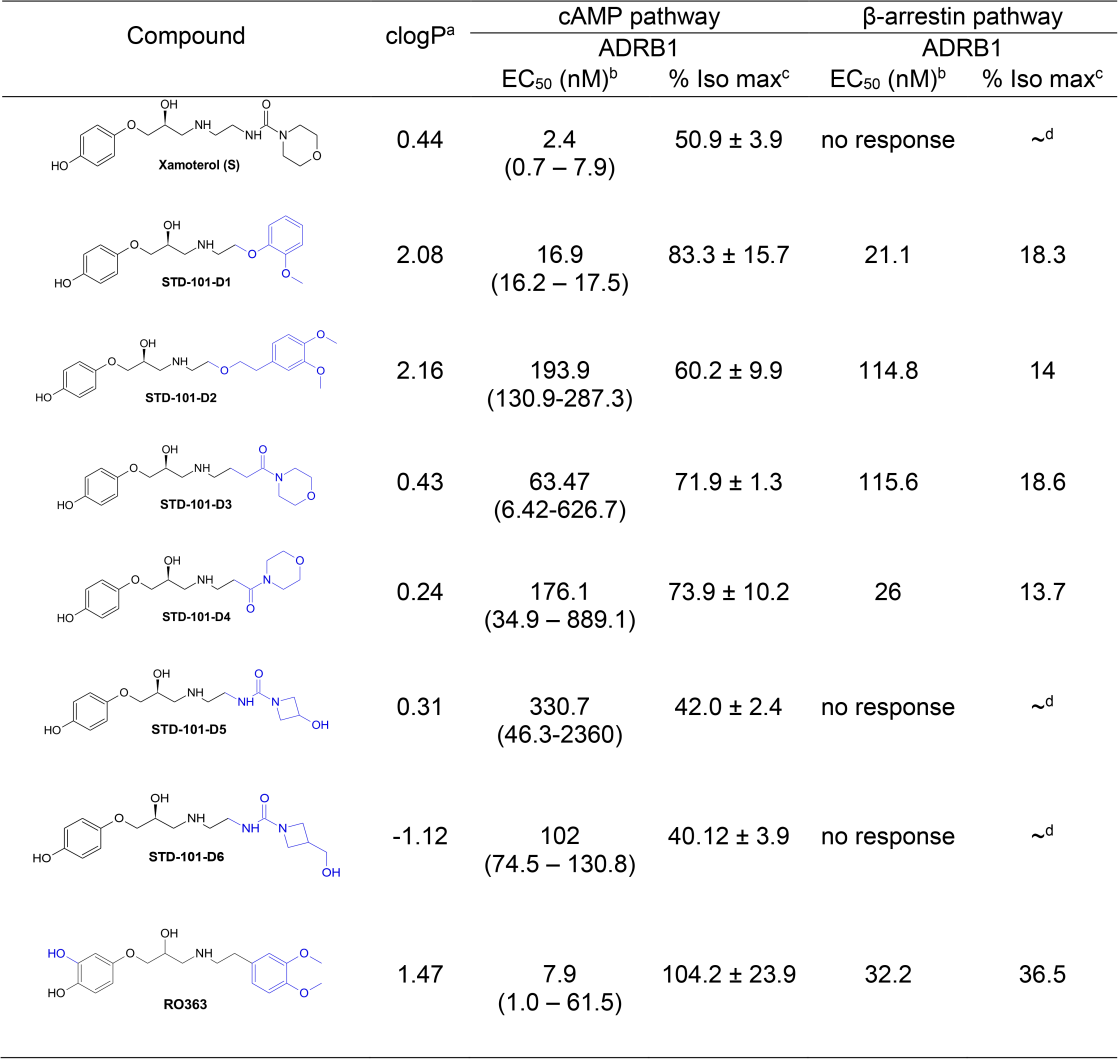
Effects of structural modifications of the morpholino urea moiety of xamoterol on the cAMP pathway mediated by ADRB1. clogP^a^, Calculated with ChemDraw Pro Version 16.0; EC_50_ (nM)^b^, Geometrical mean of EC50 values from at least two independent experiments; % Iso max^c^, Percent efficacy compared to the maximum response achieved with isoproterenol; ~^d^, Could not be determined.

In the second series of compounds, analogs incorporated structural modifications of the morpholino urea moiety of xamoterol (Fig 9). Replacement of morpholine-4-carboxamide moiety in xamoterol (S) by (2-methoxyphenoxy)ethanamine (STD-101-D1) led to an approximately 7-fold decrease in potency and approximately 30% increase in efficacy (Fig 8C and Fig 9). Substitution with [2-(3,4-dimethoxyphenyl)ethoxy]ethanamie (STD-101-D2) resulted in an approximately 80-fold decrease in potency and an approximately 10% increase in efficacy. Similarly, analogs where the NH of the urea was replaced with methylene (STD-101-D3) or completely excised (STD-101-D4) led to approximately 26- and 73-fold decreases in potency, respectively. However, these modifications in STD-101-D3 and STD-101-D4 led to approximately 20% increases in efficacy (Fig 8C and Fig 9). Substitution of the morpholine group in xamoterol (S) with smaller ring systems, 3-hydroxyazetidine (STD-101-D5) and 3-hydroxymethylazetidine (STD-101-D6) led to more than approximately 40-fold decreases in potency. Interestingly, these changes in STD-101-D5 and STD-101-D6 only marginally affected their efficacies. Substitution of the morpholine group in xamoterol (S) with 1,2-dimethoxybenzene (RO363) led to an approximately 3-fold decrease in potency and an approximately 2-fold increase in efficacy (Fig 8C and Fig 9). Importantly, none of the second series of compounds having partial agonist activity on the cAMP pathway via ADRB1 show activity on cAMP pathways via ADRB2 or ADRB3, suggesting that they are partial agonists selective for ADRB1 versus ADRB2 and ADRB3 (data not shown).

### Signaling bias of the key compound

**Fig 10:**
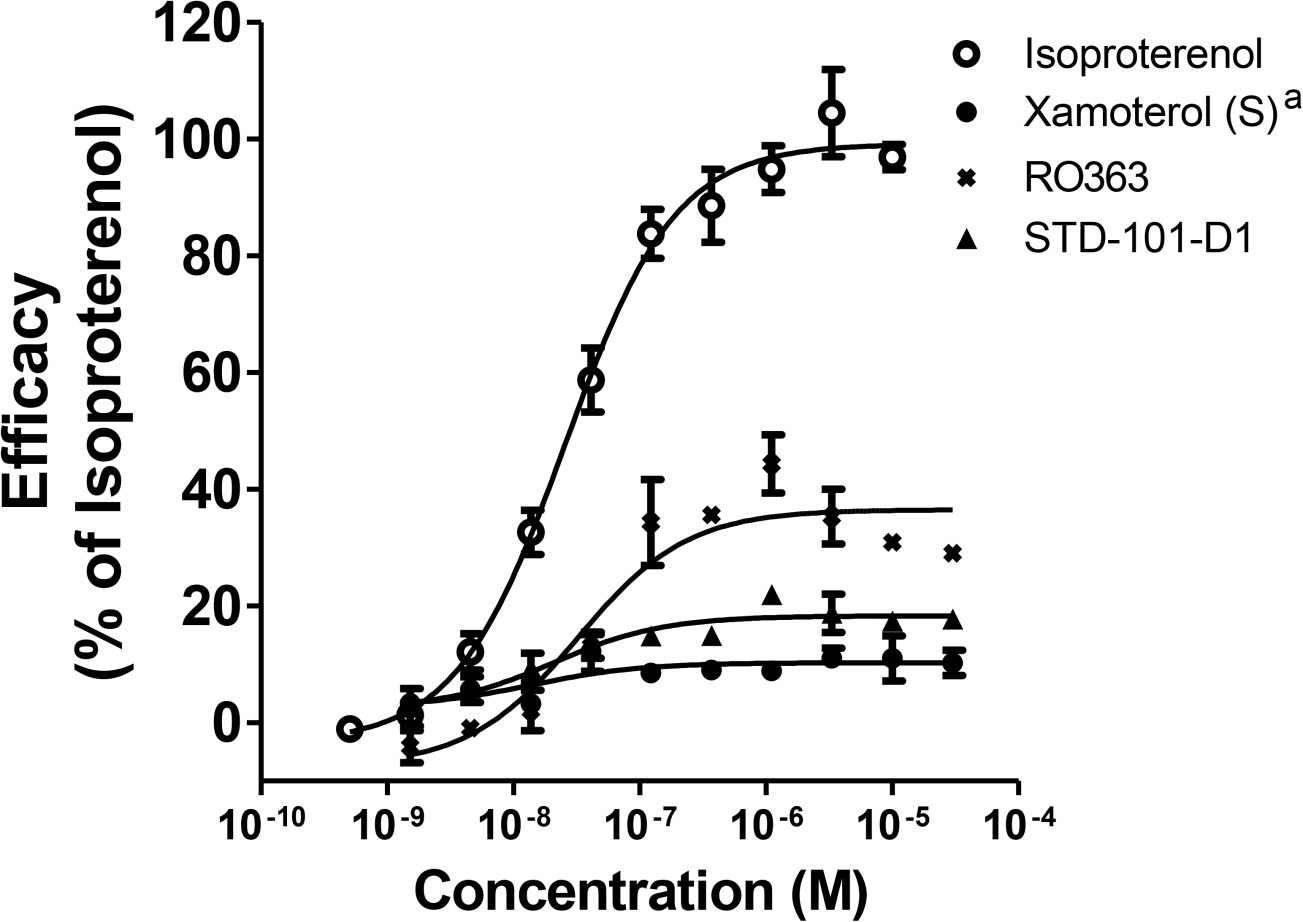
Concentration-response effects of compounds on the β-arrestin pathway via ADRB1. Data are expressed as a percentage of maximum efficacy obtained with the full agonist isoproterenol. Values represents means ± S.E.M.s (1–2 experiments with n = 1–2). Xamoterol^a^; data has been previously published [11].

To identify G protein-biased agonists of ADRB1, we assessed the pharmacological activity of the compounds that showed significant agonistic activity at the cAMP pathway (e.g. STD-101-D1 through STD-101-D6 and RO363) on the ADRB1-mediated β-arrestin pathway. As a classical unbiased agonist, isoproterenol produced concentration-dependent responses at the β-arrestin pathway via ADRB1 with an EC_50_ value of 31.3 nM (Fig 10). In contrast, xamoterol (S) did not produce concentration-dependent responses up to 30 μM, indicating that it has a very high level of functional selectivity toward the cAMP pathway (Fig 10). The partial agonists STD-101-D1 through STD-101-D4 displayed very weak partial agonist activity at the β-arrestin pathway, producing less than 20% efficacy compared to the full agonist isoproterenol (Figs 9 and 10). The other two compounds STD-101-D5 and STD-101-D6 did not produce concentration-dependent responses up to 30 μM (Fig 9). However, RO363, which showed full agonistic activity on the ADRB1-mediated cAMP pathway, produced partial agonistic activity on the ADRB1-mediated β-arrestin pathway, achieving 36.5% efficacy with an EC_50_ value of 32.2 nM. On the basis of potency and partial agonistic activity on the cAMP pathway and functional selectivity for the cAMP pathway over the β-arrestin pathway, the compound STD-101-D1 was selected for further *in vitro* and *in vivo* testing.

### Crystal structure modeling

**Fig 11:**
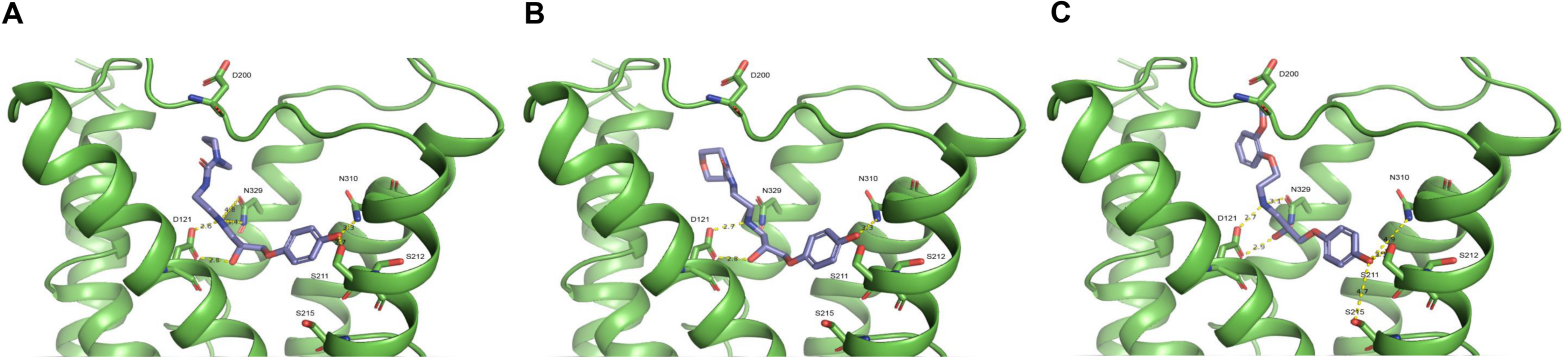
**Crystal structure of ADRB1 with xamoterol (S) (cyan, panel A), xamoterol (R) (blue, panel B), and STD-101-D1 (salmon, panel C) docked into the ligand-binding site.** The transmembrane regions are shown as green ribbons, and. Putative interactions are displayed as yellow dashed lines with estimated distance in angstroms (Å). The carbon-nitrogen chain of xamoterol (S) is predicted to pack approximately 1.0 Angstroms closer to D121 and Transmembrane Helix 3 compared to xamoterol (R), and its morpholino ring rests in a rotated pose as well.

In order to investigate the molecular mechanisms of xamoterol (S), xamoterol (R), and STD-101-D1, we used molecular docking to predict the binding poses of the three ligands (Fig 11). All three compounds are predicted to have a common set of characteristic interactions, including: 1) a salt bridge between the protonated secondary amine of the compound and the carboxylic acid of D121 of the receptor, 2) a hydrogen bond between the beta amino alcohol of the compound and D121 of the receptor, and 3) a hydrogen bond between the phenolic hydroxyl group of the compound and S211 of the receptor. Perceptible differences between the binding poses of the three compounds are qualitatively subtle and, in lieu of a fully active crystal structure of ARB1, are difficult to connect with differences in activity of the compounds. The carbon-nitrogen chain of xamoterol (S) packs much more closely to D121 and to the transmembrane (TM) domain 3 than xamoterol (R), likely owing to the difference in stereochemistry between the two compounds. In addition, whereas the phenolic moiety of all three drugs overlaps nearly exactly in the predicted pose, the six-membered heterocycle of both xamoterol enantiomers and the phenyl group of STD-101-D1 are predicted to adopt different positions in the extracellular portion of the binding pocket. These differences serve as potential mechanisms for the gap in affinity between the two chiral forms of xamoterol.

### Neuroimmuno-modulatory effects of the key compound

**Fig 12:**
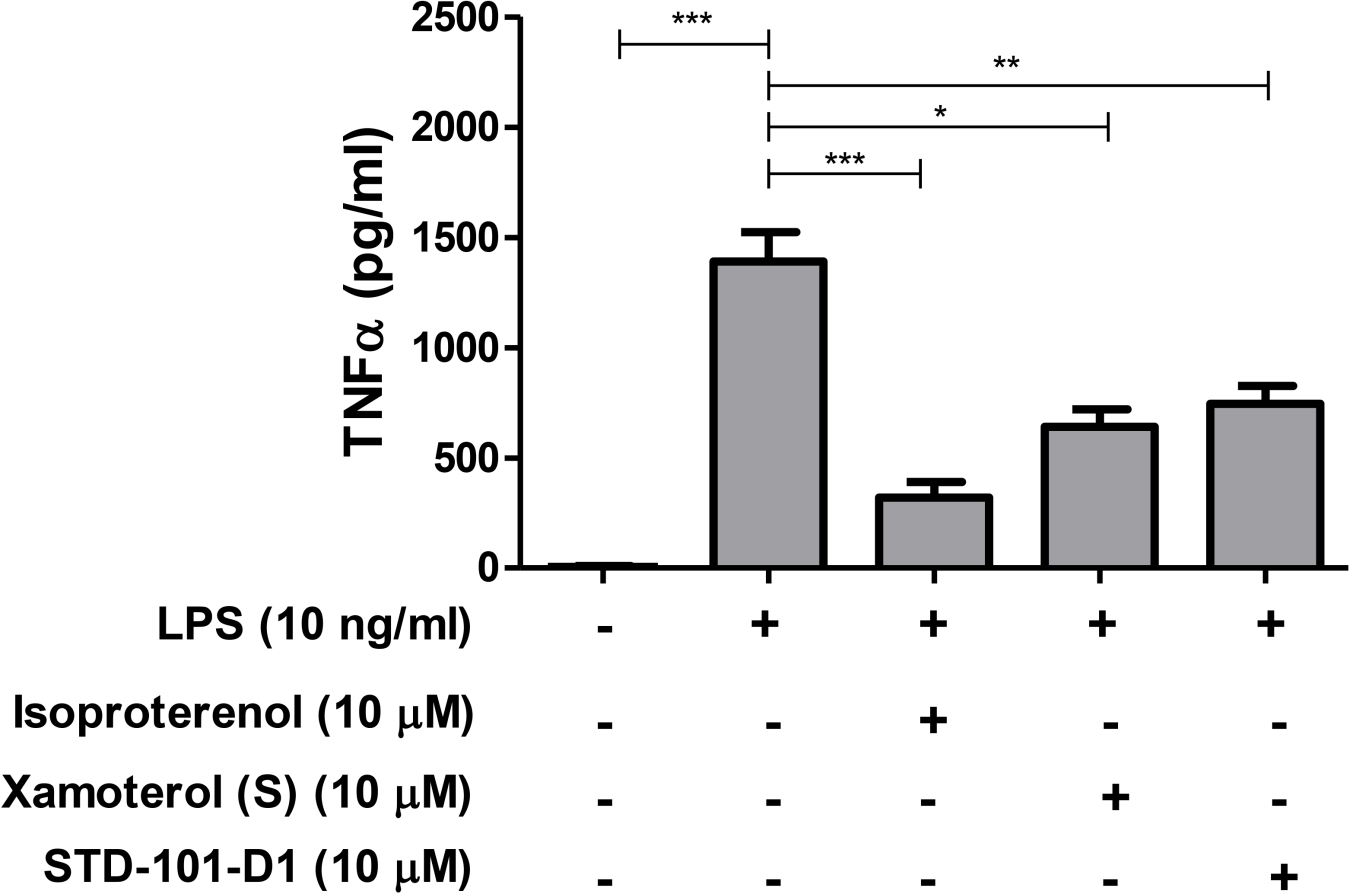
Inhibitory effects of ADRB1 ligands on LPS-induced TNFα response in primary microglia. Data are represented as mean ± S.E.M.s of four independent experiments (n = 3–18 per group, * *p <* 0.05, **p < 0.01, *** *p <* 0.001, one-way ANOVA followed by Dunnett’s multiple comparison against LPS exposure alone).

**Fig 13:**
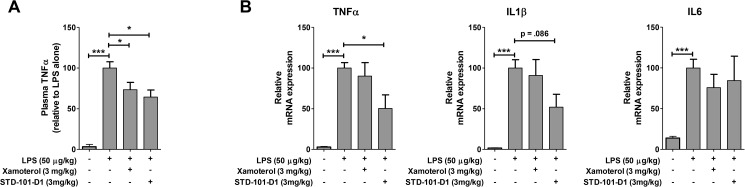
Inhibitory effects of ADRB1 ligands on LPS-induced TNFα response in mice. (A) Plasma TNFα concentrations in control animals and animals pretreated with xamoterol or STD-101-D1 90 min after LPS injection. (B) TNFα, IL1β, and IL6 mRNA expression in homogenized cortical tissue from control mice and animals pretreated with xamoterol or STD-101-D1 90 min after LPS injection. Data are represented as mean ± S.E.M.s of three independent experiments. (n = 4–14 per group, * *p <* 0.05, **p < 0.01, *** *p <* 0.001, one-way ANOVA followed by Dunnett’s multiple comparison).

The TNFα signaling pathway has been strongly implicated in AD pathology and neuroinflammatory diseases [23–25]. With the aim of identifying compounds that have therapeutic potential for AD and neuroinflammatory diseases, we assessed whether our key compound STD-101-D1 could modulate the TNFα signaling pathway. First, the effects of STD-101-D1 on the TNFα signaling pathway were assessed *in vitro* by stimulating primary microglia with the bacterial endotoxin LPS for 4 hrs in the absence or presence of the test compounds. Previous studies with this model have shown that the effects of xamoterol on the LPS-induced TNFα response are dependent on ADRB1; its effects were reversed by the selective ADRB1 antagonists CGP 20712A and betaxolol, but not by the selective ADRB2 antagonist ICI-118551 [11]. As shown in Fig 12, stimulation of primary microglia cells with LPS led to a significant increase in TNFα levels. Treatment with isoproterenol, the unbiased full agonist of ADRB1, inhibited the LPS-induced TNFα production by approximately 80%, whereas treatment with xamoterol (S) inhibited LPS-induced TNFα production by approximately 55%. The key compound STD-101-D1 reduced LPS-induced TNFα production by approximately 50%. In order to investigate whether STD-101-D1 could also inhibit the TNFα response *in vivo*, the effects of STD-101-D1 on mice exposed to LPS were also examined. Administration of LPS resulted in a peripheral inflammatory response as measured by increased levels of TNFα in plasma at 90 min post-LPS (Fig 13A). The LPS-induced systemic TNFα response was markedly inhibited by pre-treating mice with xamoterol (S) or STD-101-D1 at 3 mg/kg (Fig 13A). Administration of LPS also led to an inflammatory response in the CNS at 90 min post-LPS. Brain tissue from LPS-injected mice showed increased gene expression of proinflammatory cytokines TNFα, IL-1β, and IL-6 (Fig 13B). The LPS-induced inflammatory TNFα response in the brain was attenuated by pre-treating mice with STD-101-D1 at 3 mg/kg (Fig 13B). We observed a trend for attenuation of CNS IL-1β with STD-101-D1 and no effect at IL-6. Significant effects of xamoterol were not observed in CNS inflammatory markers at this time point, possibly due to timing of LPS response and differences in PK properties of xamoterol relative to STD-101-D1. The finding that 129.9 ± 28.2 ng/g (n = 5) of STD-101-D1 were detected at 105 min post-dose in brain homogenates from the mice pretreated with STD-101-D1 (3 mg/kg) indicates that it gets to the brain.

### Pharmacokinetic properties of the key compound

**Fig 14:**
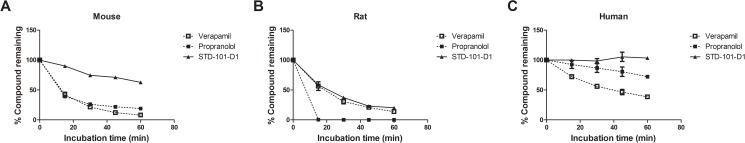
Metabolic stability in mouse, rat, and human microsomes. STD-101-D1 and two reference compounds verapamil and propranolol were incubated at 0.1 uM in mouse (A), rat (B), or human (C) liver microsomes. Serial samples were removed up until 60 min. All experiments were performed in duplicate, and data are represented as mean ± S.E.M.

The findings that STD-101-D1 has potent partial agonistic activity on ADRB1 with functional selectivity for the cAMP pathway and produces anti-inflammatory effects suggested that it could be the promising drug-like lead compound. Toward evaluating this intriguing possibility, we profiled the PK properties of STD-101-D1 in an *in vitro* microsomal stability assay and *in vivo* pharmacokinetic studies. First, the metabolic stability of STD-101-D1 was assessed together with the reference compounds verapamil (a calcium channel blocker) and propranolol (a beta blocker) using microsomes from the mouse, rat, and human. In mouse microsomes, both verapamil and propranolol were readily metabolized, showing half-lives of less than 30 minutes (Fig 14A). In comparison, STD-101-D1 was stable in mouse liver microsomes, with 62.8% of the compound remaining after 60 min (Fig 14A). The rate of disappearance of verapamil, propranolol, and STD-101-D1 was greater in rat microsomes as compared to mouse microsomes (Fig 14B). Verapamil and propranolol were readily metabolized with half-lives of 20.9 min and 7 min, respectively. STD-101-D1 was metabolized with a half-life of 25.2 min in rat microsomes. In human microsomes, verapamil was metabolized with a half-life of 44.1 min; propranolol was stable with 72.2% of compound remaining after 60 min (Fig 14C). STD-101-D1 was very stable in human microsomes, with almost no reduction after 60 min. (Fig 14C).

**Fig 15:**
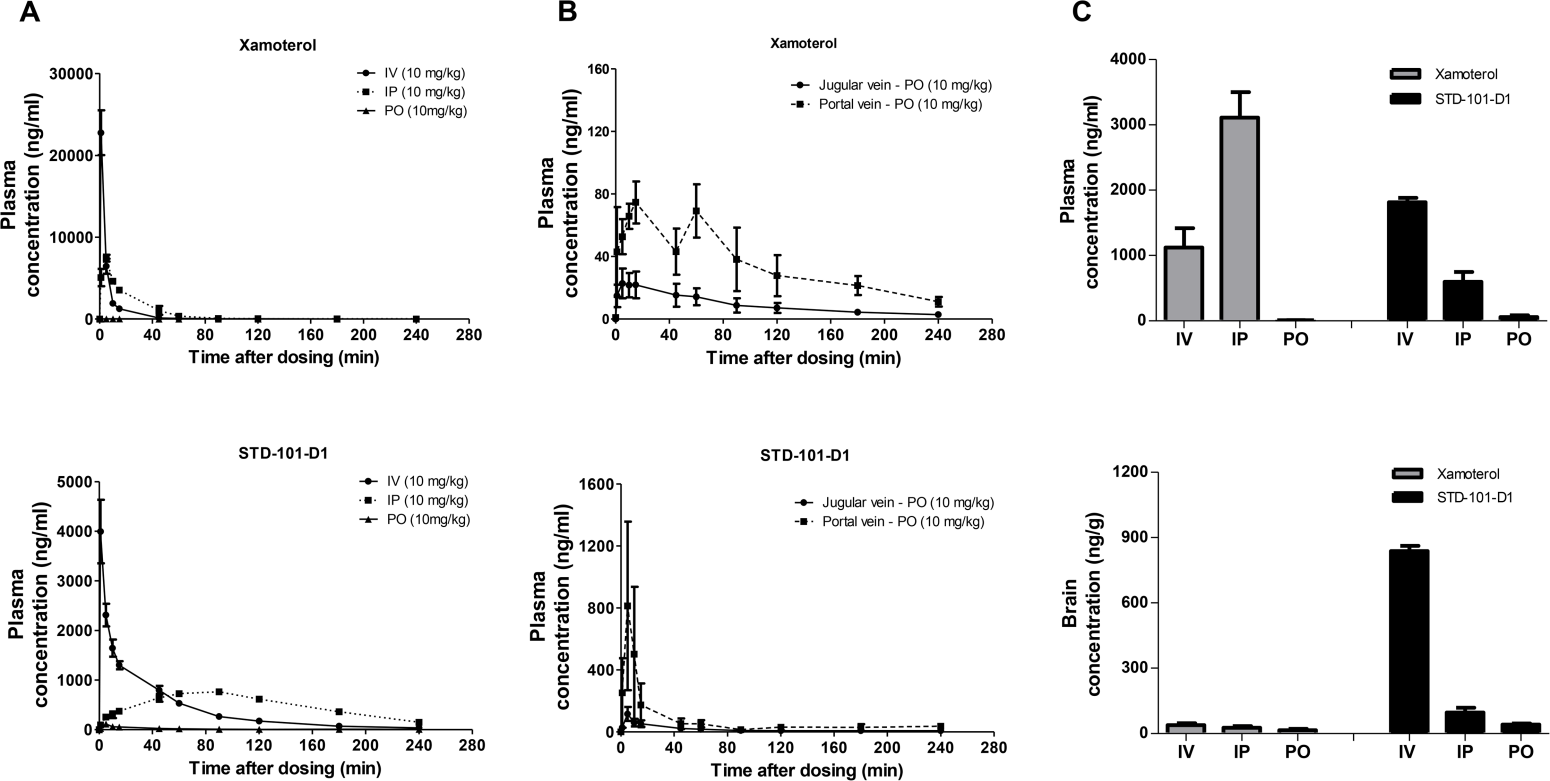
Pharmacokinetics of xamoterol and STD-101-D1. Systemic (A) and portal vein (B) plasma concentrations of xamoterol and STD-101-D1 as a function of time after a single injection of xamoterol (10 mg/kg) or STD-101-D1 (10 mg/kg) via intravenous (IV), intraperitoneal (IP) and oral (PO) administration. Plasma and brain (C) concentrations of xamoterol and STD-101-D1 in rats collected 20 min after a single injection of xamoterol or STD-101-D1 (10 mg/kg) via IV, IP, and PO administration. Data are represented as mean ± SEM (n = 3 per route).

*In vivo* pharmacokinetic properties of STD-101-D1 were also evaluated in male Sprague-Dawley rats in 4-hr time-course PK and 20-min post-dose collection studies after IV, IP, and PO administration of STD-101-D1. In comparison, the pharmacokinetic properties of xamoterol were also determined. The 4-hr time-course PK study revealed that xamoterol was cleared rapidly (Fig 15A, Table 1). Consistent to what has been reported, xamoterol’s oral bioavailability was low (1.7%). Concentrations of xamoterol in the jugular and portal veins were consistently low, indicating that the low absolute oral bioavailability of xamoterol is due to poor absorption. Compared to xamoterol, STD-101-D1 was cleared more slowly and remained in the system for a longer period of time (Fig 15A, Table 1). Following IP injection, STD-101-D1 was rapidly and very significantly absorbed, as evidenced by the systemic plasma concentrations (Fig 15A). The maximum concentration (C_max_) of 762 ng/mL was achieved in systemic plasma after IP administration at 90 min post-dose (Table 1). On the other hand, STD-101-D1 was minimally absorbed after oral administration, resulting in low oral bioavailability (6%) (Fig 15A and 15B). In the systemic (jugular vein) circulation, the C_max_ of STD-101-D1 was 116 ng/ml after oral administration. The corresponding C_max_ of STD-101-D1 in portal veins was 946 ng/ml (Table 1).

**Table 1:** Pharmacokinetic parameters of xamoterol and STD-101-D1 determined in the 4-hr time-course study.

	Route	C_max_ (ng/mL)	AUC_inf_(h*ng/mL)
Xamoterol	IV	22200 ± 4029	2446 ± 601
	IP	7443 ± 730	2834 ± 517
	PO–Systemic	23 ± 16	42.5 ± 29.5
	PO–Portal	79.8 ± 23.8	175.9 ± 53.4
STD-101-D1	IV	3997 ± 1107	1796 ± 256
	IP	762 ± 67	2159 ± 310
	PO–Systemic	116 ± 78	101 ± 29
	PO–Portal	946 ± 867	362 ± 367

The 20 min post-dose collection study revealed that systemic exposure to xamoterol was low after oral administration, in line with its low oral bioavailability. Plasma concentrations of xamoterol at 20 min after PO administration were 0.7% and 0.2% of the plasma concentrations achieved via IV and IP administration, respectively (Fig 15C, Table 2). STD-101-D1 administration led to plasma concentrations comparables with those obtained from xamoterol administration. However, STD-101-D1 had higher CNS penetration, with 22-fold higher brain concentrations after IV treatment of STD-101-D1 compared to xamoterol (Fig 15C, Table 2).

**Table 2:** Pharmacokinetic parameters of xamoterol and STD-101-D1 determined in the 20-min post-dose collection study.

		Plasma concentration (ng/ml)	Brain concentration (ng/g)
Xamoterol	IV	1121 ± 297.1	38.3 ± 9.3
	IP	3113 ± 388.6	26.5 ± 8.4
	PO	8.1 ± 2.0	15.8 ± 5.7
STD-101-D1	IV	1813 ± 67.41	838.7 ± 23.6
	IP	597.7 ± 149.8	96.0 ± 22.6
	PO	56.9 ± 29.2	41.7 ± 5.3

### Pharmacological specificity of the key compound

In light of its promising *in vitro* pharmacology profiles and improved brain penetration, STD-101-D1 was screened against a panel of CNS relevant targets, including G protein-coupled receptors and transporters [26]. In comparison, off-target binding of xamoterol (S) was also determined. Xamoterol (S) displays a distinct preference for ADRB1 and shows low off-target affinity (Ki > 10 μM) for a broad range of neurotransmitter transporters, ion channels and other CNS proteins (including opioid, dopamine, serotonin, nicotinic acetylcholine, muscarinic acetylcholine, and N-methyl-D-aspartate receptors) (S1 Table). In contrast, STD-101-D1 displays a significant affinity for non-ADRB1 binding including 5-HT1A, 5-HT2B, α1A adrenergic, α1D adrenergic, and D3 dopaminergic receptors, while maintaining nominal affinity for several other CNS proteins including opioid, histamine, and muscarinic acetylcholine receptors (S2 Table). The fact that STD-101-D1 binds to non-ADRB1 CNS targets suggests that its neuroimmune-modulatory effects shown in *in vitro* and *in vivo* LPS studies should be interpreted cautiously.

### Cardiovascular effects of the key compound

**Fig 16:**
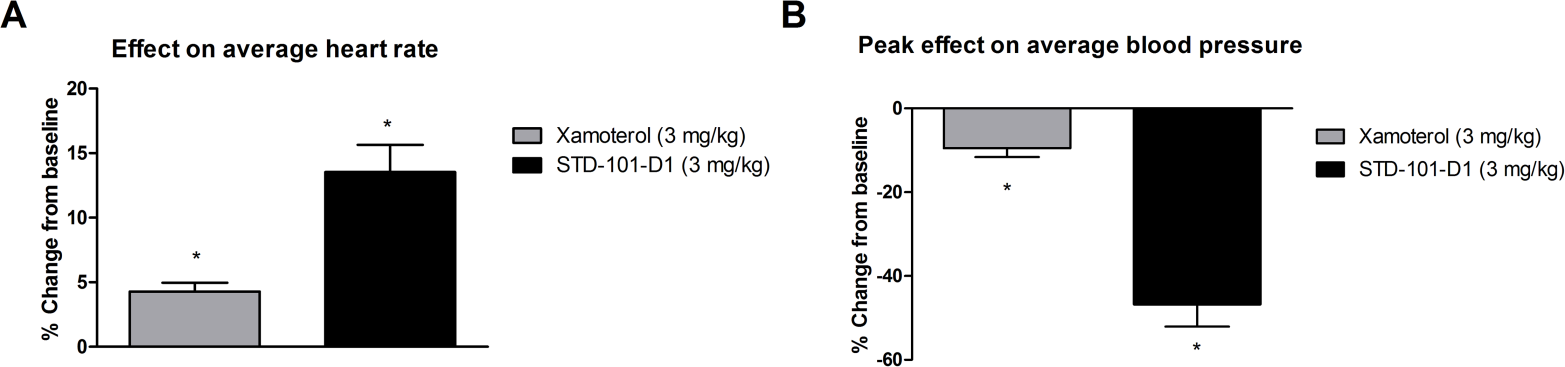
Effects of xamoterol and STD-101-D1 on heart rate and blood pressure. Changes in heart rate (A) and blood pressure (B) following subcutaneous administration of xamoterol or STD-101-D1 at dose of 3 mg/kg. Date are represented as mean ± SEM (n = 3 per compound). (One-sample t-test vs. 0% theoretical mean, * p < 0.05).

ADRB1 is highly expressed in cardiac tissue and plays an important role in regulating cardiac function. To determine if xamoterol and STD-101-D1 has peripheral effects, we evaluated the effects of xamoterol or STD-101-D1 on heart rates and blood pressures in anesthetized rats. At a dose of 3 mg/kg, xamoterol and STD-101-D1 increased heart rates by 4% and 13%, respectively (Fig 16A). In addition, xamoterol and STD-101-D1 decreased blood pressures by 9% and 46%, respectively (Fig 16B).

## Discussion

In this study we established SARs for a novel chemotype targeting the ADRB1. The key compound, STD-101-D1, produced partial agonistic activity on G protein signaling with an EC_50_ value in the low nanomolar range, but engaged very little β-arrestin signaling compared to the unbiased agonist isoproterenol. This biased ligand represents a new mode for ADRB1 activation and is distinctly different compared to isoproterenol—a full and unbiased agonist.

ADRB1 has been known to play an important role in learning and memory functions. For example, the ADRB1 selective antagonist betaxolol has been shown to induce contextual memory impairment in mice, which was reversed by the ADRB1 selective partial agonist xamoterol in a dose-dependent manner [10]. Similarly, the retrieval deficits exhibited by mice with NA deficiency have been rescued by the ADRB1 selective partial agonist xamoterol [27]. The involvement of ADRB1 in learning and memory has important clinical and therapeutic implications for AD, as severe neurodegeneration of the NA system begins in the early stages of AD. It is believed that loss of NA signaling and the resulting hypoactivation of ADRB1 may partially contribute to the cognitive symptoms in AD. Therefore, ADRB1 agonists may provide a promising therapeutic strategy to improve cognitive function in AD by restoring the lost NA signaling. Given the well-characterized role of protein kinase A (PKA)/cAMP response element-binding protein (CREB) signaling in learning and memory [28, 29], the cyclic adenosine monophosphate (cAMP) signaling pathway downstream of ADRB1 is believed to mediate the cognitive-enhancing effects of ADRB1 agonists. Toward our goal of modulating ADRB1 to produce therapeutic benefits in AD, we specifically sought to discover partial agonists; these would have more subtle effects in the periphery yet be efficacious enough to restore the decreased NA signaling found in AD. Indeed, human trials with ADRB1 partial agonists such as xamoterol were shown to be very safe, suggesting lack of toxicity with this class of compounds [30, 31]. Accordingly, we chose xamoterol, a known partial agonist of ADRB1, as our lead compound and exploited SAR around its analogs.

The SAR studies described here showed that, in general, substitution of the phenol ring of xamoterol was not well tolerated. This was especially true for the ortho position to the phenolic OH group, where alkyl groups and Cl led to a complete loss of activity. This appeared to be largely a steric effect, as substitution with the small F atom ortho to the OH group gave a compound with only a small diminution in potency. In contrast, substitutions at the morpholino urea site were relatively well tolerated although all were less potent than xamoterol. Aside from the two azetidine urea analogs, all of the compounds showed greater efficacy at the cAMP pathway via ADRB1 compared to xamoterol (S). This effect did not correlate with lipophilicity (cLogP), nor any other obvious structural features of this subset of molecules. It is therefore interesting to speculate that the observed higher efficacy is due to the increased conformational flexibility of the side chains of the non-urea compounds and their consequent ability to fit more readily into the binding sites of the receptor.

Biased agonism, the notion that ligands at GPCRs can preferentially stimulate one intracellular signaling pathway over another, is an emerging concept in GPCR signaling [17, 32, 33]. There is growing interest in the development of biased GPCR ligands, because biased ligands may provide safer and more efficacious therapeutic benefits compared to non-biased ligands by selectively targeting a subset of the receptor-mediated signaling [34]. As a result, the list of known biased agonists for GPCRs including the dopamine D2 receptor, serotonin 5-HT2A, cannabinoid CB1, and κ-opioid receptors, is growing and some have already progressed to clinical development [35–40]. A key property of xamoterol (S) and its analogs (STD-101-D1 through STD-101-D6) is the functional bias toward the G protein-mediated cAMP pathway. G protein-mediated signaling of ADRB1 is implicated in cognitive function and neuroinflammation, whereas β arrestin-mediated signaling is implicated in development of drug-induced tolerance. Therefore, the development of a G protein-biased agonist of ADRB1 may provide a means to optimally tune ADRB1 therapeutics that will ameliorate the cognitive deficits and pathology underlying AD, as well as other neuroinflammatory diseases, without producing significant tachyphylaxis. Biased ligands, such as those reported in this study, can be used as pharmacological tools to aid in the elucidation of ADRB1-mediated signaling cascades in cellular systems and *in vivo*. It will be of great interest to utilize these newly identified functionally selective G protein-biased ADRB1 agonists in additional *in vitro* and *in vivo* assays to determine the contribution of G-protein signaling to ADRB1-mediated effects.

Computational modeling has enabled us to capture poses of the two enantiomers of xamoterol and the ADRB1 agonist STD-101-D1 consistent with experimental data. The docked poses feature polar contacts between the phenolic hydroxyl of xamoterol (S) and both S211 as well as with N310. Furthermore, the binding pocket residue D121 dually forms a salt bridge with the protonated secondary amine of xamoterol’s backbone as well as a hydrogen bond with the beta amino alcohol of xamoterol. The experimental compound STD-101-D1 is predicted to form analogous sets of contacts, with the primary difference originating from the position of its methoxy phenyl ring compared to the heterocyclic ring of xamoterol. Previous studies have identified the contact with S211 as being important for mediating agonism [41]. Further insights into the structure-activity relationships of xamoterol and its derivatives, such as STD-101-D1, would benefit greatly from a fully active, G protein-bound or G protein-mimetic nanobody-bound crystal structure of ADRB1.

Accumulating data suggest a close association between neuroinflammation and AD pathogenesis. Prominent activation of immune responses characterized by activated microglia, reactive astrocytes, and increased expression of complement factors and proinflammatory cytokines associated with Aβ deposits, have been observed in the brains of AD patients as well as in transgenic mouse models of AD [42, 43]. TNFα is one of the main proinflammatory cytokines known to be elevated in brains from AD patients and animal models of AD, and has been strongly implicated in AD pathology. For example, elevated levels of TNFα are observed in serum and cerebrospinal fluid from AD patients compared to age-matched controls [44, 45]. Similarly, overexpression of TNFα has been shown in several animal models of AD, including the 3xTg-AD and 5XFAD mouse models [11, 46, 47]. More importantly, increased TNFα in AD models has been correlated with disease progression [48]. At the molecular level, TNFα has been shown to exacerbate Aβ-induced apoptosis in neurons and increase Aβ production by upregulating both β-secretase expression and γ-secretase activity, as well as the expression of APP [49–53]. It has also been demonstrated that TNFα inhibits phagocytosis of toxic Aβ species, which might lead to hindering efficient plaque removal by brain resident microglia [54]. Taken together, these observations suggest that excessive expression of TNFα may contribute to and accelerate the progression of AD. Targeted inhibition of TNFα signaling in AD may be an effective therapeutic approach to halt or attenuate the progression of AD. In support of this idea, a recent study demonstrates that inhibition of TNFα signaling prevents pre-plaque amyloid-associated neuropathology and reduces plaque accumulation and tau phosphorylation in transgenic mouse models of AD [55, 56]. Relevant to the present study, the adrenergic system has been shown to be involved in the regulation of TNFα signaling as well as general peripheral inflammatory responses and CNS inflammatory responses [57–59]. In our previous *in vitro* study with primary microglia, we showed that the highly selective ADRB1 agonist xamoterol inhibited the LPS-induced TNFα response [11]. Its effects were reversed by the ADRB1 selective antagonists CGP 20712A and betaxolol, but not by the ADRB2 selective antagonist ICI-118551, suggesting that xamoterol produces its anti-inflammatory effects on the TNFα response via ADRB1. When chronically administered to the 5XFAD mouse model of AD, xamoterol also produced anti-inflammatory effects and attenuated increased expressions of proinflammatory markers, including TNFα, shown in the brains of the transgenic mice. This suggests that ADRB1 is an important player in regulating the immune response, and modulating ADRB1 activity has therapeutic potential for AD as well as other neuroinflammatory diseases. Notably, the key compound STD-101-D1 was found to suppress TNFα production in rat primary microglia challenged with LPS. When administered to mice prior to the LPS challenge at 3 mg/kg, STD-101-D1 also attenuated the acute peripheral and CNS TNFα response induced by LPS. Given the well-established suppressive effects of cAMP on LPS-induced transcriptional activation of the TNFα gene [60–62], we speculate that a G protein-biased agonist such as STD-101-D1 acts at the transcription level via a cAMP dependent mechanism. Our observations that the full ADRB agonist isoproterenol or the partial agonist STD-101-D1 did not produce anti-inflammatory effects when administered after the LPS challenge (data not shown), and that they require the pretreatment period to produce inhibitory effects on the LPS-induced inflammatory response, support this hypothesis. However, it should be noted that the current study measures the acute LPS-induced immune response. It will be intriguing to determine whether this class of compound produces anti-inflammatory effects when given after the LPS challenge in the long-term LPS exposure model. It is also important to note that STD-101-D1 has non-specific activity on other receptors and binds to several receptors such as 5-HT 1A and 5-HT 2B with comparable affinity to ADRB1. Thus, it is possible that its anti-inflammatory effects shown *in vitro* and *in vivo* could be mediated by non-ADRB1. Additional studies are necessary to validate that STD-101-D1 exerts its anti-inflammatory effects via ADRB1 and to identify more specific ADRB1 partial agonists. Regardless, identification of ADRB1 agonists with anti-inflammatory effects such as STD-101-D1 has important clinical implications.

Xamoterol is a highly selective partial agonist of ADRB1 with functional bias for the cAMP pathway over the β-arrestin pathway [11]. However, its therapeutic utility as a CNS drug is limited, as it has very poor oral bioavailability and low CNS penetration (Fig 15). Despite this low penetration, however, xamoterol was shown to produce biological effects in the brain after systemic administration. For example, a systemic acute treatment with xamoterol led to increases in phosphorylation of CREB in the brain, an important event involved in learning and memory [9]. In multiple mouse models of AD, chronic systemic administration of xamoterol was also shown to enhance cognitive functions and attenuate pathological features of AD [9, 11]. Based on this observation, we hypothesized that we could maximize the therapeutic utility of this class of drugs for CNS indications by optimizing PK properties of xamoterol. With favorable CNS permeability, ADRB1 agonists can be administered at low doses, which will reduce systemic exposure and lower the peripheral effects, including cardiovascular effects. In order to evaluate the PK properties of STD-101-D1, we conducted *in vitro* microsomal stability tests and *in vivo* PK studies. In the *in vitro* microsome stability test, STD-101-D1 was shown to be very stable, with half-lives greater than 60 min, both in mouse and human microsomes. In rat microsomes, however, STD-101-D1 was relatively less stable and metabolized with a half-life of 25.2 min. This observation indicates a marked species difference in the metabolism of STD-101-D1. Given this faster rate of metabolism in rat liver microsomes compared to mouse liver microsomes, the *in vivo* PK study in rat uses a higher dose of STD-101-D1 (i.e. 10 mg/kg) compared to the dose used in the *in vivo* mouse LPS study (i.e. 3 mg/kg). In the *in vivo* PK study, STD-101-D1 was moderately cleared in Sprague-Dawley rat with a half-life of 2.7 hr after oral administration, in line with its moderate metabolic stability shown in rat microsomes. However, the oral bioavailability of STD-101-D1 remains low (6%), similar to xamoterol. The low oral bioavailability of STD-101-D1 could be due to poor absorption through the gut membrane and/or efflux through the P-glycoprotein.

Obtaining compounds with good brain permeability is a major hurdle in CNS drug development. Importantly, STD-101-D1 shows greater CNS penetration compared to xamoterol (Fig 15). For example, when administered intravenously, STD-101-D1 achieved an approximately 22-fold higher brain concentration compared to xamoterol at 20 min post dose. With its improved CNS permeability, STD-101-D1 can be administered at lower doses for CNS indications, reducing systemic exposure and lowering cardiovascular effects. Of note, the brain concentrations achieved with STD-101-D1 are considered to be within the therapeutically relevant range. For example, when administered intravenously or intraperitoneally, acute single administration of STD-101-D1 at the dose of 10 mg/kg led to brain concentrations of 2.5 μM and 288 nM, respectively. As these values are up to 148-fold higher than its EC_50_ value (16.9 nM) for ADRB1 *in vitro*, we believe brain concentrations achieved with a single administration of STD-101-D1, even at lower doses, would be sufficient to induce the CNS target engagement needed for efficacy. This brain concentration is also in line with other drugs clinically used for AD, such as memantine and donepezil [63, 64]. Collectively, this suggests a possible beneficial therapeutic value of STD-101-D1 for CNS indications, although its overall PK properties remain to be improved. It is important to note that only a trace of STD-101-D1 was detected in the brain at 4 hrs post dose, indicating that most of the drug is cleared from brain within 4 hrs. Further medicinal chemistry efforts would be needed to identify new compounds that have improved oral bioavailability and longer half-lives.

ADRB1 is highly expressed in a number of peripheral organs, and plays an important role in mediating multiple physiological processes. Thus, modulators of ADRB1 could possibly produce many unwanted peripheral adverse effects. Using a medicinal chemistry approach, our laboratory is developing partial agonists of ADRB1 with improved brain penetration and, as a result, we are seeking to decrease peripheral exposure with lower dosing, while achieving efficacious concentrations in the CNS. As our aim was to develop an ADRB1 partial agonist with minimal peripheral effects, we have evaluated the peripheral effects of the key compound STD-101-D1. Notably, at equivalent doses, both xamoterol and STD-101-D1 produced significant changes in heart rates and blood pressures of rats after an acute single administration. The effects of STD-101-D1 were more profound than those of xamoterol, possibility due to its higher efficacy on ADRB1. Importantly, higher brain permeability suggests that we can reduce the dose of STD-101-D1, thereby minimizing peripheral exposure. It has been previously shown that chronic administration of xamoterol (3 mg/kg, subcutaneous administration) does not lead to significant changes in cardiovascular function or cardiac structure in mice [11]. It will be important to determine whether chronic administration of STD-101-D1 at a lower dose leads to changes in cardiovascular function or cardiac structure.

In summary, this study has described the rational design of a novel series of compounds with ADRB1 agonist activity. Our findings indicate that we have successfully identified biased ligands with unique pharmacology. Such biased ligands will be invaluable research tools to dissect out the G protein-coupled receptor signaling transduction mechanisms, and also act as potential lead compounds for further development to provide safer, more efficacious therapeutics.

## Supporting information

S1 Fig HPLC and NMR spectra for xamoterol (S), xamoterol (R), STD-101-B1 to B9, D1 to D6, and E. (PDF)

S1 Table Binding affinity (K_i_, nM) of xamoterol (S) at non-ADRB1 binding sites. (PDF)

S2 Table Binding affinity (K_i_, nM) of STD-101-D1 at non-ADRB1 binding sites. (PDF)
