# Supplementary material for: Discovery of novel brain permeable and G protein-biased beta-1 adrenergic receptor partial agonists for the treatment of neurocognitive disorders
Source: PLoS One. 2017 Jul 26;12(7):e0180319. doi: 10.1371/journal.pone.0180319 (PMC5529018; doi:10.1371/journal.pone.0180319)
Supplement: S1 Fig — (PDF) [file pone.0180319.s001.pdf]

S1 Fig. HPLC and NMR spectra for Xamoterol (S), Xamoterol (R), STD-101-B1 to B9, D1 to D6, and E

Xamoterol (S)

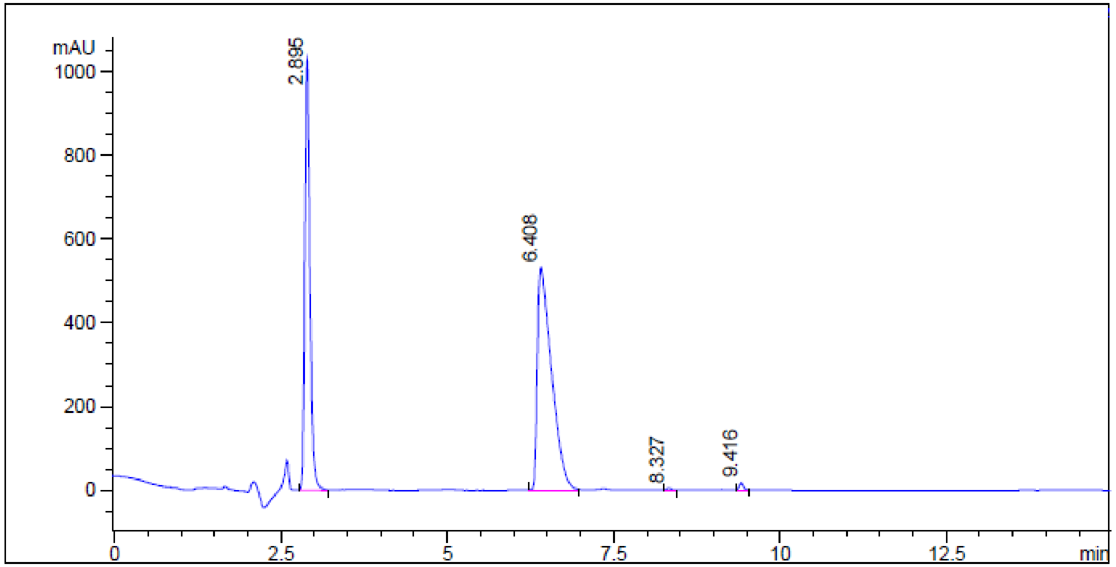

Area Percent Report

Signal 1: DAD1 A, Sig=220,4 Ref=off

Peak #	RT [min]	Type	Width [min]	Height	Area	Area %
1	2.895	MM	0.091	65.167	5617.917	41.359
2	6.408	BB	0.217	33.535	7877.501	57.994
3	8.327	BB	0.064	0.280	18.450	0.136
4	9.416	BB	0.066	1.018	69.460	0.511

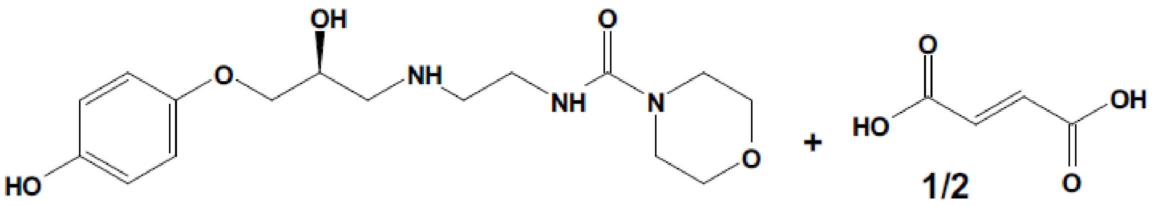

Xamoterol (S)

400MHz ^{1}HNMR DMSO-d_6

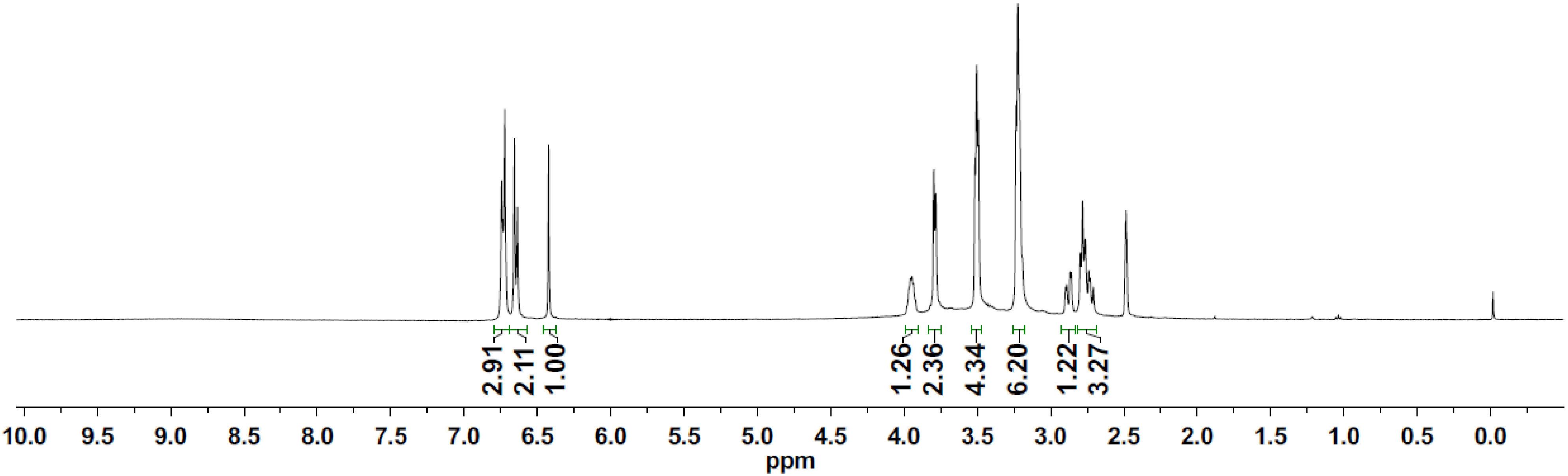

Xamoterol (R)

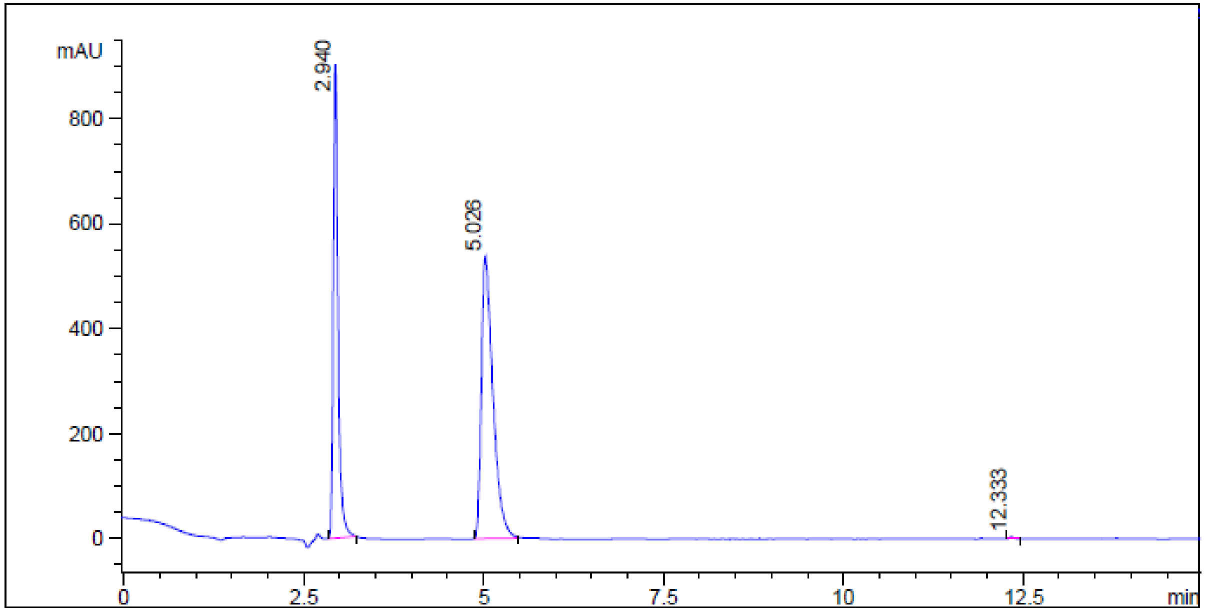

Area Percent Report

Signal 1: DAD1 A, Sig=220,4 Ref=off

Peak #	RT [min]	Type	Width [min]	Height	Area	Area %
1	2.940	MM	0.076	62.591	4116.884	40.822
2	5.026	BB	0.163	37.154	5950.455	59.003
3	12.333	BB	0.074	0.255	17.608	0.175

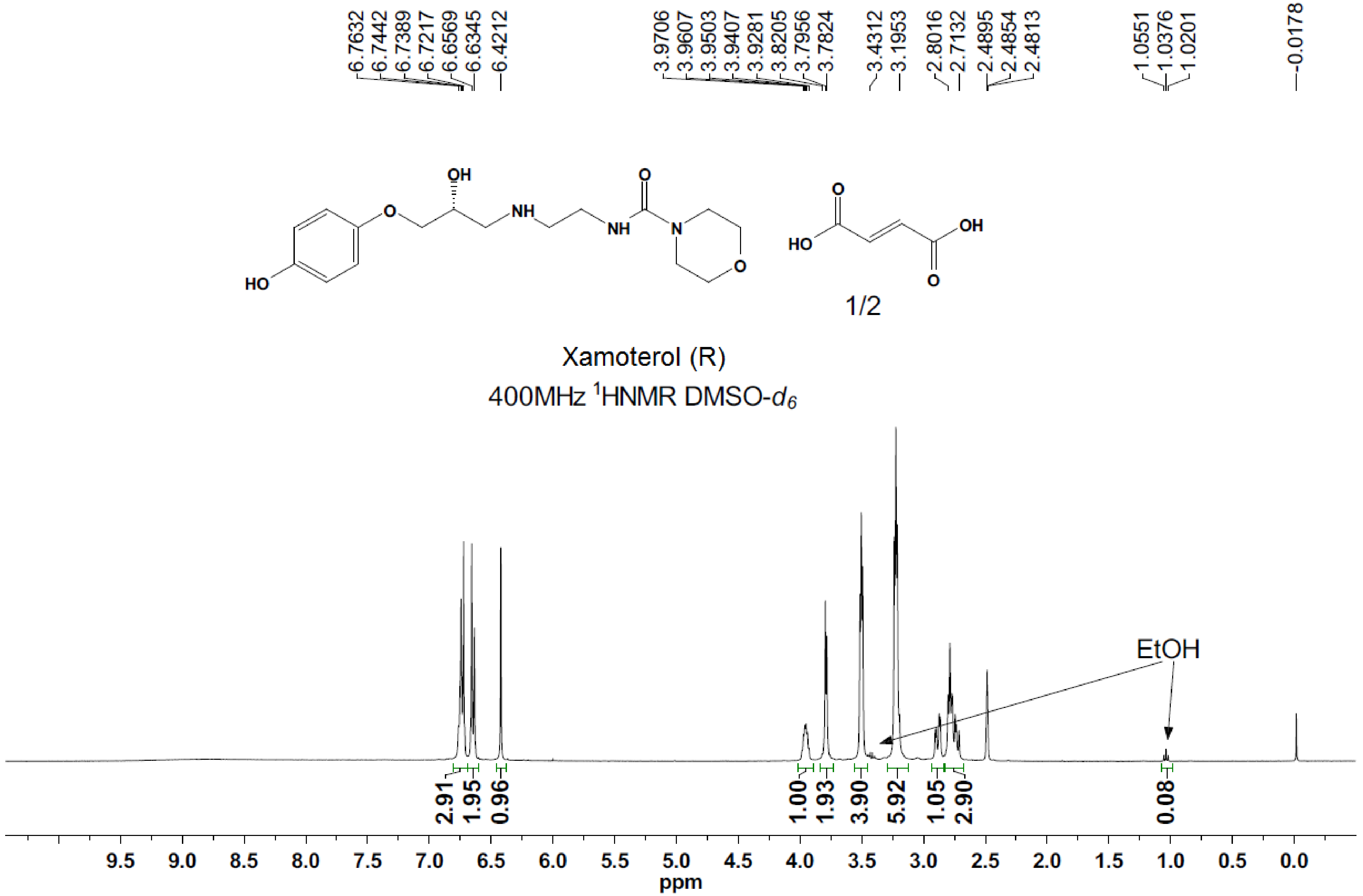

6.7632
6.7442
6.7389
6.7217
6.6569
6.6345
6.4212
3.9706
3.9607
3.9503
3.9407
3.9281
3.8205
3.7956
3.7824
3.4312
3.1953
2.8016
2.7132
2.4895
2.4854
2.4813
1.0551
1.0376
1.0201
-0.0178
OH
O
NH
NH
N
O
HO
O
HO
OH
O
1/2
Xamoterol (R)
400MHz 1HNMR DMSO-d6
EtOH
2.91
1.95
0.96
1.00
1.93
3.90
5.92
1.05
2.90
0.08
9.5
9.0
8.5
8.0
7.5
7.0
6.5
6.0
5.5
5.0
4.5
4.0
3.5
3.0
2.5
2.0
1.5
1.0
0.5
0.0
ppm

STD-101-B1

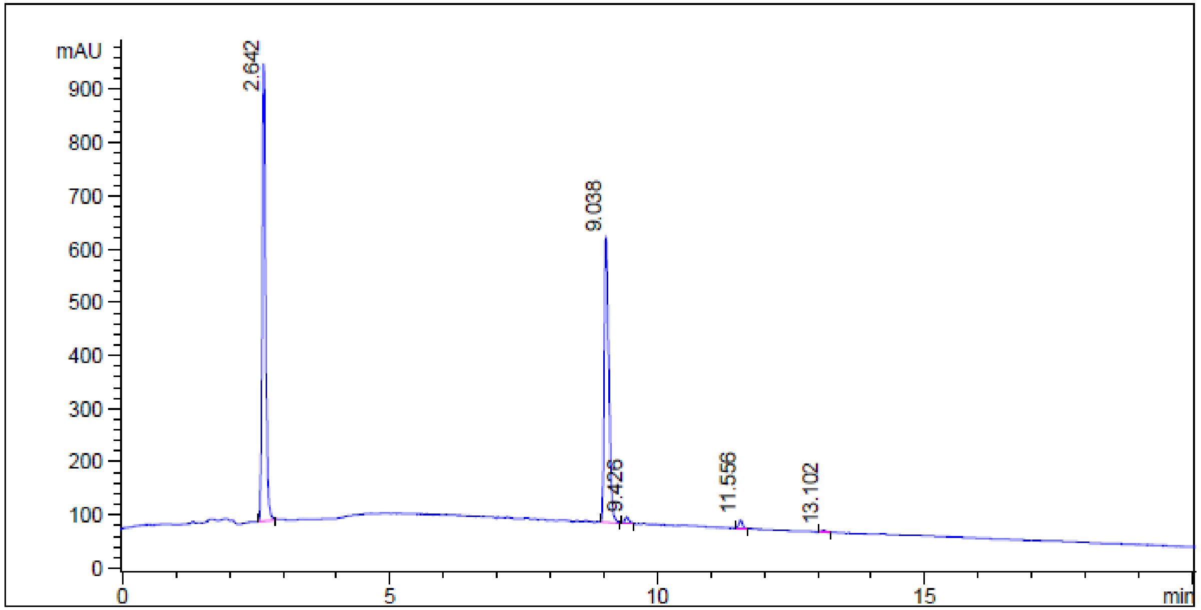

Area Percent Report

Signal 1: DAD1 A, Sig=220,4 Ref=off

Peak #	RT [min]	Type	Width [min]	Height	Area	Area %
1	2.642	BB	0.069	60.396	3951.477	54.116
2	9.038	BB	0.092	37.413	3190.146	43.689
3	9.426	BB	0.080	0.846	63.986	0.876
4	11.556	BB	0.078	1.094	77.589	1.063
5	13.102	BB	0.079	0.252	18.717	0.256

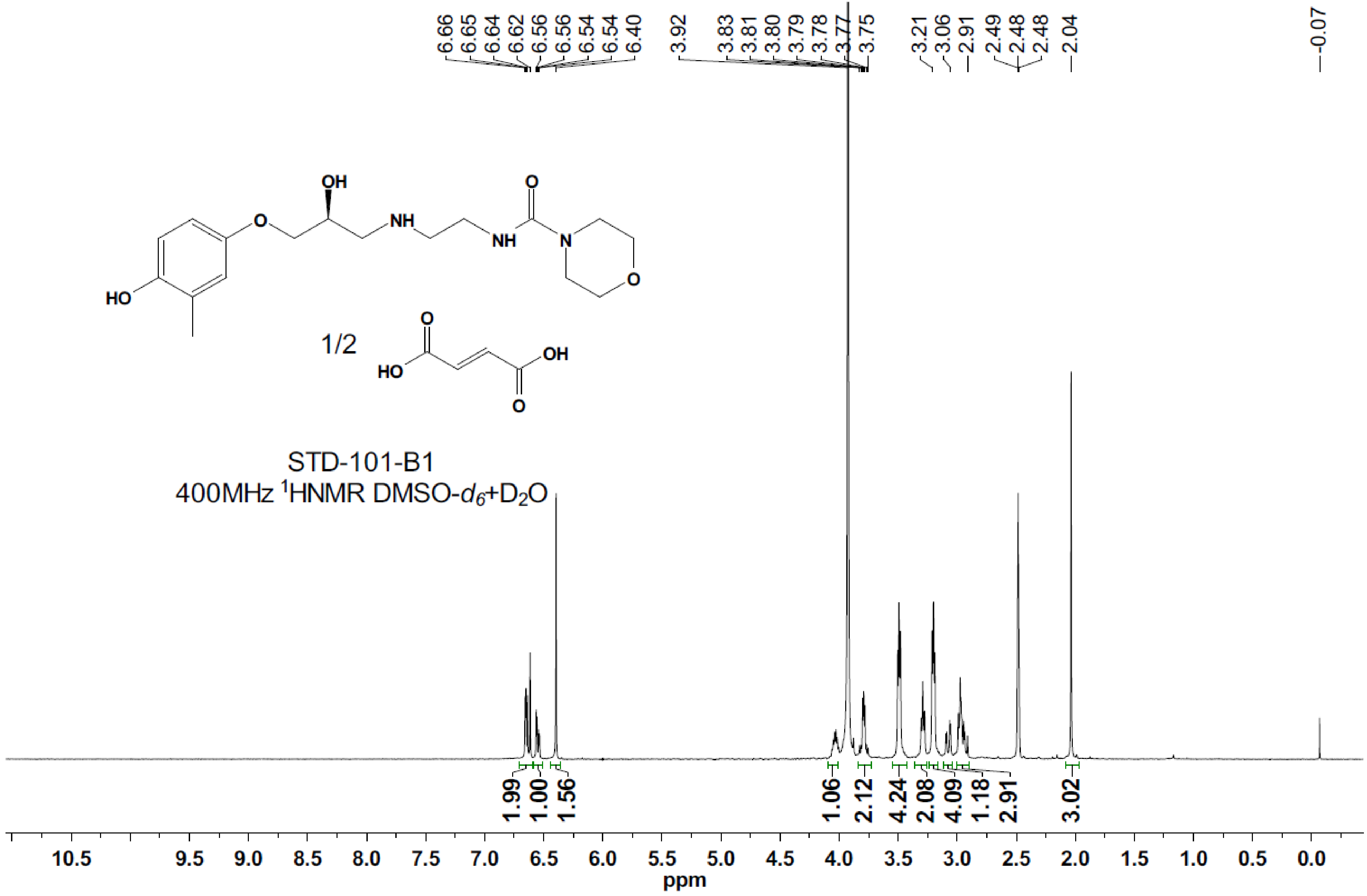
STD-101-B1
400MHz 1HNMR DMSO-d6+D2O
1/2
6.66 6.65 6.64 6.62 6.56 6.56 6.54 6.54 6.40
3.92 3.83 3.81 3.80 3.79 3.78 3.77 3.75
3.21 3.06 2.91 2.49 2.48 2.48 2.04
-0.07
1.99 1.00 1.56 1.06 2.12 4.24 2.08 4.09 1.18 2.91 3.02
ppm

STD-101-B2

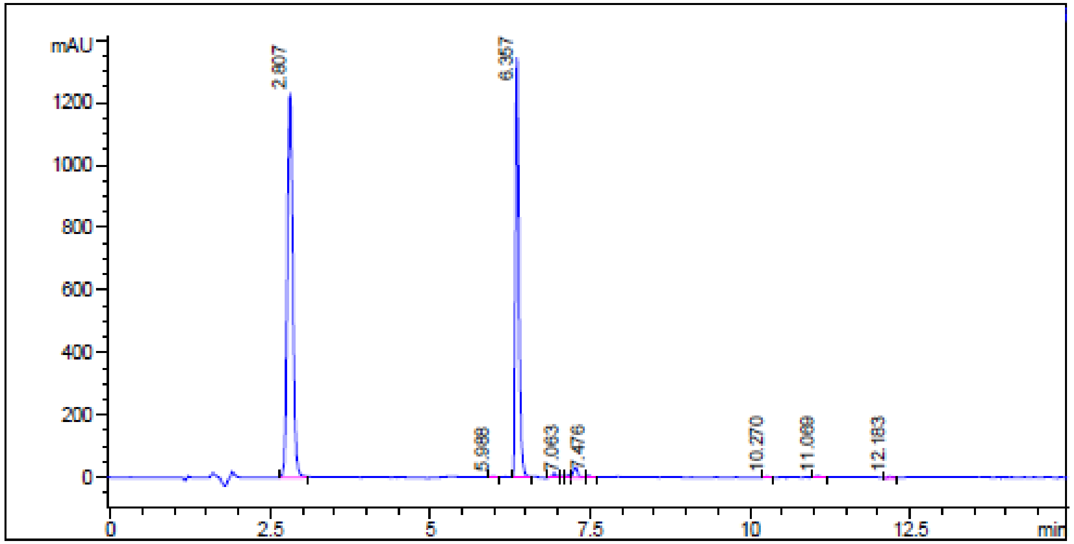

Area Percent Report

Signal 1: DAD1 A, Sig=220,16 Ref=off

Peak #	RT [min]	Type	Width [min]	Height	Area	Area %
1	2.807	BB	0.093	46.136	7235.272	55.747
2	5.988	BB	0.059	0.108	11.110	0.086
3	6.357	BB	0.063	50.758	5379.355	41.447
4	6.940	BV	0.069	0.529	63.537	0.490
5	7.063	VV	0.061	0.213	22.896	0.176
6	7.156	VV	0.057	0.290	28.395	0.219
7	7.276	VB	0.077	1.259	157.849	1.216
8	7.476	BB	0.058	0.237	23.434	0.181
9	10.270	BB	0.057	0.123	12.150	0.094
10	11.069	BB	0.079	0.168	22.468	0.173
11	12.183	BB	0.071	0.178	22.373	0.172

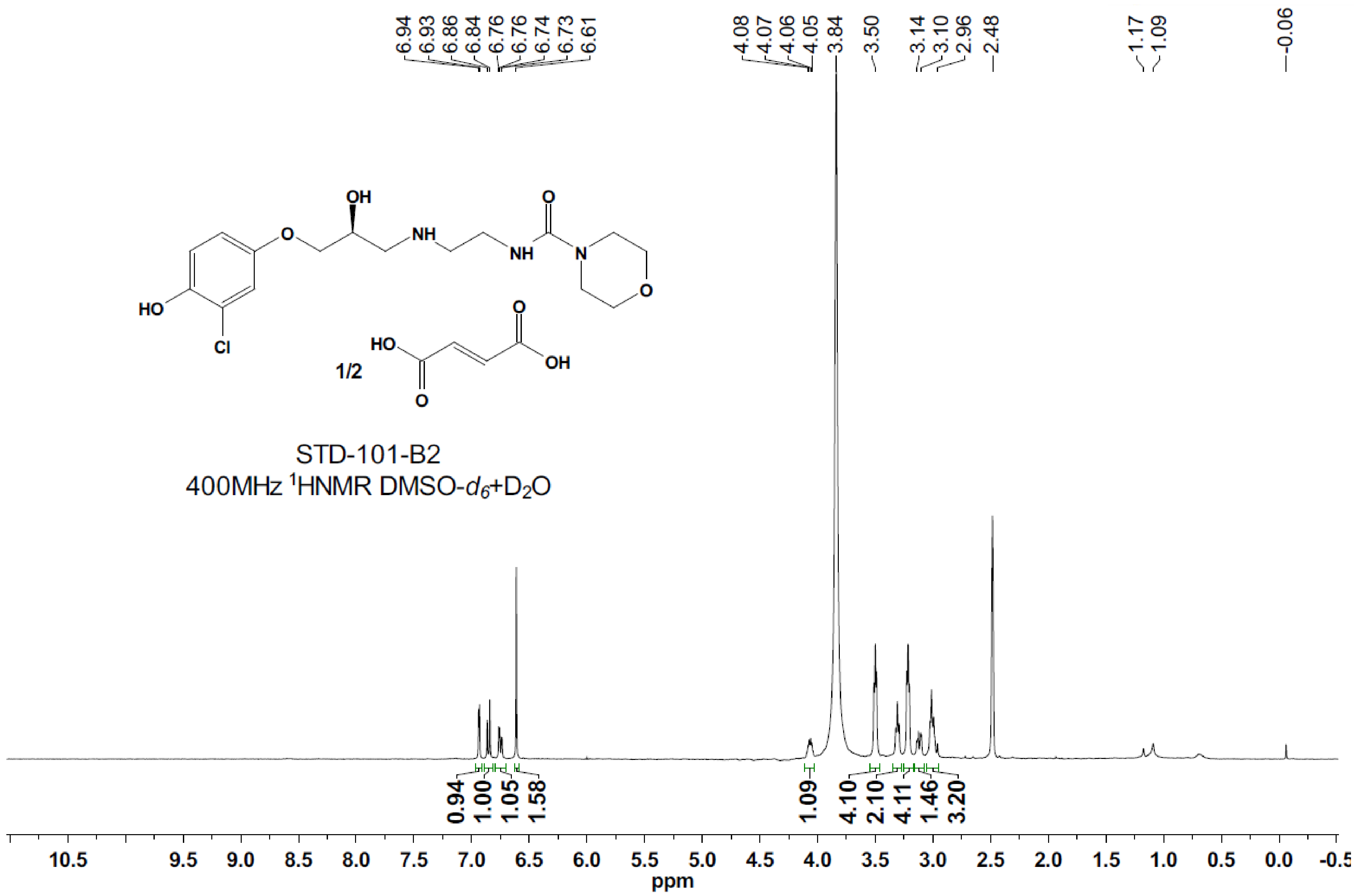
6.94 6.93 6.86 6.84 6.76 6.76 6.74 6.73 6.61
4.08 4.07 4.06 4.05 3.84 3.50 3.14 3.10 2.96 2.48
1.17 1.09
-0.06
OH
O
NH
NH
N
O
HO
Cl
HO
O
OH
1/2
STD-101-B2
400MHz 1HNMR DMSO-d6+D2O
0.94 1.00 1.05 1.58
1.09
4.10 2.10 4.11 1.46 3.20
10.5 9.5 9.0 8.5 8.0 7.5 7.0 6.5 6.0 5.5 5.0 4.5 4.0 3.5 3.0 2.5 2.0 1.5 1.0 0.5 0.0 -0.5
ppm

STD-101-B3

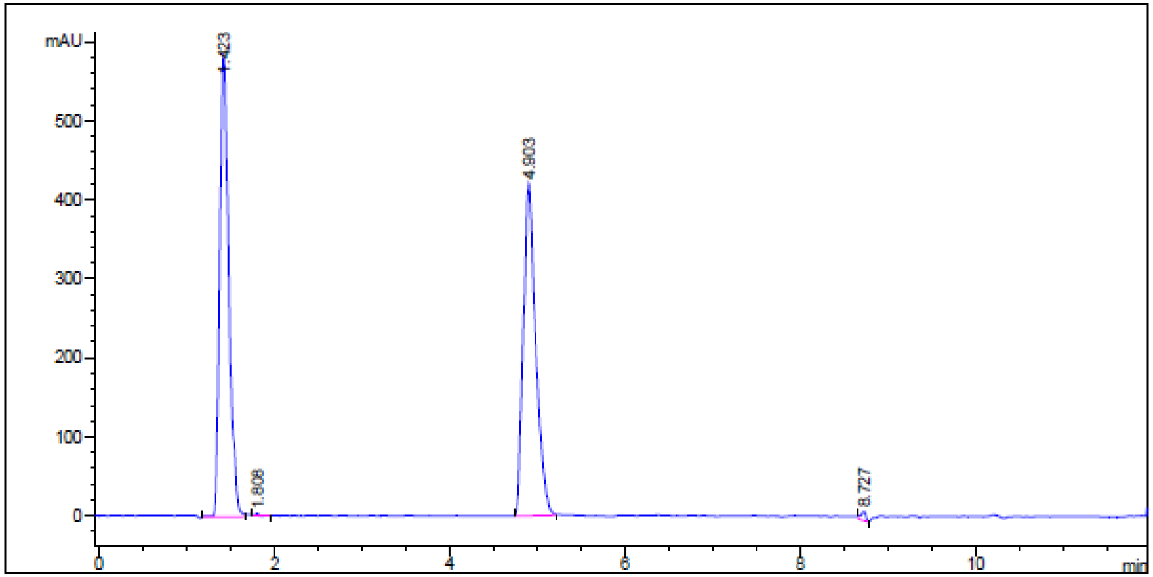

==

Area Percent Report

==

Signal 1: DAD1 A, Sig=220,4 Ref=off
Signal has been modified after loading from rawdata file!

Peak #	RetTime [min]	Type	Width [min]	Area [mAU*s]	Height [mAU]	Area %
1	1.423	BB	0.1118	4198.00293	584.67932	50.1499
2	1.808	BB	0.0675	15.69524	3.29798	0.1875
3	4.903	BB	0.1320	4112.06787	420.65262	49.1233
4	8.727	BV	0.0614	45.14379	11.11092	0.5393

Totals : 8370.90982 1019.74083

==

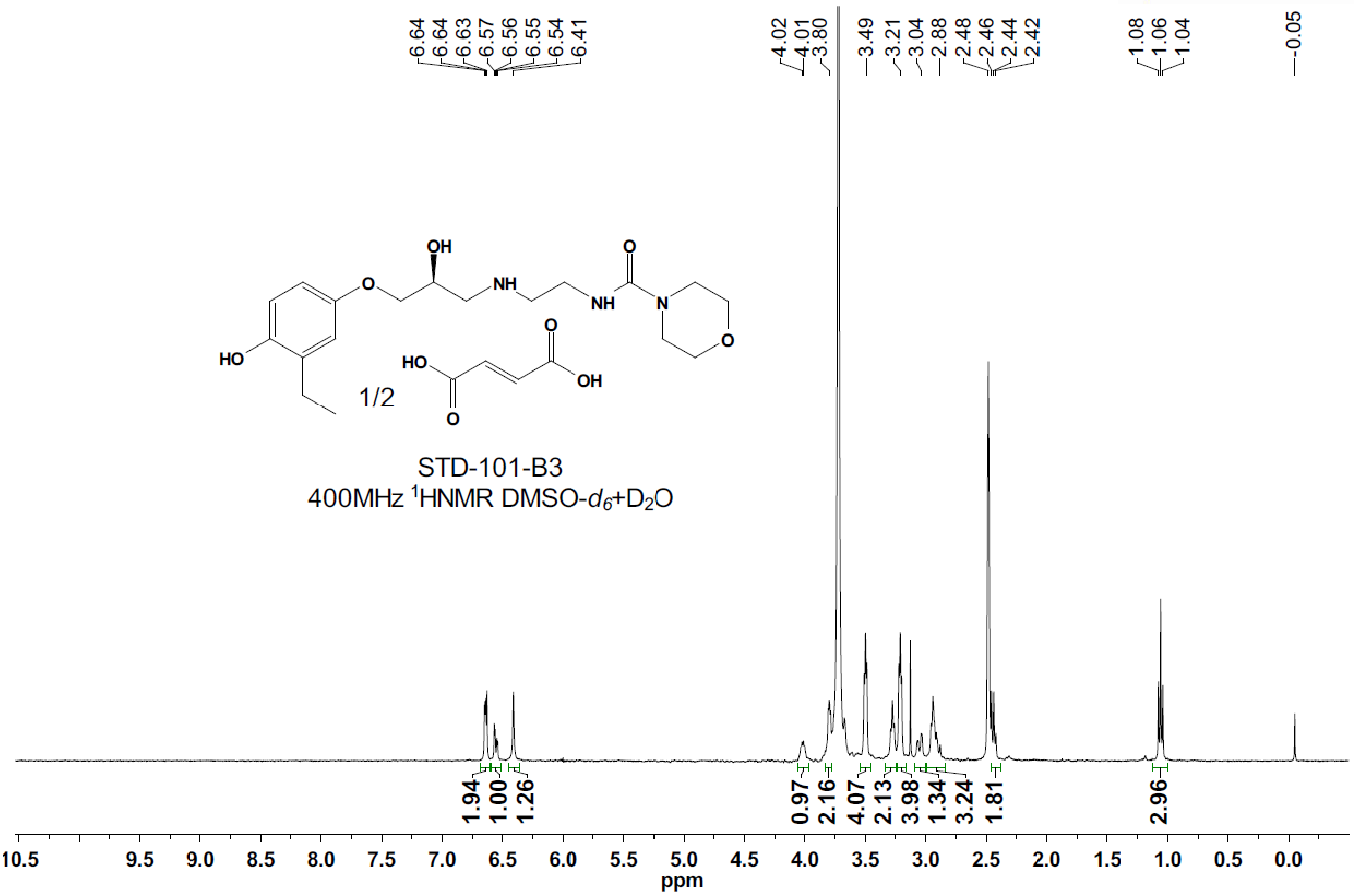
6.64
6.64
6.63
6.57
6.56
6.55
6.54
6.41
4.02
4.01
3.80
3.49
3.21
3.04
2.88
2.48
2.46
2.44
2.42
1.08
1.06
1.04
-0.05
OH
O
NH
NH
N
O
O
HO
HO
OH
O
O
1/2
STD-101-B3
400MHz ¹HNMR DMSO-d6+D2O
1.94
1.00
1.26
0.97
2.16
4.07
2.13
3.98
1.34
3.24
1.81
2.96
10.5
9.5
9.0
8.5
8.0
7.5
7.0
6.5
6.0
5.5
5.0
4.5
4.0
3.5
3.0
2.5
2.0
1.5
1.0
0.5
0.0
ppm

STD-101-B4

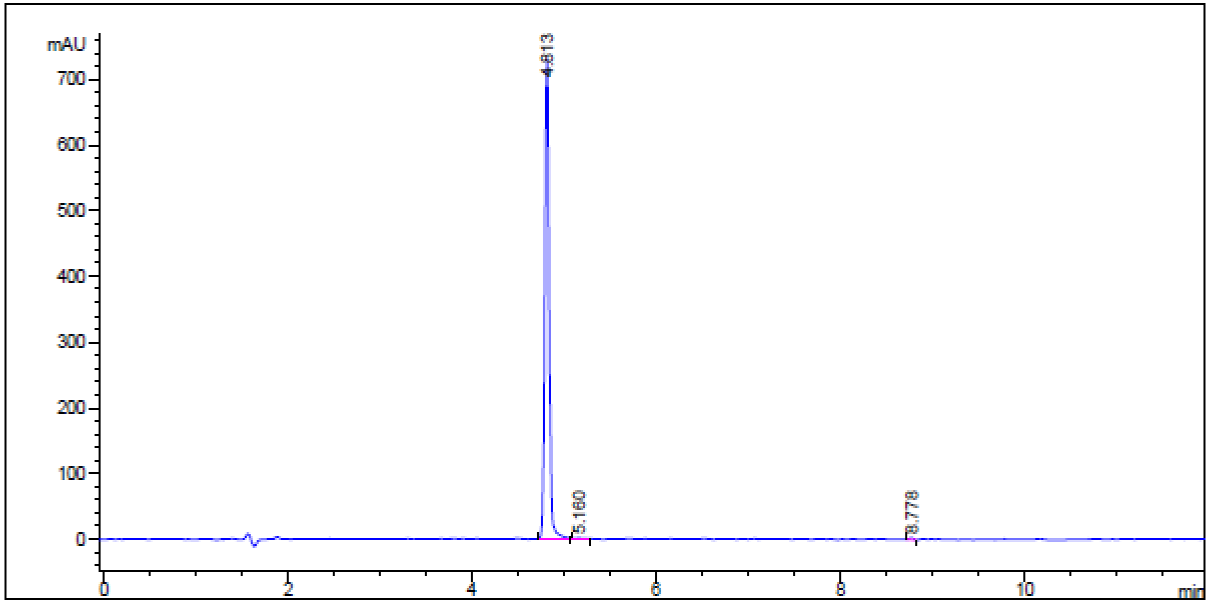

==

Area Percent Report

==

Signal 1: DAD1 A, Sig=220,4 Ref=off
Signal has been modified after loading from rawdata file!

Peak #	RetTime [min]	Type	Width [min]	Area [mAU*s]	Height [mAU]	Area %
1	4.813	BB	0.0527	2439.61719	736.07660	99.0070
2	5.160	BB	0.1032	13.66538	1.71004	0.5546
3	8.778	BV	0.0505	10.80243	3.27725	0.4384

Totals : 2464.08500 741.06389

==

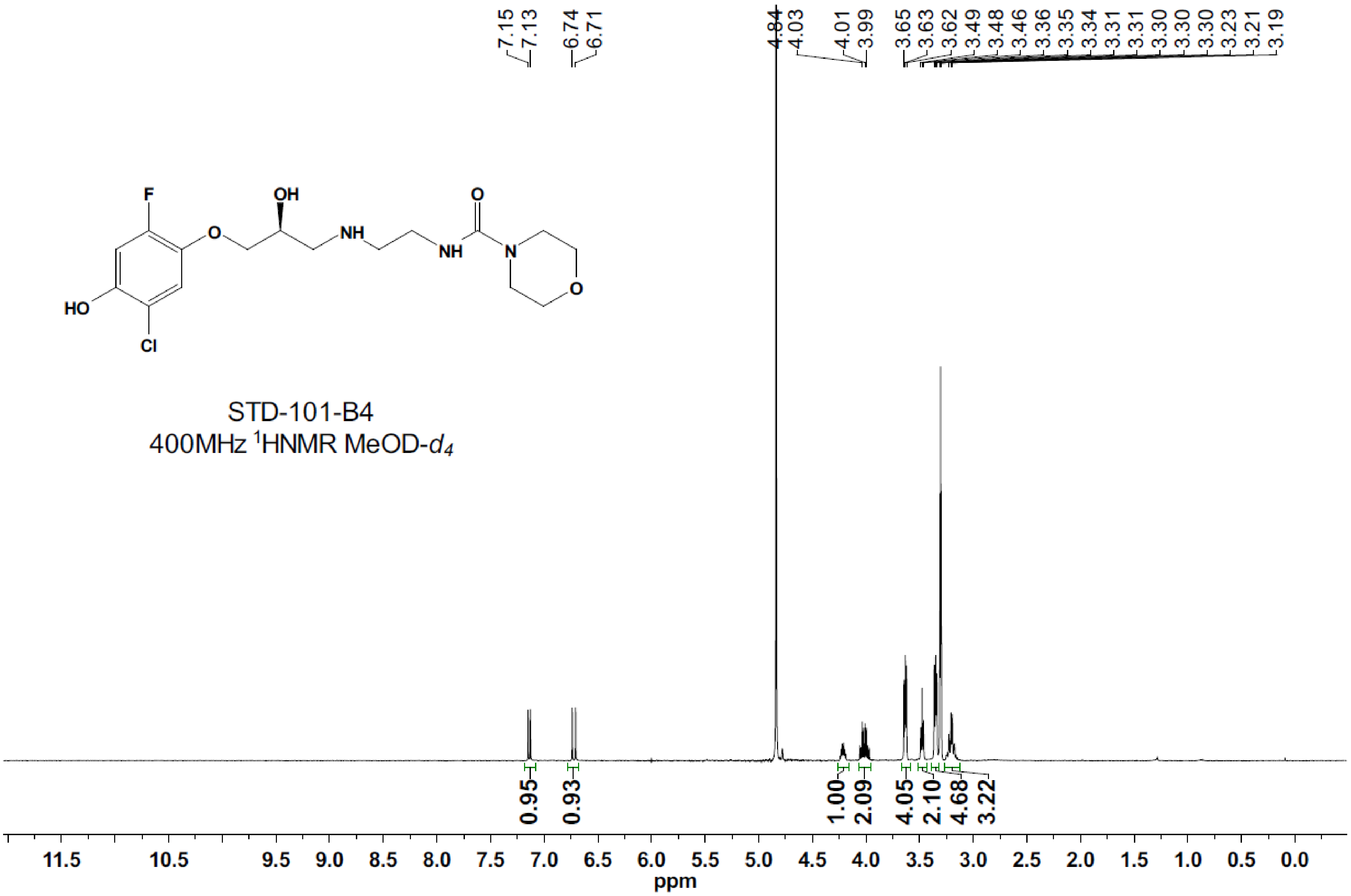

7.15
7.13
6.74
6.71
4.84
4.03
4.01
3.99
3.65
3.63
3.62
3.49
3.48
3.46
3.36
3.35
3.34
3.31
3.31
3.30
3.30
3.30
3.23
3.21
3.19
F
OH
O
NH
NH
O
N
O
HO
Cl
STD-101-B4
400MHz ¹HNMR MeOD-d4
0.95
0.93
1.00
2.09
4.05
2.10
4.68
3.22
11.5
10.5
9.5
9.0
8.5
8.0
7.5
7.0
6.5
6.0
5.5
5.0
4.5
4.0
3.5
3.0
2.5
2.0
1.5
1.0
0.5
0.0
ppm

STD-101-B5

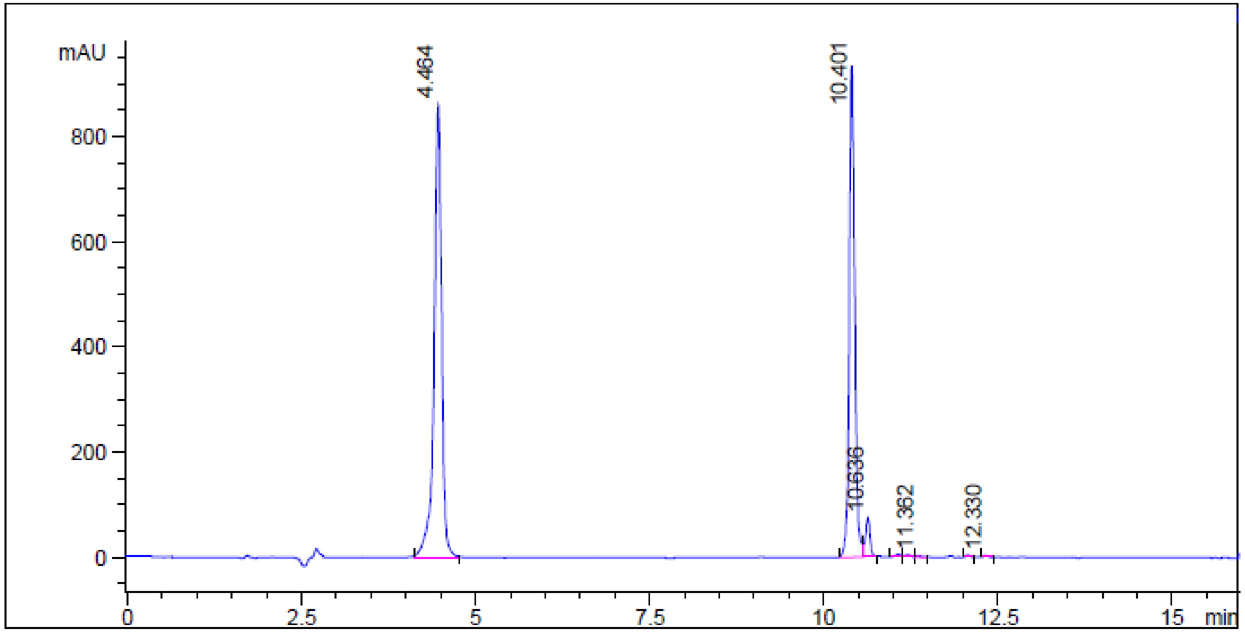

==

Area Percent Report

==

Signal 1: DAD1 A, Sig=220,16 Ref=off

Peak #	RT [min]	Type	Width [min]	Height	Area	Area %
1	4.464	BB	0.117	45.579	6586.832	53.728
2	10.401	BV	0.085	49.459	5232.674	42.682
3	10.636	VB	0.070	3.918	340.150	2.775
4	11.074	BV	0.081	0.264	27.774	0.227
5	11.207	VV	0.093	0.236	27.107	0.221
6	11.362	VB	0.066	0.163	13.753	0.112
7	12.071	BB	0.063	0.206	16.418	0.134
8	12.330	BB	0.068	0.176	14.867	0.121

==

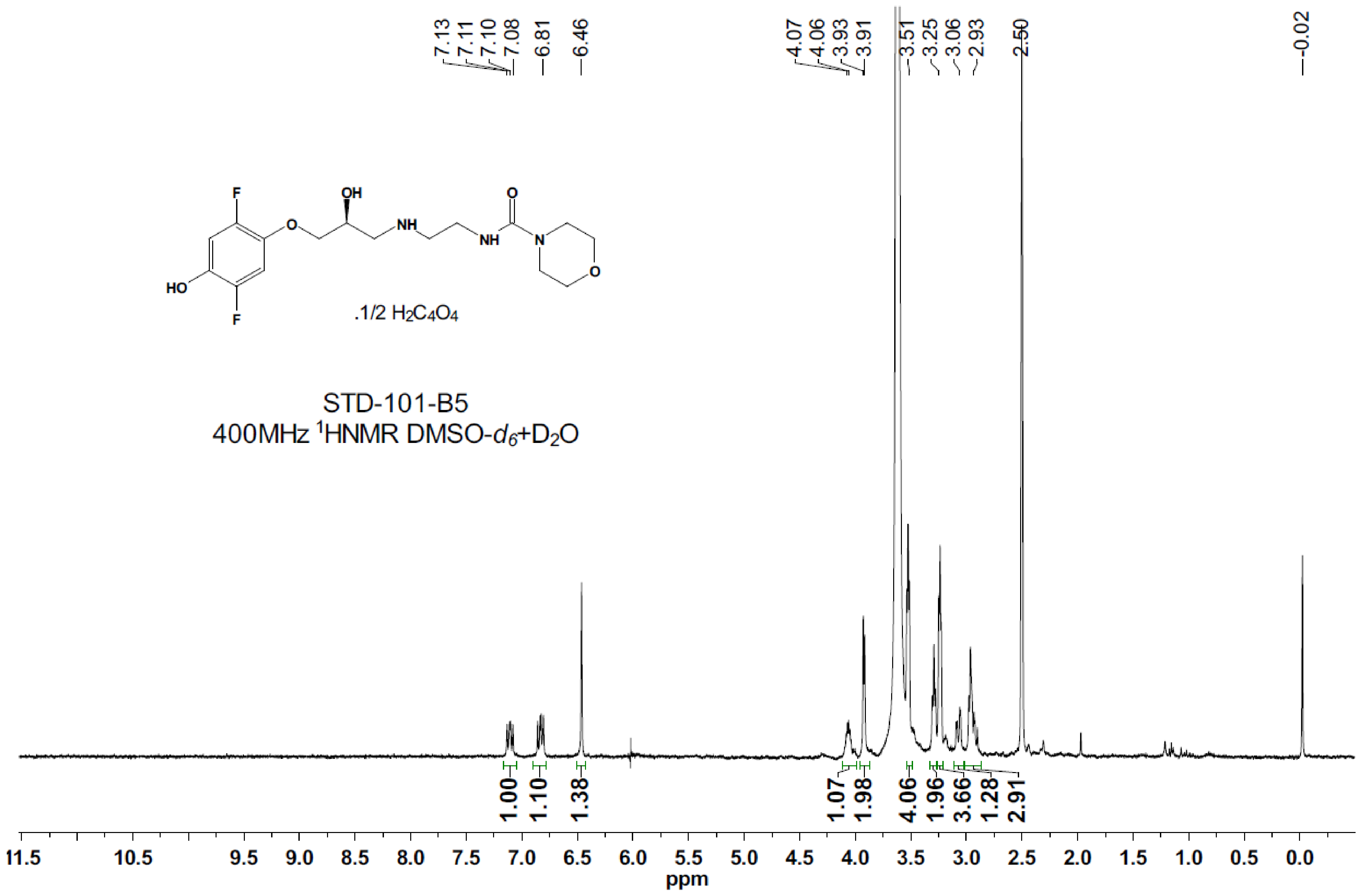

F
OH
O
NH
NH
O
N
O
HO
F
.1/2 $H_2C_4O_4$
STD-101-B5
400MHz ^{1}HNMR DMSO-d_6+D_2O
7.13
7.11
7.10
7.08
6.81
6.46
4.07
4.06
3.93
3.91
3.51
3.25
3.06
2.93
2.50
-0.02
1.00
1.10
1.38
1.07
1.98
4.06
1.96
3.66
1.28
2.91
ppm

STD-101-B6

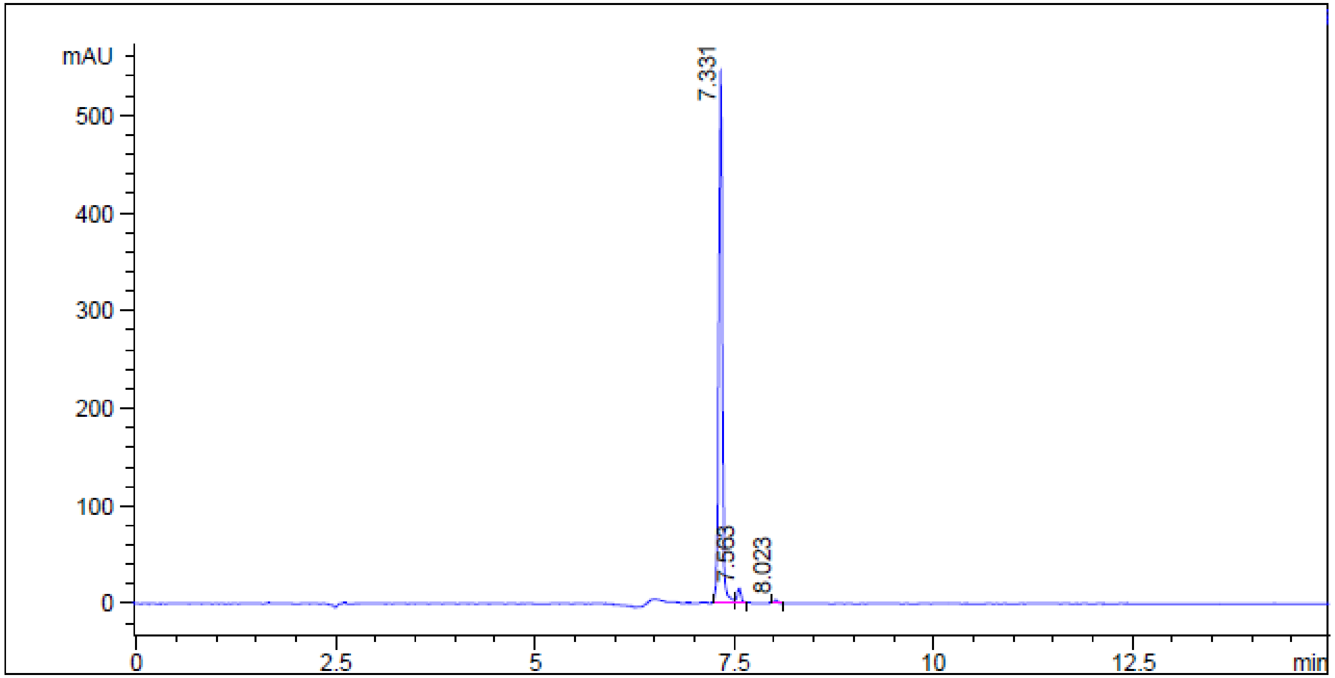

Area Percent Report

Signal 1: DAD1 A, Sig=220,16 Ref=off

Peak #	RT [min]	Type	Width [min]	Height	Area	Area %
1	7.331	BB	0.051	96.713	1824.201	96.575
2	7.563	BB	0.053	2.646	53.425	2.828
3	8.023	BB	0.048	0.642	11.277	0.597

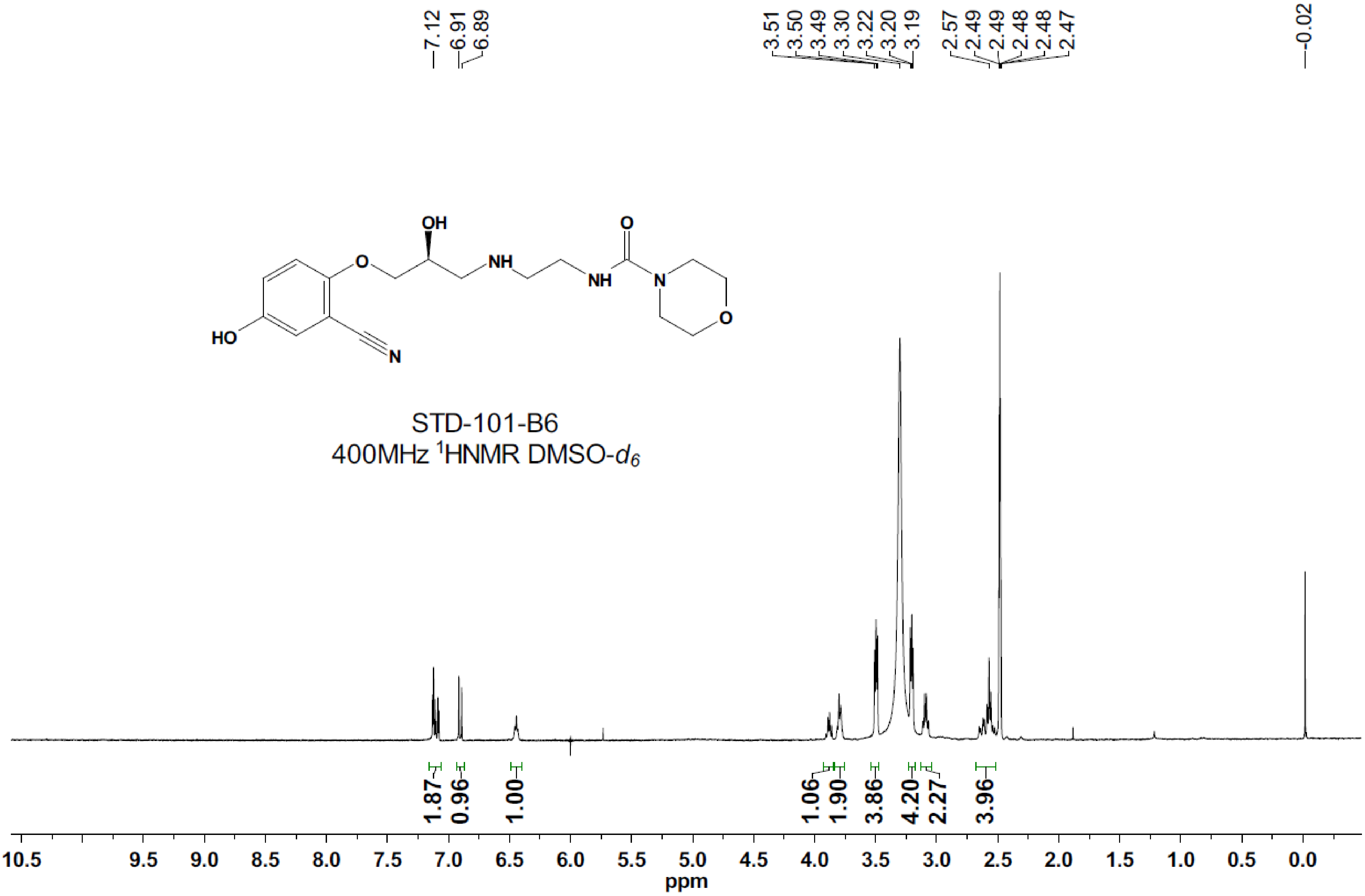
7.12
6.91
6.89
3.51
3.50
3.49
3.30
3.22
3.20
3.19
2.57
2.49
2.49
2.48
2.48
2.47
-0.02
OH
O
NH
NH
O
N
O
HO
N
STD-101-B6
400MHz ^{1}HNMR DMSO-d_6
1.87
0.96
1.00
1.06
1.90
3.86
4.20
2.27
3.96
10.5
9.5
9.0
8.5
8.0
7.5
7.0
6.5
6.0
5.5
5.0
4.5
4.0
3.5
3.0
2.5
2.0
1.5
1.0
0.5
0.0
ppm

STD-101-B7

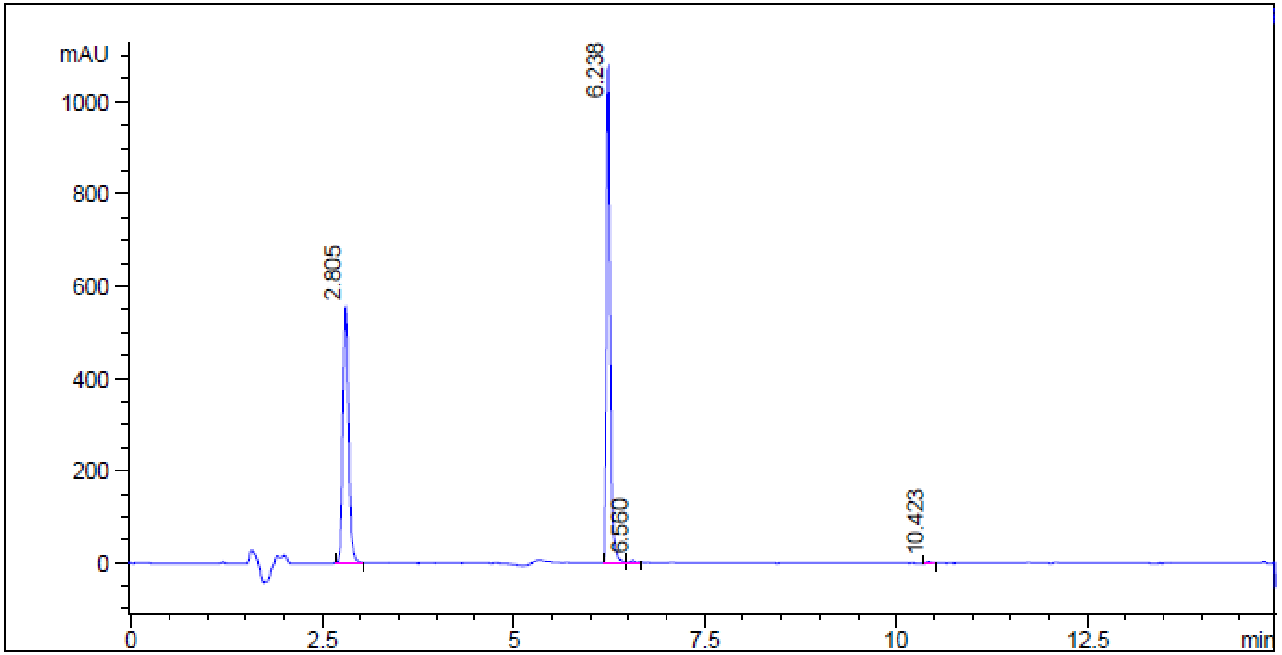

===

Area Percent Report

===

Signal 1: DAD1 A, Sig=220,16 Ref=off

Peak #	RT [min]	Type	Width [min]	Height	Area	Area %
1	2.805	BB	0.078	33.802	2788.547	42.609
2	6.238	BB	0.052	65.673	3719.376	56.832
3	6.560	BB	0.071	0.295	23.590	0.360
4	10.423	BB	0.054	0.231	12.941	0.198

===

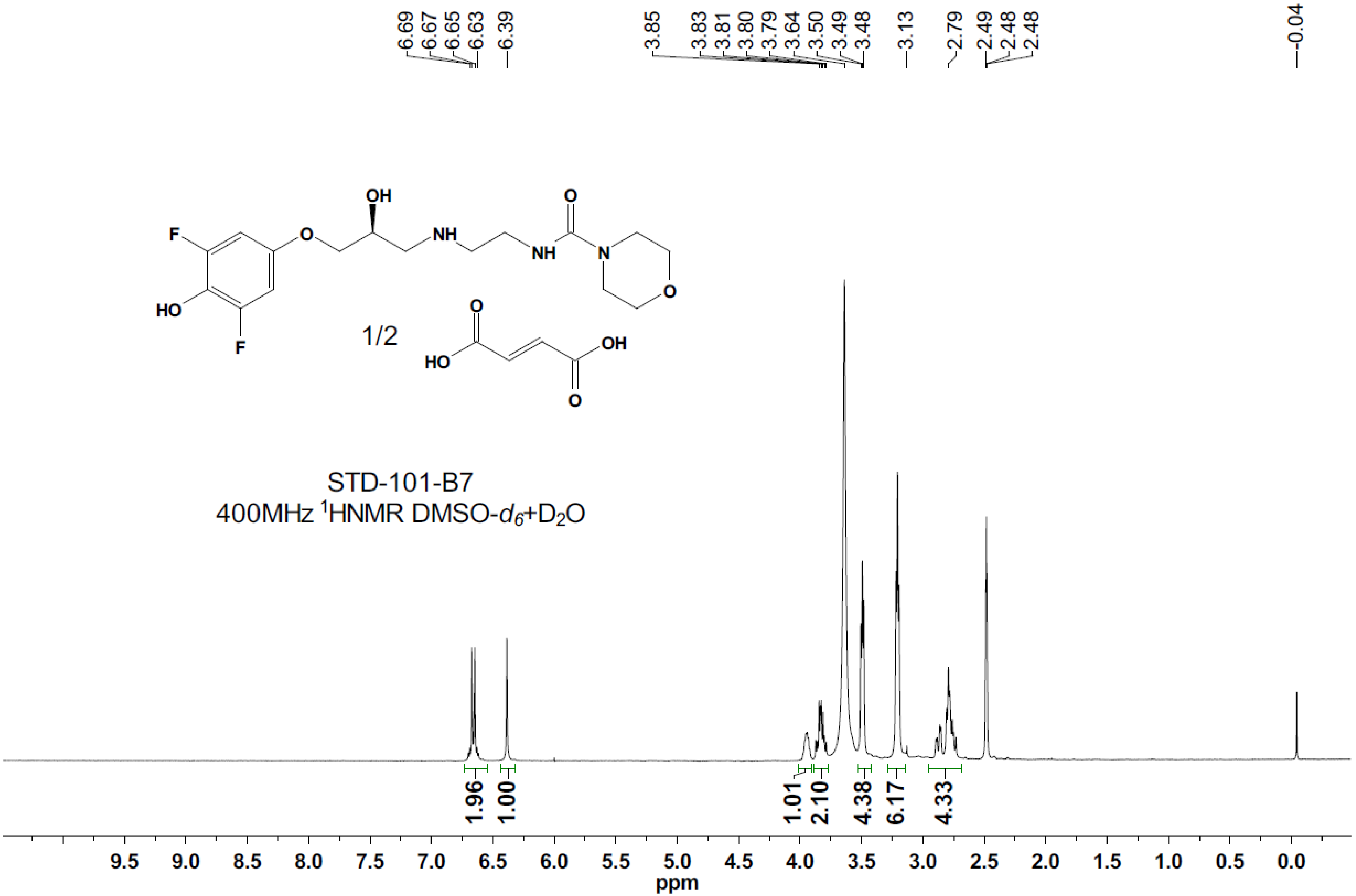
6.69
6.67
6.65
6.63
6.39
3.85
3.83
3.81
3.80
3.79
3.64
3.50
3.49
3.48
3.13
2.79
2.49
2.48
2.48
-0.04
F
OH
O
NH
NH
O
N
O
HO
F
1/2
O
OH
HO
O
STD-101-B7
400MHz 1HNMR DMSO-d6+D2O
1.96
1.00
1.01
2.10
4.38
6.17
4.33
9.5
9.0
8.5
8.0
7.5
7.0
6.5
6.0
5.5
5.0
4.5
4.0
3.5
3.0
2.5
2.0
1.5
1.0
0.5
0.0
ppm

STD-101-B8

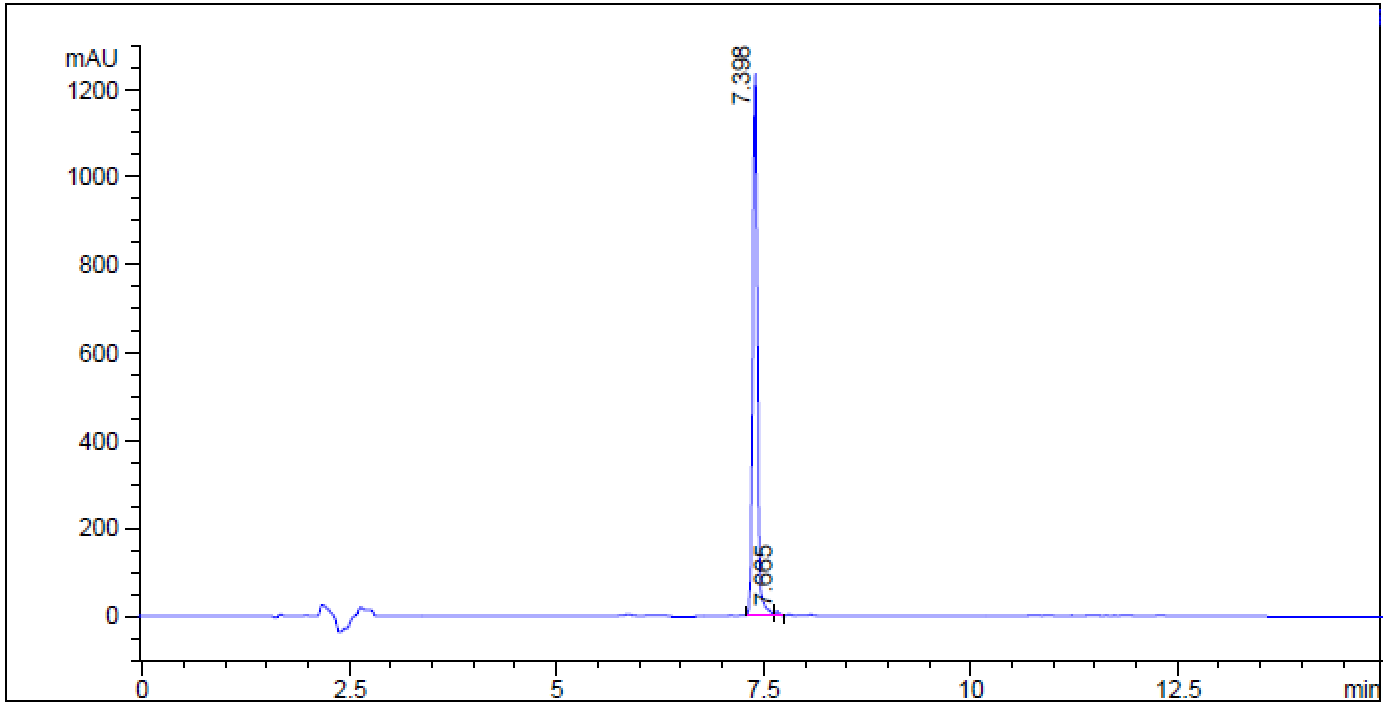

===

Area Percent Report

===

Signal 1: DAD1 A, Sig=220,16 Ref=off

Peak #	RT [min]	Type	Width [min]	Height	Area	Area %
1	7.398	BV	0.059	99.305	4771.844	99.300
2	7.665	VB	0.057	0.695	33.639	0.700

===

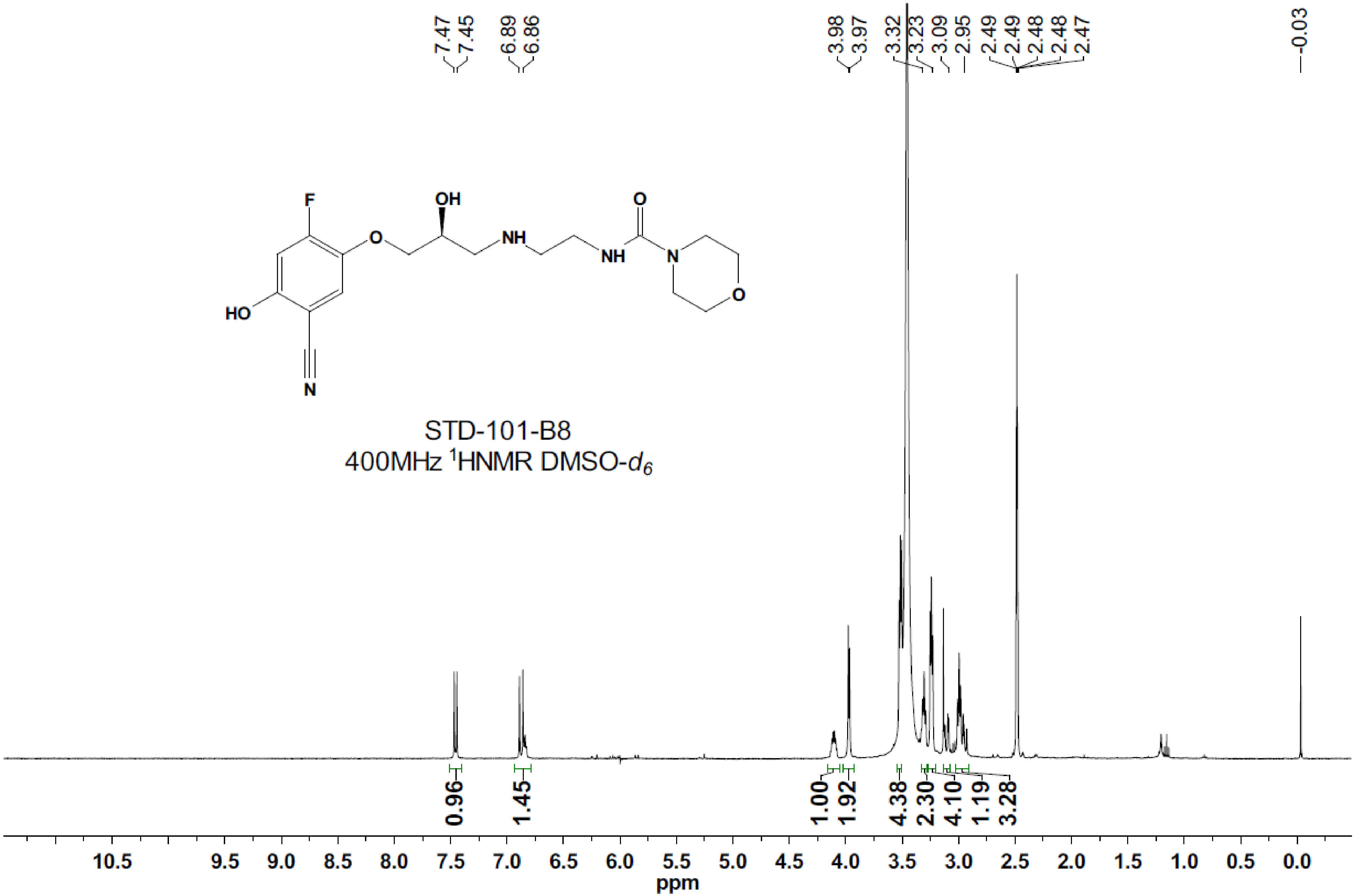

7.47
7.45
6.89
6.86
3.98
3.97
3.32
3.23
3.09
2.95
2.49
2.49
2.48
2.48
2.47
-0.03
F
OH
O
NH
NH
O
N
O
HO
N
STD-101-B8
400MHz 1HNMR DMSO-d6
0.96
1.45
1.00
1.92
4.38
2.30
4.10
1.19
3.28
10.5
9.5
9.0
8.5
8.0
7.5
7.0
6.5
6.0
5.5
5.0
4.5
4.0
3.5
3.0
2.5
2.0
1.5
1.0
0.5
0.0
ppm

STD-101-B9

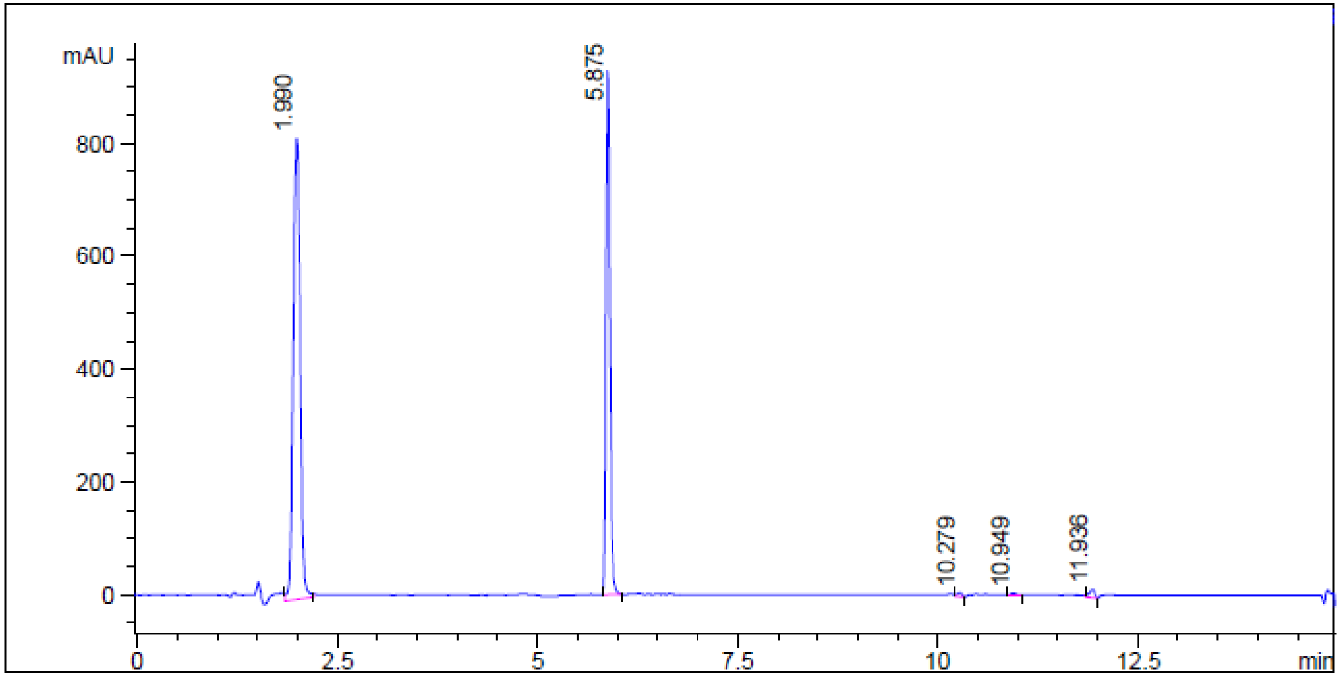

===

Area Percent Report

===

Signal 1: DAD1 A, Sig=220,16 Ref=off

Peak #	RT [min]	Type	Width [min]	Height	Area	Area %
1	1.990	VB	0.098	46.068	5066.942	59.353
2	5.875	BB	0.058	52.609	3369.850	39.474
3	10.279	BV	0.060	0.363	24.326	0.285
4	10.949	BB	0.057	0.167	10.283	0.120
5	11.936	BV	0.070	0.793	65.547	0.768

===

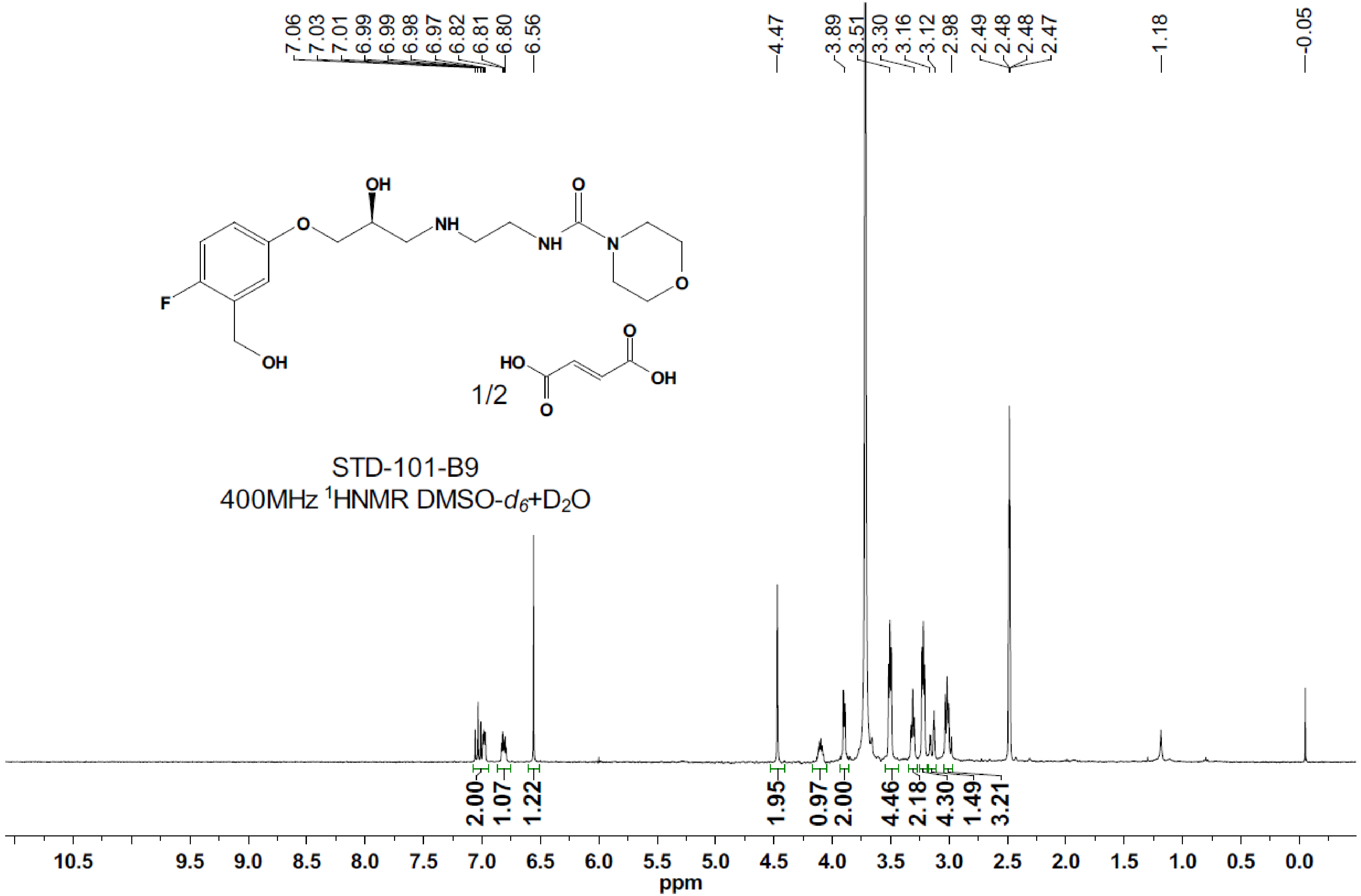
7.06
7.03
7.01
6.99
6.99
6.98
6.97
6.82
6.81
6.80
6.56
4.47
3.89
3.51
3.30
3.16
3.12
2.98
2.49
2.48
2.48
2.47
1.18
-0.05
OH
O
NH
NH
N
O
F
OH
HO
OH
O
O
1/2
STD-101-B9
400MHz 1HNMR DMSO-d6+D2O
2.00
1.07
1.22
1.95
0.97
2.00
4.46
2.18
4.30
1.49
3.21
10.5
9.5
9.0
8.5
8.0
7.5
7.0
6.5
6.0
5.5
5.0
4.5
4.0
3.5
3.0
2.5
2.0
1.5
1.0
0.5
0.0
ppm

STD-101-E

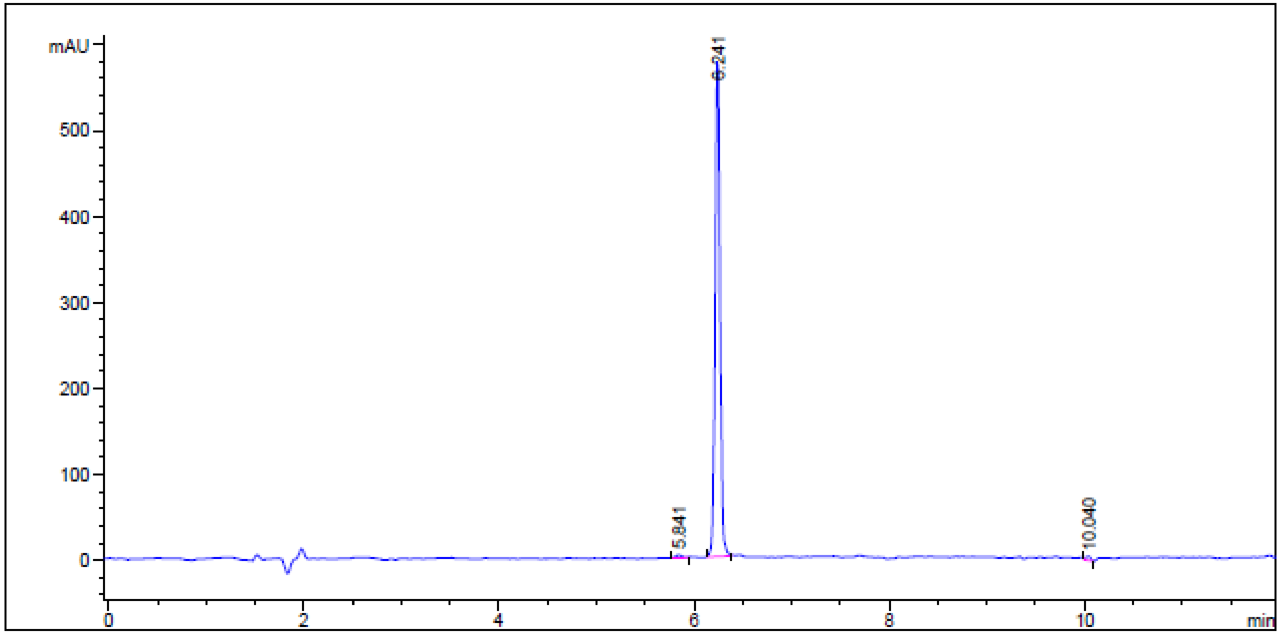

===

Area Percent Report

===

Signal 1: DAD1 A, Sig=220,4 Ref=off

Signal has been modified after loading from rawdata file!

Peak #	RetTime [min]	Type	Width [min]	Area [mAU*s]	Height [mAU]	Area %
1	5.841	BB	0.0694	16.18991	3.40852	0.7785
2	6.241	BB	0.0553	2045.86768	578.03595	98.3760
3	10.040	BV	0.0566	17.58346	4.81107	0.8455

Totals : 2079.64105 586.25554

===

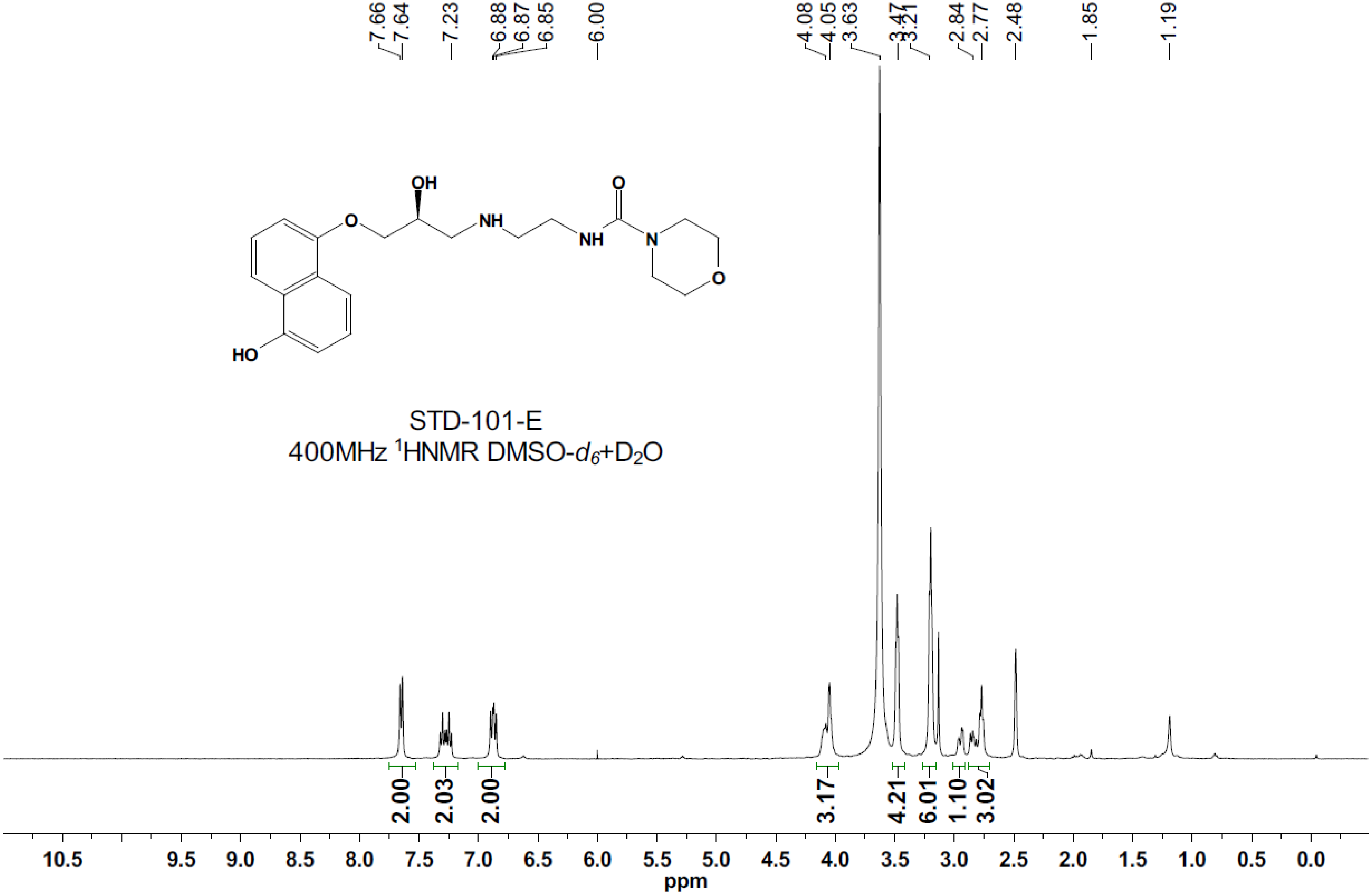
7.66
7.64
7.23
6.88
6.87
6.85
6.00
4.08
4.05
3.63
3.47
3.21
2.84
2.77
2.48
1.85
1.19
OH
O
NH
NH
O
N
O
HO
STD-101-E
400MHz 1HNMR DMSO-d6+D2O
2.00
2.03
2.00
3.17
4.21
6.01
1.10
3.02
10.5
9.5
9.0
8.5
8.0
7.5
7.0
6.5
6.0
5.5
5.0
4.5
4.0
3.5
3.0
2.5
2.0
1.5
1.0
0.5
0.0
ppm

STD-101-D1

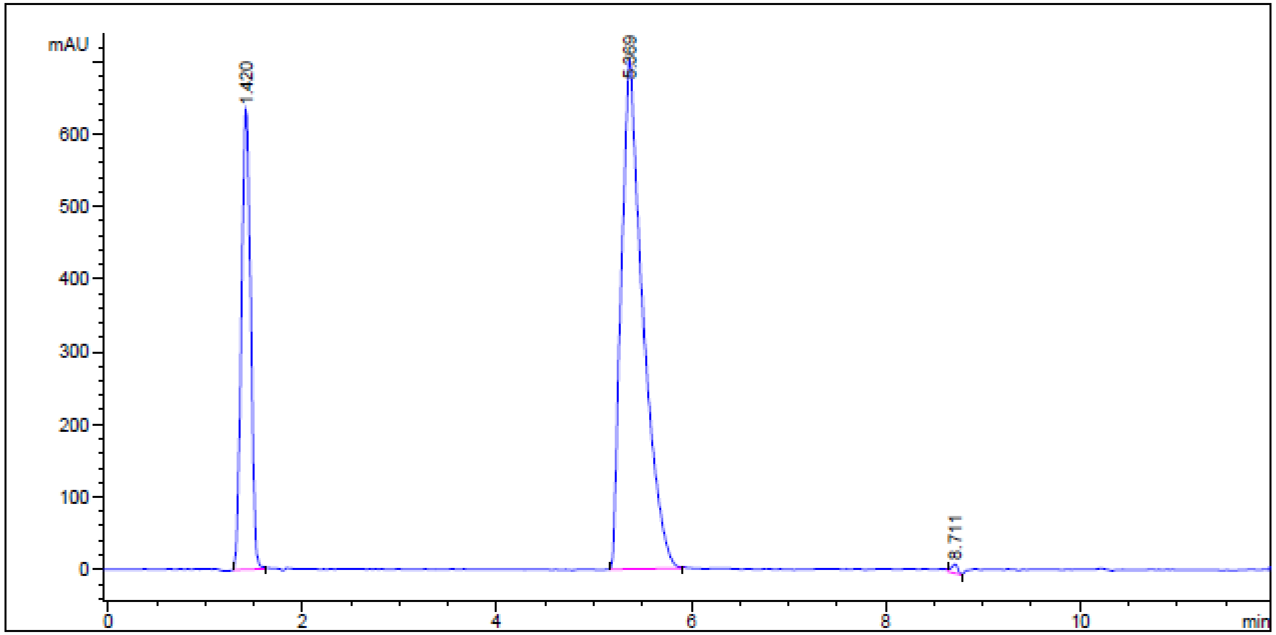

Area Percent Report

Signal 1: DAD1 A, Sig=220,4 Ref=off

Signal has been modified after loading from rawdata file!

Peak #	RetTime [min]	Type	Width [min]	Area [mAU*s]	Height [mAU]	Area %
1	1.420	BB	0.1055	4196.78760	632.01465	28.0859
2	5.369	BB	0.1996	1.06888e4	702.07568	71.5323
3	8.711	BV	0.0719	57.04675	12.34177	0.3818

Totals : 1.49427e4 1346.43210

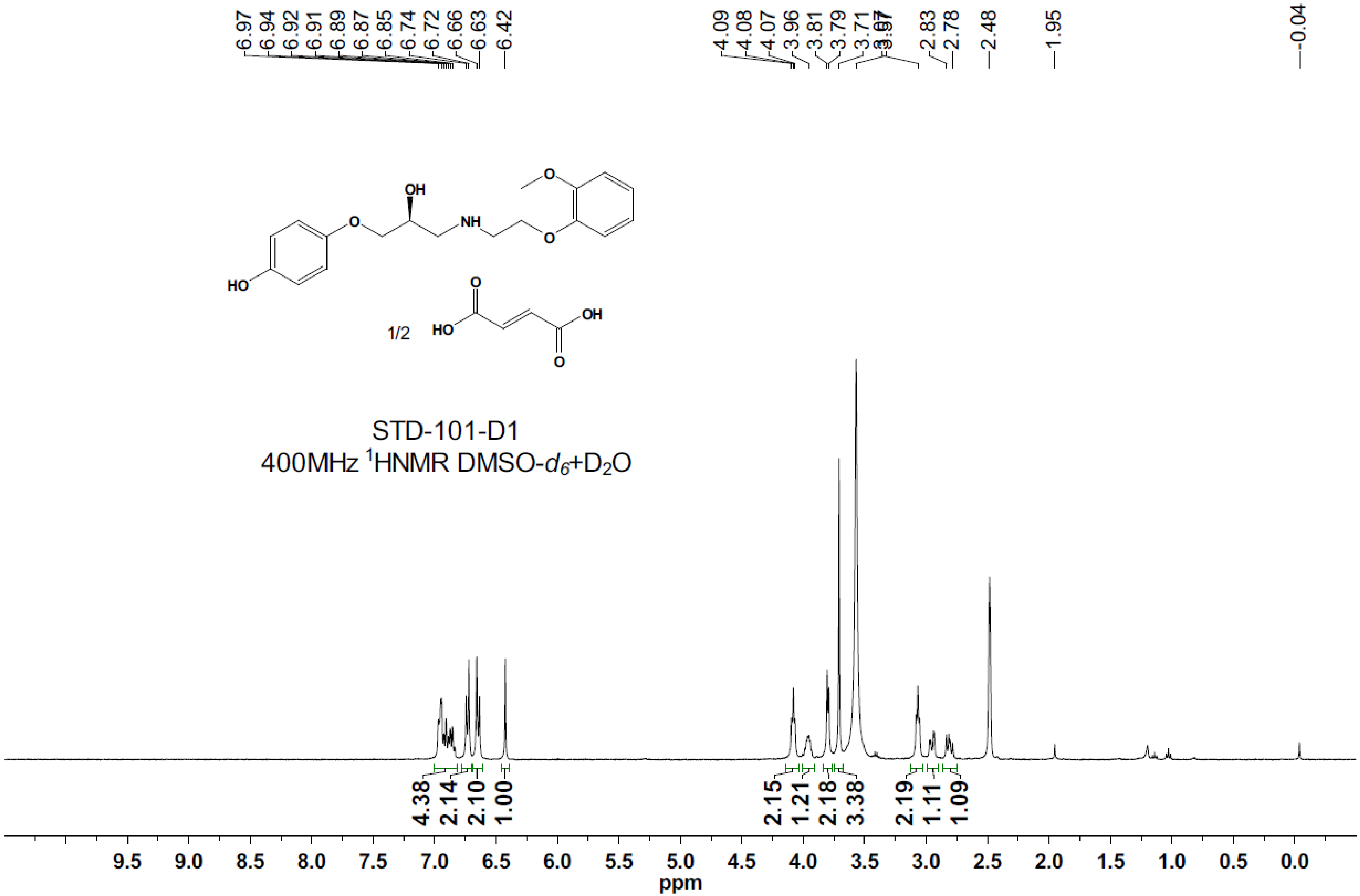
6.97 6.94 6.92 6.91 6.89 6.87 6.85 6.74 6.72 6.66 6.63 6.42
4.09 4.08 4.07 3.96 3.81 3.79 3.71 3.57 2.83 2.78 2.48 1.95 -0.04
OH
O
NH
O
O
HO
1/2
O
HO
OH
O
STD-101-D1
400MHz ¹HNMR DMSO-d6+D2O
4.38 2.14 2.10 1.00
2.15 1.21 2.18 3.38
2.19 1.11 1.09
9.5 9.0 8.5 8.0 7.5 7.0 6.5 6.0 5.5 5.0 4.5 4.0 3.5 3.0 2.5 2.0 1.5 1.0 0.5 0.0
ppm

STD-101-D2

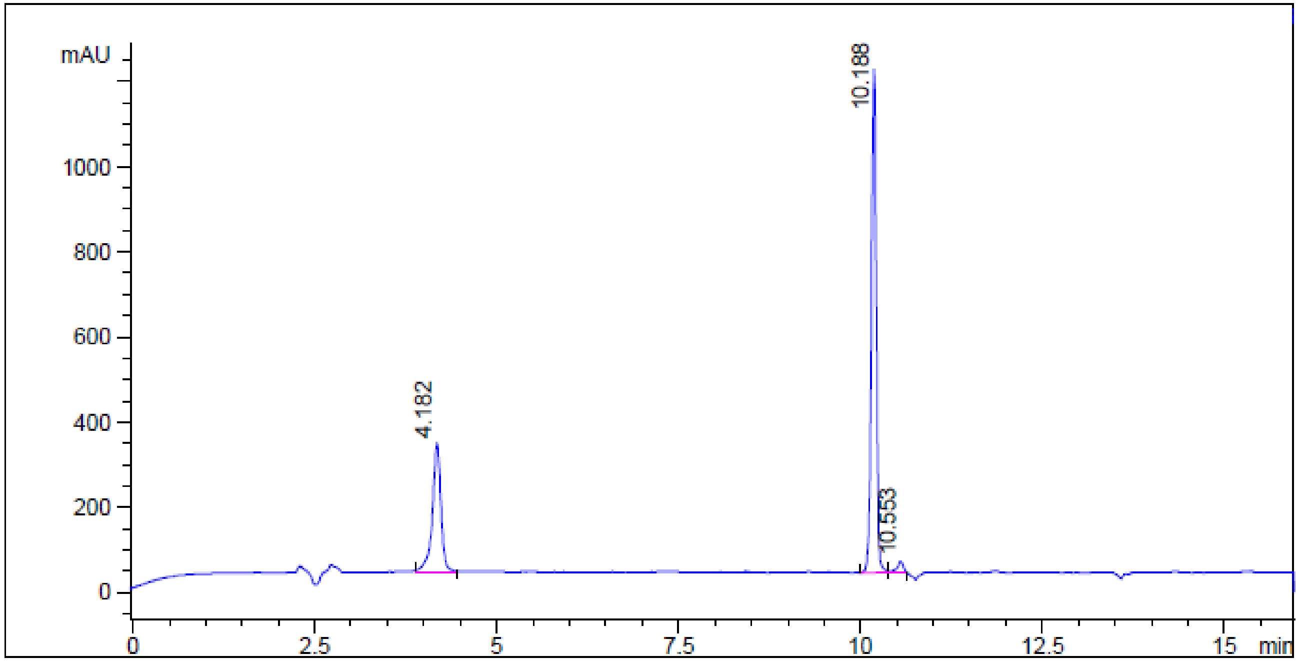

Area Percent Report

Signal 1: DAD1 A, Sig=220,16 Ref=off

Peak #	RT [min]	Type	Width [min]	Height	Area	Area %
1	4.182	BB	0.125	19.850	2489.435	30.895
2	10.188	MF R	0.076	78.427	5413.867	67.189
3	10.553	FM R	0.099	1.723	154.410	1.916

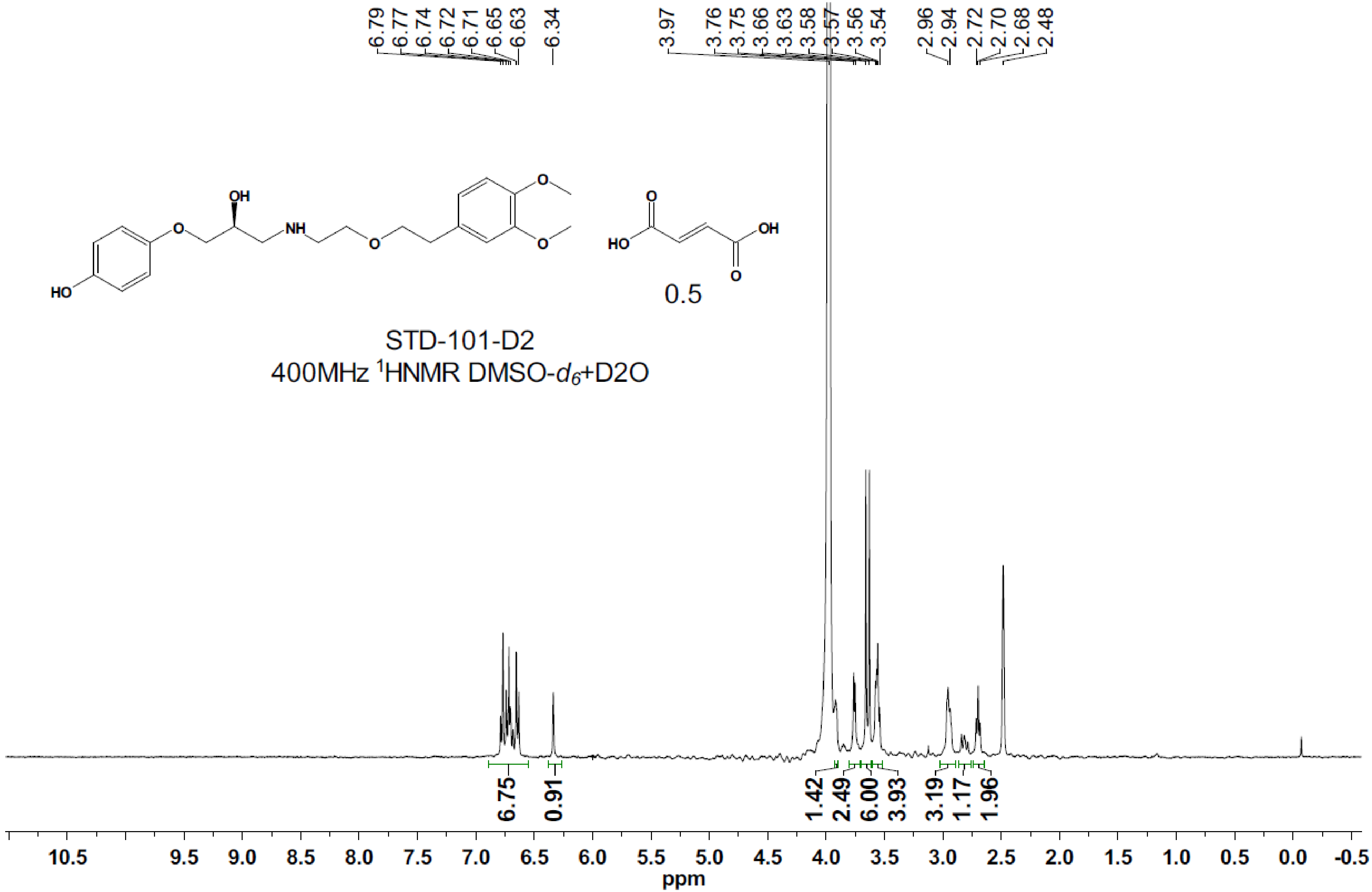
6.79 6.77 6.74 6.72 6.71 6.65 6.63 6.34
3.97 3.76 3.75 3.66 3.63 3.58 3.57 3.56 3.54
2.96 2.94 2.72 2.70 2.68 2.48
OH
O
NH
O
O
O
HO
O
HO
OH
O
0.5
STD-101-D2
400MHz 1HNMR DMSO-d6+D2O
6.75
0.91
1.42
2.49
6.00
3.93
3.19
1.17
1.96
10.5
9.5
9.0
8.5
8.0
7.5
7.0
6.5
6.0
5.5
5.0
4.5
4.0
3.5
3.0
2.5
2.0
1.5
1.0
0.5
0.0
-0.5
ppm

STD-101-D3

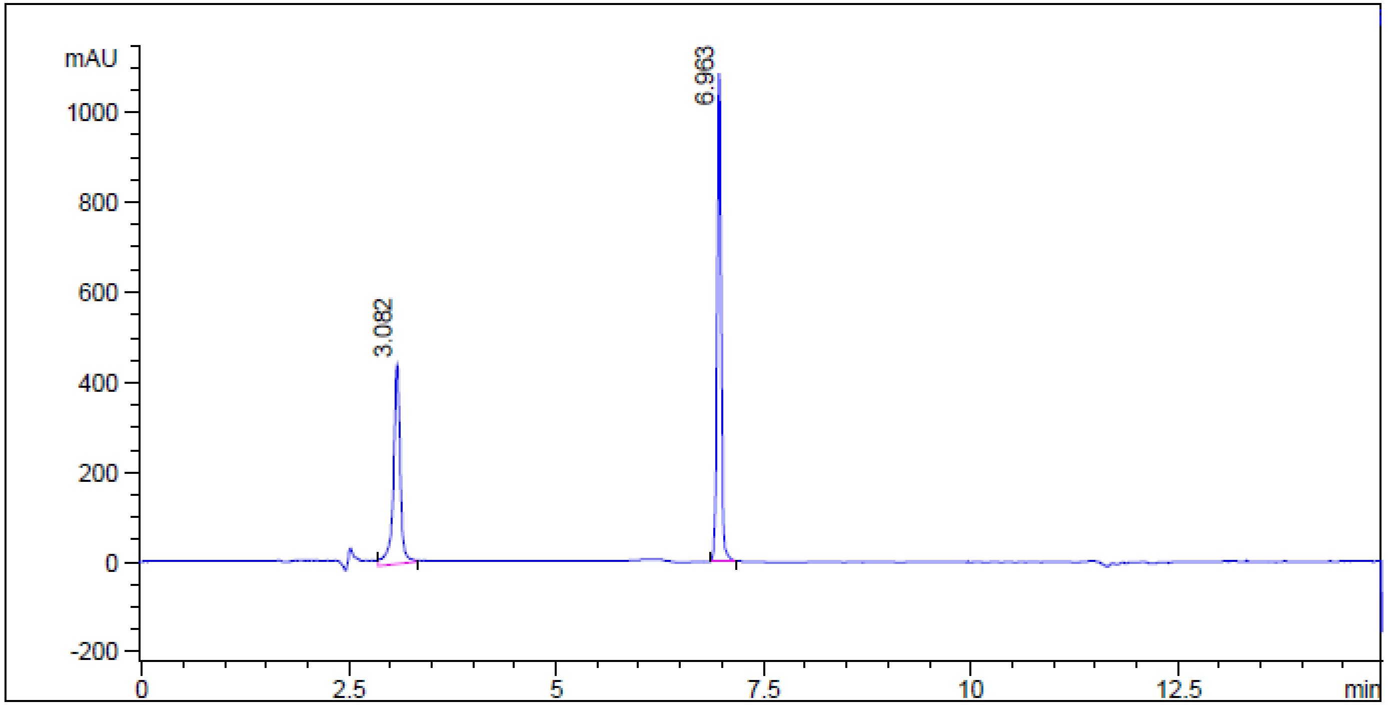

Area Percent Report

Signal 1: DAD1 A, Sig=220,16 Ref=off

Peak #	RT [min]	Type	Width [min]	Height	Area	Area %
1	3.082	BB	0.086	28.926	2594.402	40.346
2	6.963	BB	0.055	71.074	3835.935	59.654

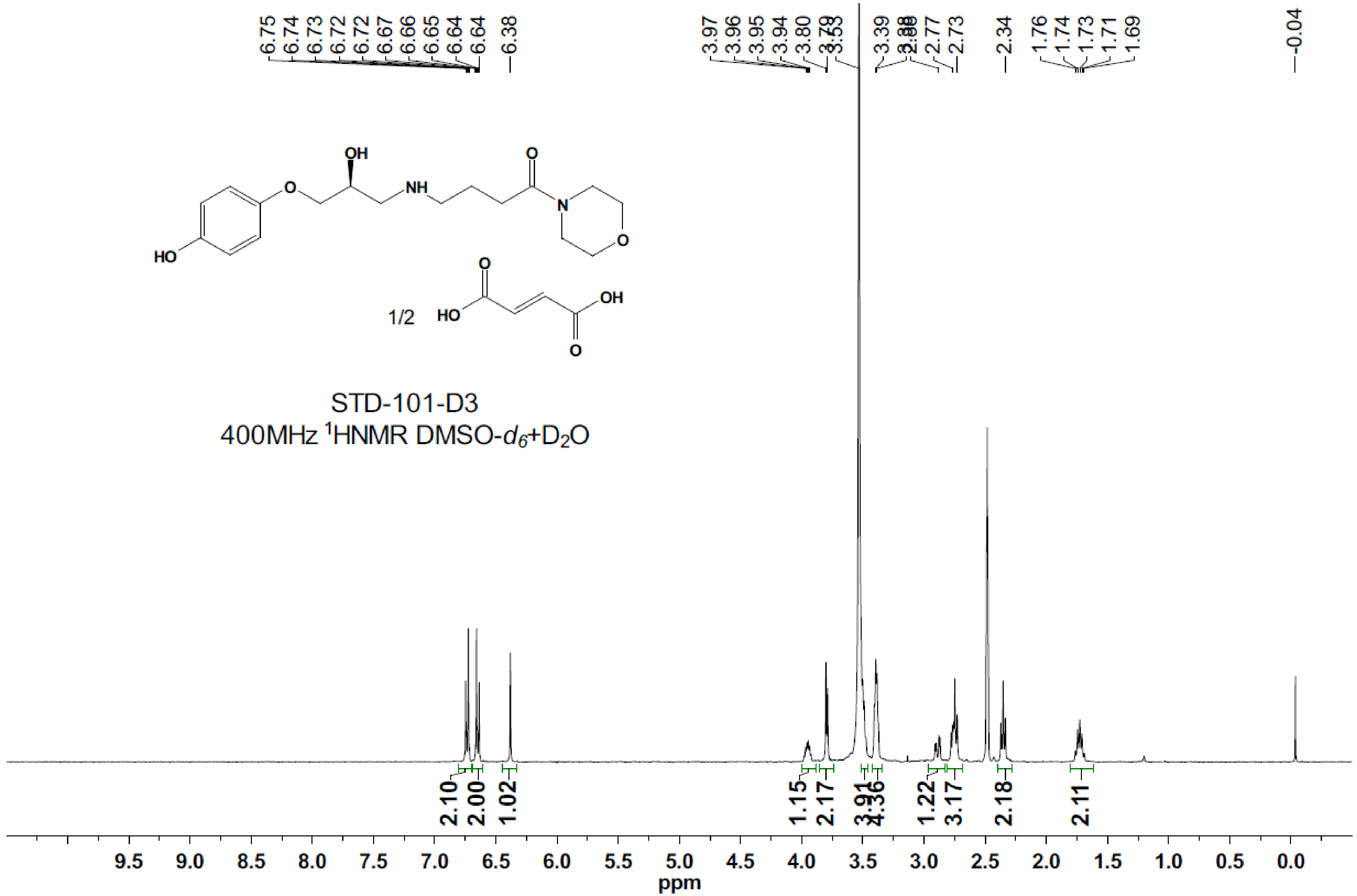

STD-101-D3
400MHz 1HNMR DMSO-d6+D2O
1/2
OH
NH
O
N
HO
6.75
6.74
6.73
6.72
6.72
6.67
6.66
6.65
6.64
6.64
6.38
3.97
3.96
3.95
3.94
3.80
3.39
2.77
2.73
2.34
1.76
1.74
1.73
1.71
1.69
-0.04
2.10
2.00
1.02
1.15
2.17
1.22
3.17
2.18
2.11
9.5
9.0
8.5
8.0
7.5
7.0
6.5
6.0
5.5
5.0
4.5
4.0
3.5
3.0
2.5
2.0
1.5
1.0
0.5
0.0
ppm

STD-101-D4

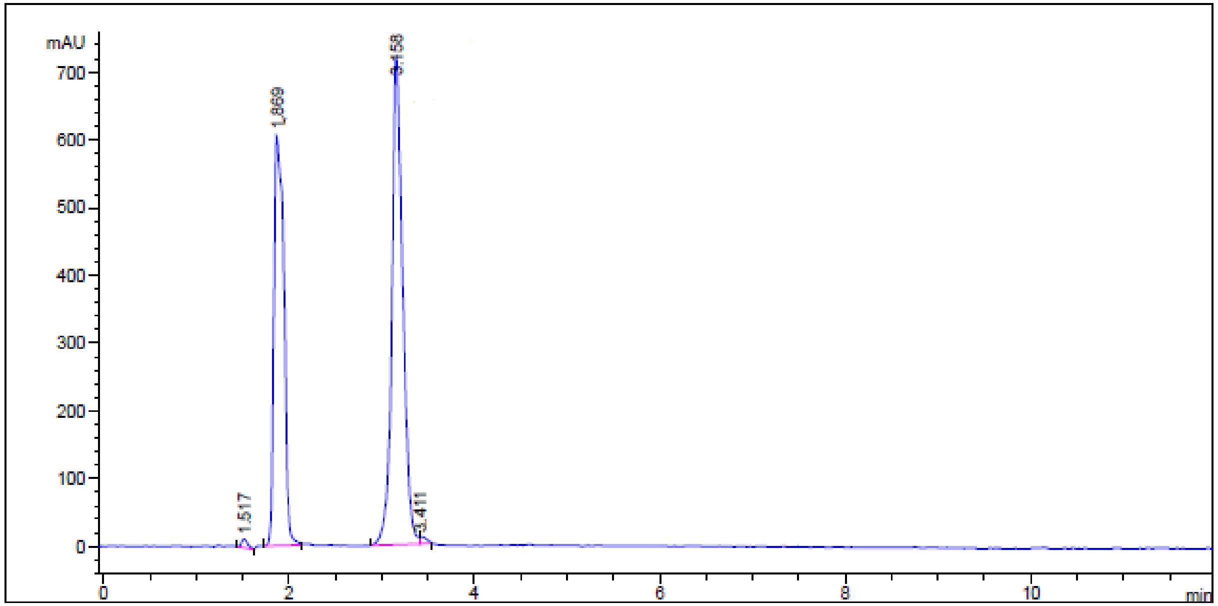


```
=====================================================================
                       Area Percent Report
=====================================================================

Signal 1: DAD1 A, Sig=220,4 Ref=off
 Signal has been modified after loading from rawdata file!

Peak RetTime Type  Width     Area      Height     Area
  #   [min]        [min]   [mAU*s]     [mAU]        %
----|-------|----|-------|----------|----------|--------|
   1   1.517 BV    0.0678   55.64667   12.53394   0.5295
   2   1.869 MM    0.1237 4505.24902  607.06256  42.8670
   3   3.158 MF    0.1366 5901.18799  719.83838  56.1492
   4   3.411 FM    0.0794   47.75102   10.02919   0.4543

Totals :                  1.05098e4  1349.46407

=====================================================================
```

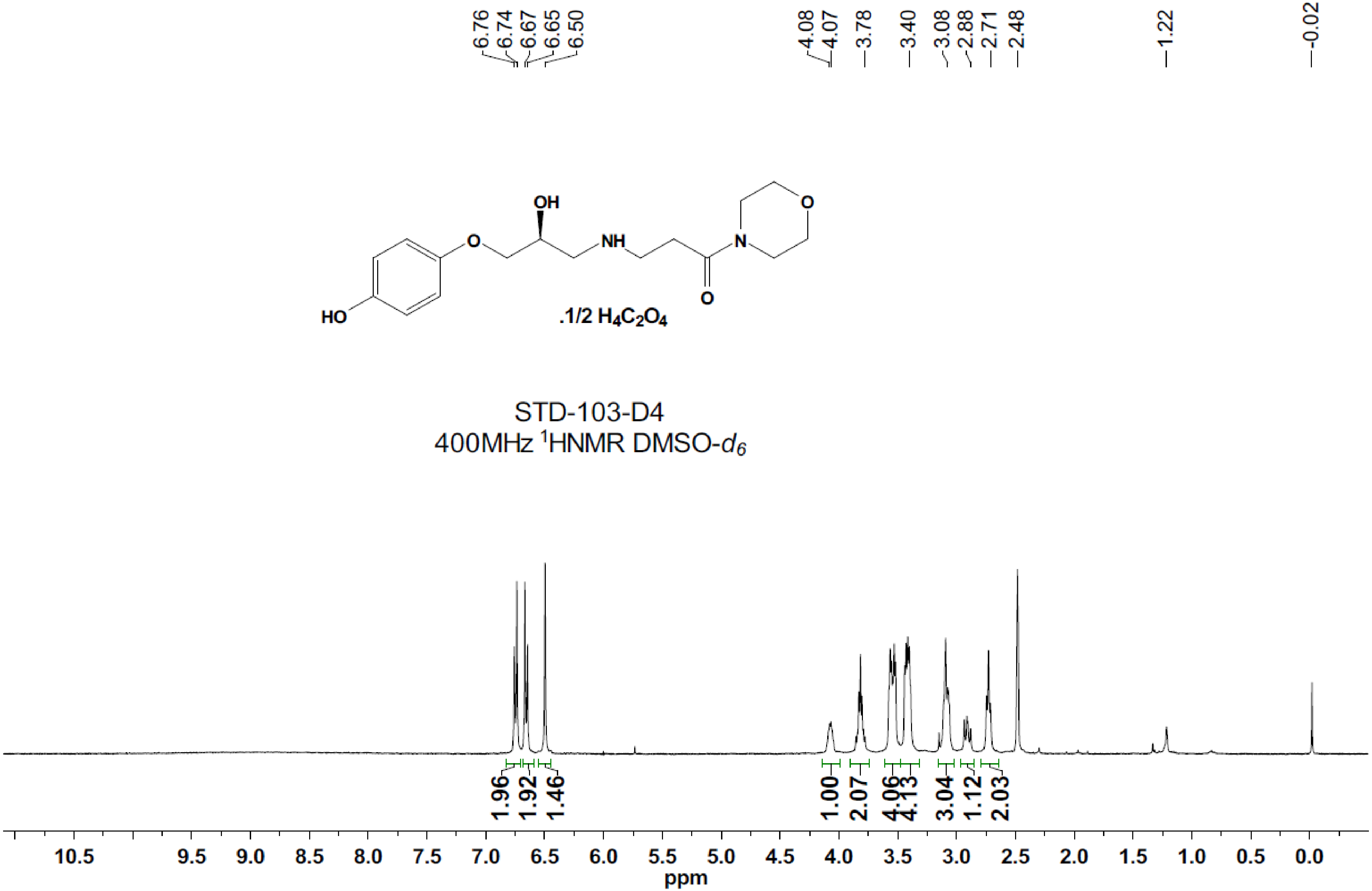

STD-103-D4

400MHz ^{1}HNMR DMSO-d_6

STD-101-D5

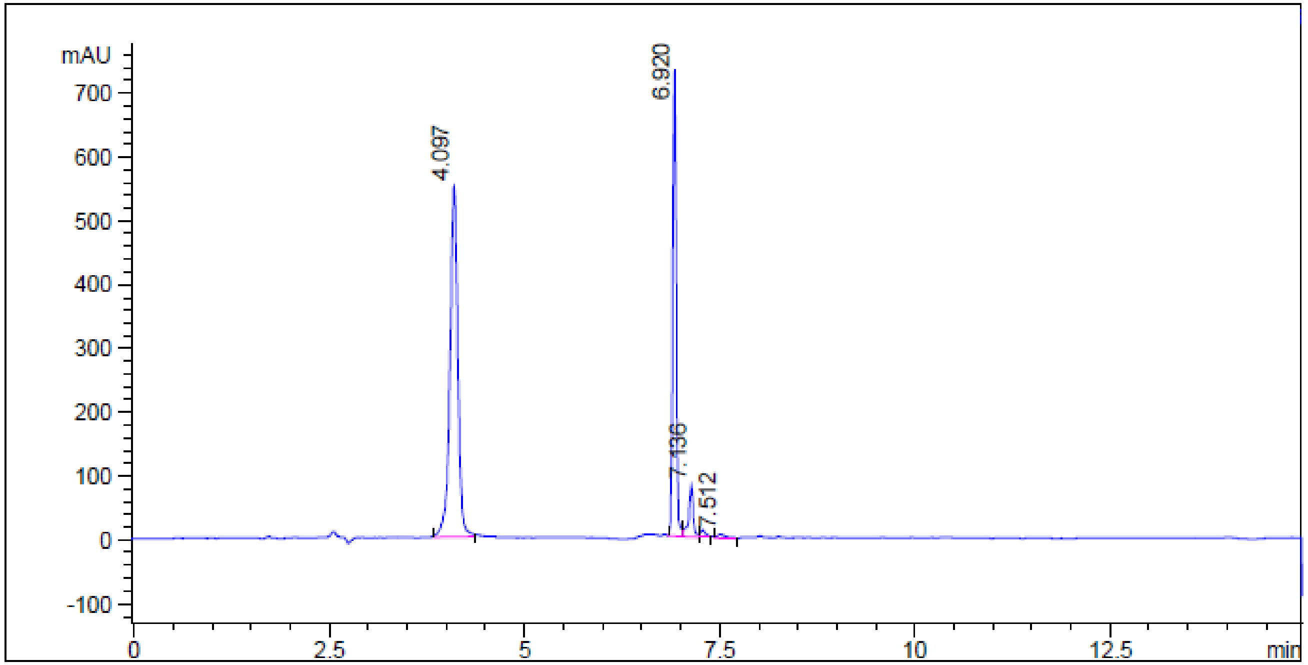

Area Percent Report

Signal 1: DAD1 A, Sig=220,16 Ref=off

Peak #	RT [min]	Type	Width [min]	Height	Area	Area %
1	4.097	BB	0.109	39.793	3890.943	58.817
2	6.920	BV	0.051	53.120	2302.392	34.804
3	7.136	VB	0.059	5.819	321.795	4.864
4	7.283	BB	0.066	0.864	55.078	0.833
5	7.512	BB	0.108	0.405	45.119	0.682

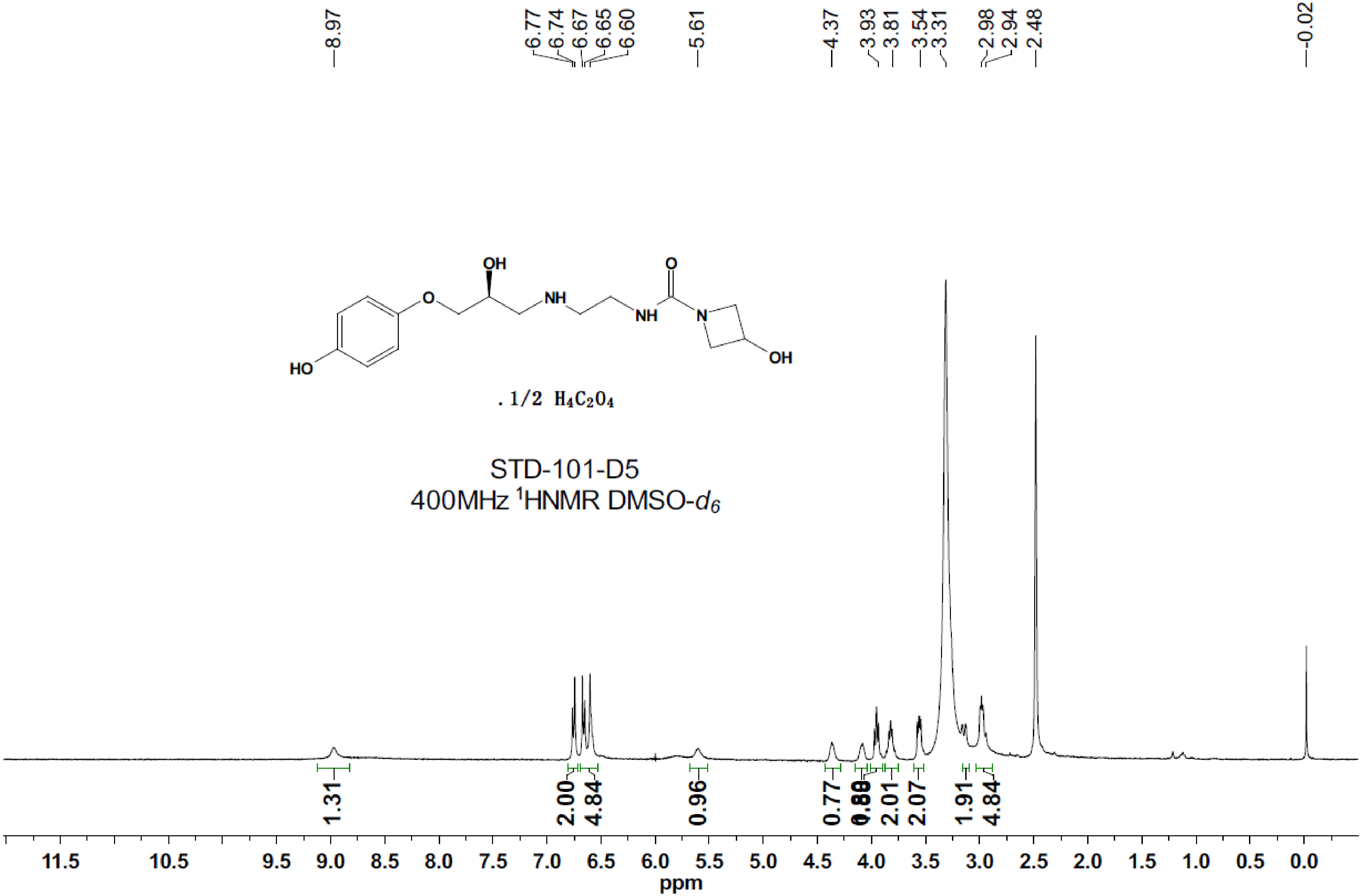
8.97
6.77
6.74
6.67
6.65
6.60
5.61
4.37
3.93
3.81
3.54
3.31
2.98
2.94
2.48
-0.02
OH
O
NH
NH
N
O
HO
OH
. 1/2 H4C2O4
STD-101-D5
400MHz 1HNMR DMSO-d6
1.31
2.00
4.84
0.96
0.77
2.01
2.07
1.91
4.84
11.5
10.5
9.5
9.0
8.5
8.0
7.5
7.0
6.5
6.0
5.5
5.0
4.5
4.0
3.5
3.0
2.5
2.0
1.5
1.0
0.5
0.0
ppm

STD-101-D6

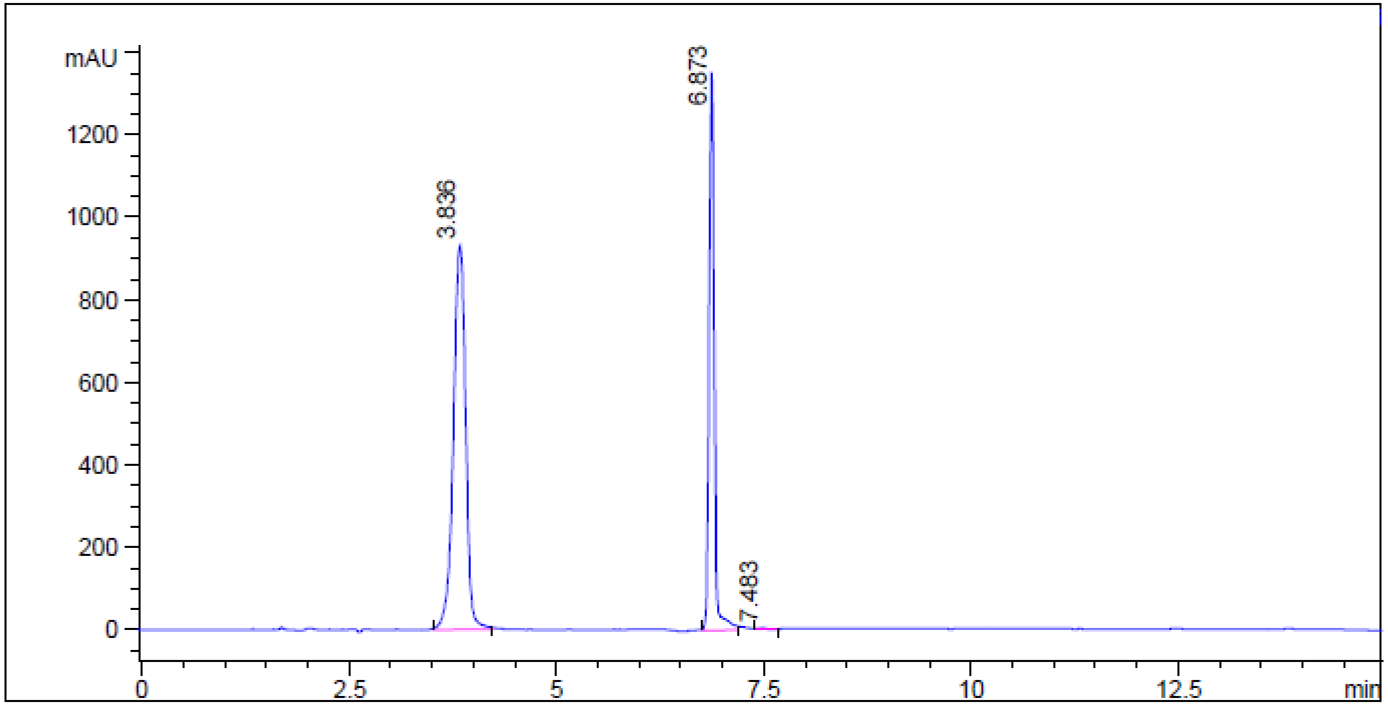

===

Area Percent Report

===

Signal 1: DAD1 A, Sig=220,16 Ref=off

Peak #	RT [min]	Type	Width [min]	Height	Area	Area %
1	3.836	BB	0.162	40.577	9582.307	62.208
2	6.873	BB	0.068	59.261	5796.280	37.629
3	7.483	BB	0.093	0.162	25.003	0.162

===

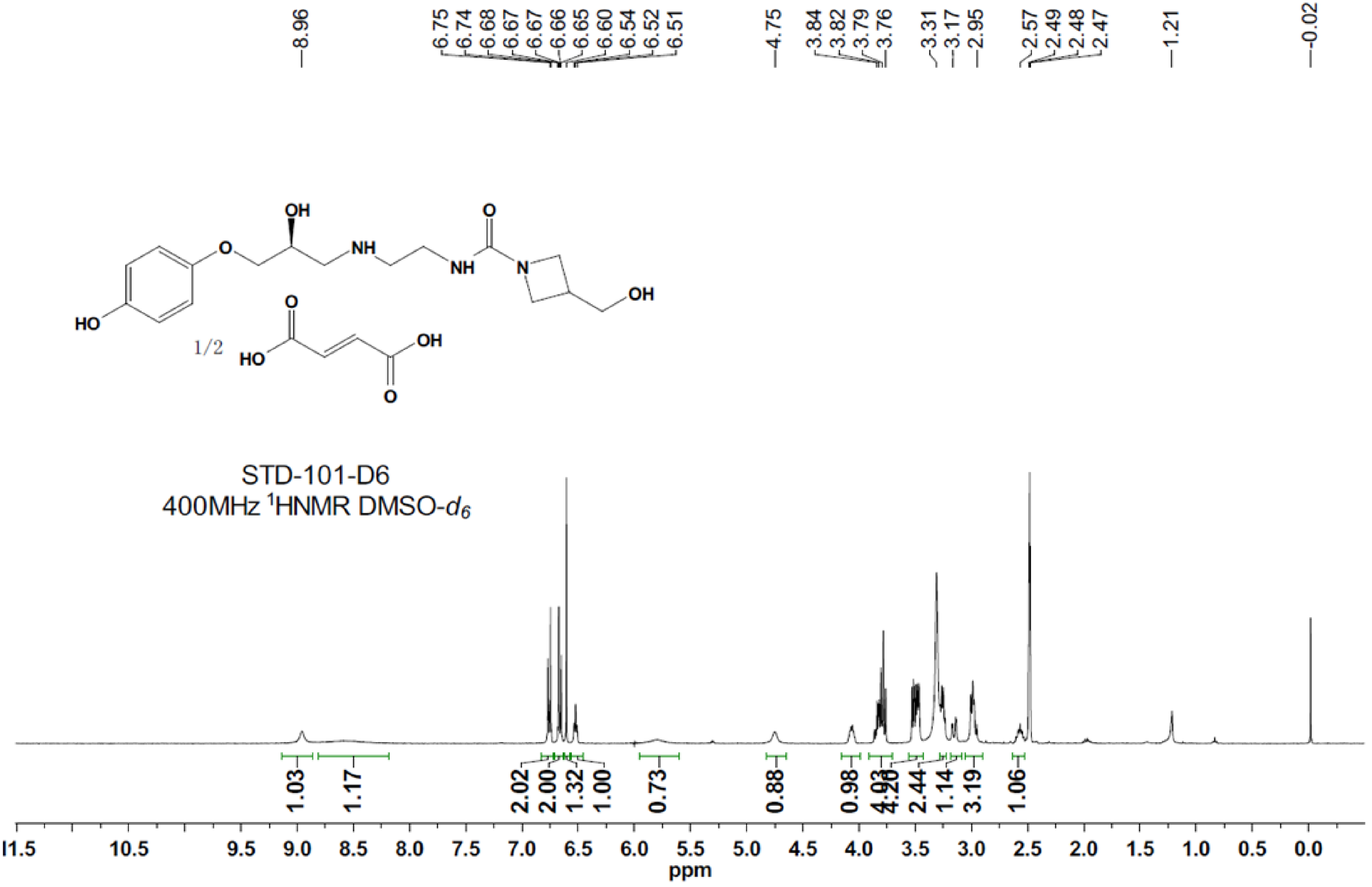
STD-101-D6
400MHz 1HNMR DMSO-d6
1/2
ppm
