# Supplementary material for: Discovery of novel brain permeable and G protein-biased beta-1 adrenergic receptor partial agonists for the treatment of neurocognitive disorders
Source: PLoS One. 2017 Jul 26;12(7):e0180319. doi: 10.1371/journal.pone.0180319 (PMC5529018; doi:10.1371/journal.pone.0180319)
Supplement: S2 Table — (PDF) [file pone.0180319.s003.pdf]

S2 Table. Binding affinity (K_i, nM) of STD-101-D1 at non-ADRB1 binding sites.

Receptor	Hot ligand	Binding Affinity K_i (nM)
ADRB1	[^{125}I]Pindolol	229.7
ADRB2	[^{3}H]CGP12177	-
ADRB3	[^{3}H]CGP12177	484.7
ADR- Alpha 1A	[^{3}H] Prazosin	17
ADR- Alpha 1B	[^{3}H] Prazosin	267
ADR - Alpha 1D	[^{3}H] Prazosin	25
ADR - Alpha 2A	[^{3}H] Rauwolscine	1168
ADR - Alpha 2B	[^{3}H] Rauwolscine	337
ADR - Alpha 2C	[^{3}H] Rauwolscine	108
5-HT 1A	[^{3}H]8-OH-DPAT	11
5-HT1B	[^{3}H]GR125743	382
5-HT 1D	[^{3}H]GR125743	149
5-HT 1E	[^{3}H]5HT	-
5-HT 2A	[^{3}H]Ketanserin	1239
5-HT 2B	[^{3}H]LSD	25
5-HT 2C	[^{3}H]Mesulergine	1153
5-HT 3	[^{3}H]GR65630	-
5-HT5A	[^{3}H]LSD	-
5-HT6	[^{3}H]LSD	1769
5-HT7	[^{3}H]LSD	-
PBR#	[^{3}H]PK11195	-
D_1	[^{3}H]SCH23390	-
D_2	[^{3}H]N-Methylspiperone	-
D_3	[^{3}H]N-Methylspiperone	47
D_4	[^{3}H]N-Methylspiperone	
D_5	[^{3}H]SCH23390	-
DAT	[^{3}HWIN35428	-
NET	[^{3}H]Nisoxetine	-
δ-OR, μ-OR, κ-OR	[^{3}H]DADLE, [^{3}H]DAMGO, [^{3}H]U69593	-
GABA A receptor	[^{3}H] Muscimol	-
H_1	[^{3}H]Pyrilamine	-
H_3	[^{3}H]Alpha-methylhistamine	-
H_4	[^{3}H]Histamine	-
M_1, M_2, M_3, M_4, M_5 mAChR	[^{3}H]QNB	-
Sigma 1 receptor	[^{3}H]Pentazocine(+)	146
Sigma 2 receptor	[^{3}H]DTG	236

_ < 50% inhibition of radioligand binding at 10 μM STD-101-D1

Receptor abbreviations:
ADR, adrenergic receptor; 5HT, serotonin receptor; PBR, peripheral benzodiazepine receptor; D, dopamine receptor; DAT, dopamine transporter; NET, norepinephrine transporter; OR, opioid receptor; GABA, *gamma*-aminobutyric acid receptor; H, histamine receptor; mAChR, muscarinic acetylcholine receptor.
